# Investigating subtle changes in facial expression to assess acute pain in Japanese macaques

**DOI:** 10.1038/s41598-022-23595-x

**Published:** 2022-11-16

**Authors:** Vanessa N. Gris, Nelson Broche, Akihisa Kaneko, Munehiro Okamoto, Juri Suzuki, Daniel S. Mills, Takako Miyabe-Nishiwaki

**Affiliations:** 1 Primate Research Institute, Kyoto University, Inuyama, Japan; 2 Center for the Evolutionary Origins of Human Behavior, Kyoto University, 41-2 Kanrin, Inuyama, Aichi 484-8506 Japan; 3 Department of Life Sciences, University of Lincoln, Lincoln, UK

**Keywords:** Animal behaviour, Pain

## Abstract

Changes in facial expression provide cues for assessing emotional states in mammals and may provide non-verbal signals of pain. This study uses geometric morphometrics (GMM) to explore the facial shape variation in female Japanese macaques who underwent experimental laparotomy. Face image samples were collected from video footage of fourteen macaques before surgery and 1, 3, and 7 days after the procedure. Image samples in the pre-surgical condition were considered pain-free, and facial expressions emerging after surgery were investigated as potential indicators of pain. Landmarks for shape analysis were selected based on the underlying facial musculature and their corresponding facial action units and then annotated in 324 pre-surgical and 750 post-surgical images. The expression of pain is likely to vary between individuals. Tightly closed eyelids or squeezed eyes and lip tension were the most commonly observed facial changes on day 1 after surgery (p < 0.01974). A good overall inter-rater reliability [ICC = 0.99 (95% CI 0.75–1.0)] was observed with the method. The study emphasizes the importance of individualized assessment and provides a better understanding of facial cues to pain for captive macaque care.

## Introduction

Non-human primates have been extensively studied for biomedical research purposes due to their close evolutionary relationship to humans^1^. Ensuring high welfare standards is not only morally important and legally mandated, but also avoids jeopardizing data validity from changes such as the lack of adequate immobilization and sympathetic response associated with surgery^2^. The assessment of pain is a key component of a welfare plan. Preventing and alleviating pain, together with distress and other adverse effects experienced, is a core aspect of the refinement principle of the 3Rs for animal experimentation^3^. Pain is recognized as a multidimensional experience involving physiological, behavioural and highly subjective components. The gold standard for assessing pain intensity in humans is typically verbal self-reporting. However, populations that need special care (human patients who are non-verbal and/or have cognitive or communication impairments)^4^, babies and children, and non-human animals are not able to verbalize this emotional state. Therefore, different pain assessment approaches and behavioural cues that do not depend on speech need to be developed.

Pain assessment during acute invasive procedures in domesticated species has been widely reported. Despite progress, the difficulties inherent to the process of discriminating the presence of pain and its intensity in wild species, such as macaques is aggravated by the lack of a standardized measurement scale. Reliance solely on physiological indicators can be misleading as prolonged anxiety, fear, and drug treatment as well as captive conditions, reproductive cycle, and circadian decline are not necessarily linked to pain but can alter key reference parameters such as adrenaline and cortisol levels^5–8^. Common health parameters measured in pain scales, such as temperature, heart rate, respiratory rate^7^ and blood pressure cannot, in most cases, be evaluated without direct handling in non-human animals. Furthermore, in the case of non-domesticated animals, such as non-human primates, direct handling presents an increased risk of injury for both the subject and health management team. Palpation of the painful area in cynomolgus macaques (*Macaca fascicularis*), for example, is performed only under anaesthesia, a procedure that greatly impacts spontaneous expressions of pain^9^. The Association of Primate Veterinarians’ Guidelines for Assessment of Acute Pain in Nonhuman Primates does not recommend palpation of the painful site in a conscious nonhuman primate unless the animal is trained to cooperate^10^.In this context, assessing behaviour could indicate pain more adequately than simple physiological measures^11^. However, the manifestation of observable behaviours after a painful event in non-human primates may not be obvious or may be suppressed, especially in the presence of an observer^11^. This may partially explain why means of assessing pain and analgesic regimen are poorly reported and the development of instruments to assess pain in non-human primates is urgent and necessary^12,13^.

Facial expressions provide a source of nonverbal communication in social interactions in many group-living mammalian species. As observed by Descovich et al. facial expressions can determine generalized, species-specific patterns, as well as accommodate individual variation^23^. The importance of the face for recognition of pain has been acknowledged in humans, especially neonates, children, and those who have become debilitated from conditions such as dementia^14,15^. The human pain face presents consistent changes across different stimuli and may be translated into brow lowering and tightening, closing of the eye, nose wrinkling and upper lip raising^16^. Recently, evaluation of facial expressions in animals have been incorporated into multidimensional pain instruments. For example, grimace scales with facial expressions considered to be associated with pain have been developed for domesticated species^17–20^. Although audience and directed attention are shown to affect the production of facial expressions in non-human primates and dogs, facial cues may potentially indicate emotional experiences determining generalized patterns as well as accommodating individual variation^21–24^.

Geometric morphometrics (GMM) is a fairly simple and relatively recent approach applied to the study of facial morphology^25–29^. GMM is based on the multivariate analysis of anatomical-based landmarks as Cartesian coordinates (i.e. x, y) to capture information of shape while maintaining its relative spatial arrangements. In GMM, the main measure of difference is the Procrustes distance, a distance between shapes after each shape has been adjusted until the sum of squared distances among them is minimized. This process is known as Procrustes fitting, Procrustes superimposition or Generalized Procrustes Analysis (GPA). GMM is applicable to shape analysis in many biologically structures for studies of development and evolution, being able to capture subtle morphological differences between species or populations of interest^30^. For example, regarding pain, GMM was suitable for confirming the discrimination of painful and pain free conditions in cats by the analysis of facial components^26^.

At present, the use of a geometric morphometric approach has not been investigated to study facial shape variation in macaques. The aim of this study was to evaluate the changes in the facial expression of macaques at four different time points related to laparotomy: pre-operatively, then 1, 3 and 7 days after surgery, using GMM. We hypothesized that macaques would display changes in facial expressions after surgery (pain present, especially one day after surgery) compared to the pre-operative condition (pain-free).

## Methods

### Subjects

This research was approved by the Animal Welfare and Care Committee of the Primate Research Institute, Kyoto University (#2016-109, #2017-096, #2018-178, #2019-156, #2020-050). All methods were performed in accordance with the relevant guidelines and regulations and reported according with ARRIVE guidelines. No surgery was conducted for the purposes of the present study and data on pain were collected opportunistically. Fourteen female Japanese macaques (*Macaca fuscata*), aged 9 ± 4 years and weighing 7.7 ± 1.2 kg were enrolled in the study. All animals underwent the invasive procedures in relation to reproductive biology studies. Macaques were housed in pairs in metal cages (D650 mm × W1560 mm × H800 mm). Approximately ten macaques were housed in each air-conditioned room, maintained at 20–27 °C and in a 12:12 light–dark cycle. Feeders, wooden toys, swings and radio music were provided as environmental enrichment. Macaques were fed twice daily with monkey chow (approximately 200 g) and sweet potatoes three times per week. Fresh water was provided ad libitum by an automatic watering system. Occasional treats such as dried banana, peanuts, fresh apples and bananas were offered as positive reinforcement for procedures such as recording their weight, after medication and in video recording sessions.

### Surgical procedure

Macaques underwent laparotomy for oocyte retrieval or implantation. Twenty-five surgical procedures were recorded between 2016 and 2020. Surgeries were performed between 09:00 and 11:00. Food, but not water, was withheld the evening before procedure. The procedure consisted of a midline abdominal incision through the skin, fascia and musculature. The uterus and ovaries were manipulated according to the surgery. Subjects were anesthetized with a combination of ketamine (5 mg/kg, Daiichi Sankyo, Propharma, Tokyo, Japan), medetomidine (0.025 mg/kg, Medetomin injection Meiji, Meiji Seika Pharma Co. Ltd., Tokyo) and midazolam (0.125 mg/kg, Midazolam Injection SANDOZ, Sandoz K. K., Tokyo) intramuscularly (IM) using a squeezing cage. Anaesthesia was maintained with sevoflurane in 100% O_2_ delivered by face mask. Amoxicillin (15 mg/kg, Amostac, Meiji Seika Pharma Co. Ltd., Tokyo), famotidine (0.1 mg/kg), buprenorphine (0.01 mg/kg, Lepetan, Otsuka Pharmaceutical Co., Ltd., Tokyo) and carprofen (4 mg/kg, Rimadyl, Zoetis Japan, Tokyo) were also administered. Heart rate and rhythm, respiratory rate, non-invasive blood pressure, haemoglobin saturation (SpO_2_%) and end-tidal carbon dioxide (EtCO_2_) were monitored with a multiparametric monitor (BP-608EV, Omron-Colin, Tokyo). Following surgery, animals recovered in individual cages in a hospital wing and returned to their original pair cage room. In addition to antibiotic and antiemetic treatment, analgesics were administered from the day of the surgery through day 3. Animals received an intramuscular injection of buprenorphine (0.01 mg/kg, BID), a subcutaneous injection of carprofen (4 mg/kg, SID), amoxicillin (15 mg/kg, IM, SID) and famotidine (0.1 mg/kg, IM, SID). Medication was administered after the video recording session (0900) and the second dose of buprenorphine was administered at the end of the workday (1800). Animals did not receive medication from days 4 through 7.

### Image sample collection

Facial images were captured from video footage of the macaques in four separate conditions: before surgery (Pre), before analgesic medication in the morning of days 1 (D1) and 3 (D3) and on the morning of day 7 (D7). Thirty to fifty-minute video sequences were recorded using cameras (GoPro HERO6Black, HERO7Black, HERO8Black) protected by a plastic box and attached to the cage bars. The camera lenses were positioned between bars to avoid visual blocking in the recording area. After setting up the equipment, the observer left the room and was not visible or audible to the macaques. Video recording aimed to capture images at the time considered to have minimal analgesic benefit. While there is no published data in macaques regarding the therapeutic plasma buprenorphine concentration, 0.16 ng/mL has been identified as a threshold in dogs^31^. At a hypothesized analgesic concentration threshold of 0.1 ng/mL, buprenorphine at 0.01 mg/kg IM is likely to provide analgesia for 10.8 ± 4.5 h^32^. Thus, the minimal analgesic period was recorded prior to the morning analgesic injection. Pre was categorized as the baseline, assuming the individual was pain-free and facial expressions emerging after surgery were investigated as potential indicators of pain, it was assumed that the individual’s pain level would be the highest on D1, whilst for days 3 and 7, it was assumed that pain would be reduced or absent.

All video snapshots meeting criteria for annotation were extracted, with an inter-sample interval of at least 2 s. Frontal views and images with the head slightly rotated were included for annotation, where all the landmark positions were visible. A total of 1074 individual images from fourteen macaques who underwent laparotomy were selected. Images were cropped from the neck up and standardized using GIMP (v.2.10.14), leaving only the facial area available for annotation and excluding the body where clipped hair and the surgical wound could indicate the animal’s status.

### Geometric morphometrics

**Figure 1:**
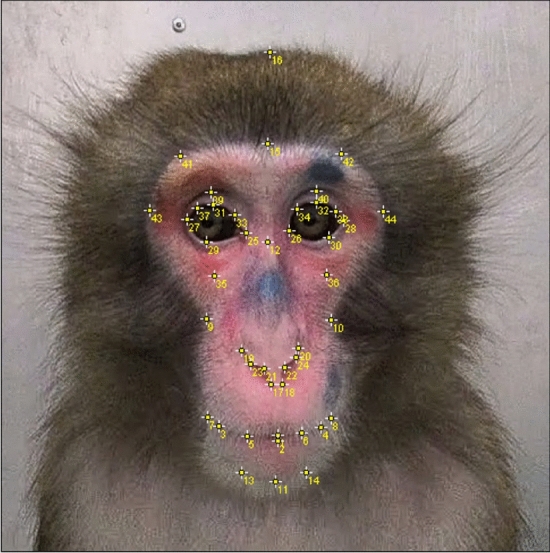
Anatomical landmarks and descriptions. Landmarks (n = 44) were manually annotated in facial images of Japanese macaques for shape analysis. For more information about the landmarks and their placement, see Supplementary Data S1.

Forty-four facial landmarks were annotated to capture a wide range of variation in facial expression using ImageJ (v.1.53e) by VG. Landmarks were selected based on the underlying facial musculature and their corresponding facial action units identified in rhesus macaques (*Macaca mulatta*)^33^, and later confirmed with the extension of MaqFACS for Japanese macaques^34^ (see Supplementary Data S1). On each image, we digitized 44 bi-dimensional landmarks for shape analysis (Fig. 1). All landmarks were present in all faces annotated.

**Table:** 

LM	Description
	**Mouth region**
1	Central point of upper lip
2	Central point of lower lip
3	Mouth corner (lips most external point) (r)
4	Mouth corner (lips most external point) (l)
5	Lip midpoint between LM 1 and LM 3 (r)
6	Lip midpoint between LM 1 and LM 4 (l)
7	The boundary between skin and coat following the mouth line (LM5, LM3) (r)
8	The boundary between skin and coat following the mouth line (LM6, LM4) (l)
	**Cheek**
9	Inner tip of the face outline (r) (the boundary between skin and coat)
10	Inner tip of the face outline (l) (the boundary between skin and coat)
	**Mental region**
11	Ventral edge of jaw at midline (the boundary between skin and coat)
13	Midpoint between LM 11 and LM 3 (r) (the boundary between skin and coat)
14	Midpoint between LM 11 and LM 4 (l) (the boundary between skin and coat)
	**Forehead/Browridge**
15	Ventral edge of forehead at midline (the boundary between skin and coat)
16	Central point of the head top coat (the boundary between coat and external environment)
	**Nose**
12	Lower bound of vertical nose ridge at midline
17	The bottom point of inner right nostril (right edge of the septum) (r)
18	The bottom point of inner left nostril (left edge of the septum) (l)
19	The top of right nostril (r)
20	The top of left nostril (l)
21	The tip point on inner nostril edge (r)
22	The tip point on inner nostril edge (l)
23	Nose tip midpoint on upper nostril edge, between LM 21 and MLM 19 (r)
24	Nose tip midpoint on upper nostril edge, between LM 22 and LM 20 (l)
	**Eye**
25	Medial corner of the eye (r)
26	Medial corner of the eye (l)
27	Lateral corner of the eye (r)
28	Lateral corner of the eye (l)
29	Lower eyelid (point where horizontal portion goes upwards) (r)
30	Lower eyelid (point where horizontal portion goes upwards) (l)
31	Edge of the upper eyelid, at the midpoint (r)
32	Edge of the upper eyelid, at the midpoint (l)
33	Midpoint between LM 25 and LM 31 (eyelid outline) (r)
34	Midpoint between LM 26 and LM 32 (eyelid outline) (l)
37	Midpoint between LM 27 and LM 31 (eyelid outline) (r)
38	Midpoint between LM 28 and LM 32 (eyelid outline) (l)
39	Midpoint of the upper line of double eyelids (r)
40	Midpoint of the upper line of double eyelids (l)
	**Infraorbital furrow**
35	Bottom point of the periorbital puffiness (r)
36	Bottom point of the periorbital puffiness (l)
	**Browridge**
41	Outline of the browridge (boundary between skin and coat in forehead), imaginary line from LM 25 through LM 39 (r)
42	Outline of the browridge (boundary between skin and coat in forehead), imaginary line from LM 26 through LM 40 (l)
43	Inner tip of the outline of the face (boundary between skin and coat in face), imaginary line from LM 25 through LM 27 (r)
44	Inner tip of the outline of the face (boundary between skin and coat in face), imaginary line from LM 26 through LM 28 (l)

To confirm inter-rater reliability of annotation, a second rater (NB) coded approximately 20% of images randomly selected from the dataset, using an example image and the description of each landmark as reference. Observers were blind to the condition from which the image was captured. ICC estimates and corresponding 95% confidence intervals were calculated using the ‘psych’ package in R (v.3.6.3), based on a single rater measurement, absolute agreement, two-way random effects model^35^.

During annotation, a general visual inspection of the images was performed to provide additional information. This evaluation aimed to identify any obvious changes from a neutral expression.

#### Group analysis

First, a group-level analysis was performed to explore broader changes and check for consistent differences across conditions. For that, we generated a balanced dataset in which individual macaques and their associated data contributions were pseudo- randomly selected and pooled to ensure even distribution of observations (up to 6 observations per condition per individual, n = 308; see Supplementary Data S2).

Shape information was extracted from the landmark coordinates with a generalized Procrustes fit using MorphoJ (v.1.07a)^36^. Landmark configurations were superimposed by translating, rotating and scaling all configurations to a common reference system (the mean). The resulting multidimensional geometric information was summarized using a Principal Component Analysis (PCA). PCA was applied to reduce the data and create new components to maximise the description of the total variation in the dataset in an ordered way, with each successive component describing smaller proportions of variance. PCA of the shape variables of the group-level analysis indicated that the first, second and third principal components (PC1, PC2 and PC3) were particularly associated with head-turning (yawing), “nodding” (pitching) or a combination of both. Biological 3D structures can be rendered into 2D representations (images) and the position of the head varies depending on the point of view (i.e., subject orientation with respect to the camera). In the case of frontal view images of faces, GMM controls for the variation in head tilting (rolling), but not for head-turning and nodding^37^. One approach to remove the artificial variation of orientation is to use regression before comparing groups. Therefore, to mitigate the effects of subject orientation we calculated the residuals of the within-group pooled regression between Procrustes coordinates and the respective PCs^38^. The resulting dataset containing the residuals produced by the regression was used to generate a covariance matrix and a new PCA was then performed. To further explore group differences, we used discriminant analysis. Canonical Variate Analysis (CVA) was used for visualization of the shape features that best distinguish between the four conditions and a confusion matrix provided information on the accuracy of the classification. Statistical results were considered significant at p < 0.05. The error correction and generation of wireframes were performed with MorphoJ while the data formatting for ICC and MorphoJ input was done using custom Bash and R scripts.

Average PC scores were generated for each of the PCs, for each macaque, across each condition. One-way repeated measures ANOVAs, with post-hoc Tukey tests, and Bonferroni corrected p values were used with images collected in all conditions (12 of 14 animals) to identify the source of any significant differences in PC scores across the conditions using PAST4 (v.4.06b)^39^.

#### Individual analysis

Data from single individuals were analysed across the conditions aiming to capture the repertoire of expressions that may not be observed in all animals due to the subjective nature of pain. For that, we used the full dataset of images available for each individual (n = 1074; 14–154 images per individual, see Supplementary Data S2). We performed the same analysis as in the group-level analysis using individual data, i.e., the Procrustes analysis, corrected the resulting PCA and a discriminant analysis. A discriminant analysis was performed for each subject individually, excluding inter-subject morphological variation.

## Results

A total of 1074 individual images across the four conditions were extracted from the fourteen macaque subjects who underwent laparotomy (Supplementary Data S2). Inter-rater agreement was tested for each of the 88 x–y coordinates to indicate whether both coders were able to reliably identify the landmark placements. The coders’ overall reliability from a first round of annotation indicated an inter-rater agreement of 0.73 to 0.99. Although the ICC values indicate moderate to excellent reliability, its 95% interval confidence was variable with a level of reliability regarded as poor to excellent. To improve the annotation of landmark position, the descriptions of inconsistent coordinates were reviewed, rewritten and followed by a second round of annotation, resulting in ICC ranging between 0.93 and 0.99, with a median value of 0.99 and good to excellent confidence intervals (see Supplementary Data S3).

### Visual inspection

**Figure 2:**
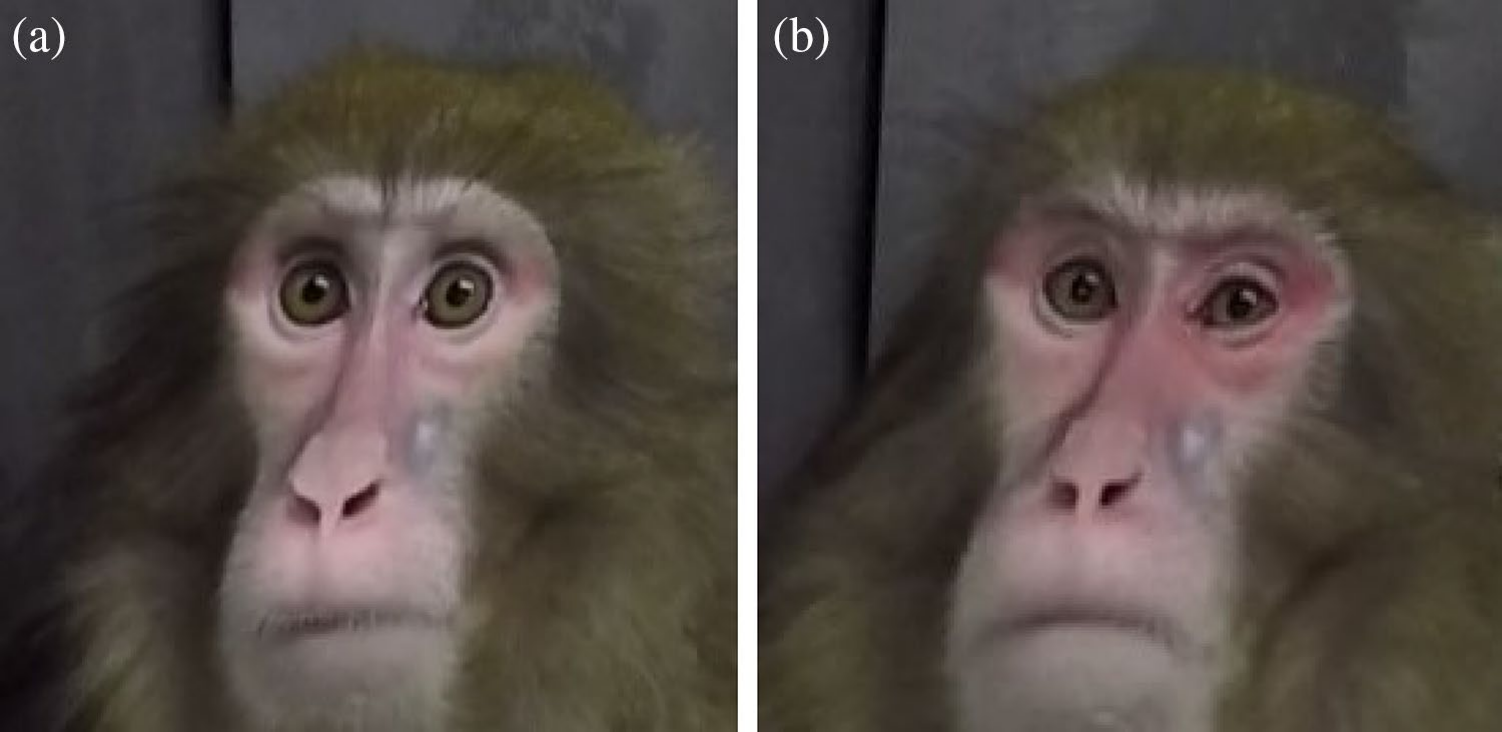
An example of eyelid asymmetry in a female Japanese macaque before and after laparotomy. (**a**) Pre: the subject is alert, presenting relatively symmetrical facial features. (**b**) D1: the picture was taken before scheduled analgesia. The left eye is more closed than the right eye.

Some facial changes were subtle in appearance and challenging to detect by direct observation alone. The most obvious difference in detecting pain between pre and post-surgery conditions was observed in some individuals the day following surgery. In these individuals, the D1 condition showed changes in the eyes where the eyelids became asymmetrical or when symmetry was found the eyelids were half open (Fig. 2). It was also observed that some animals occasionally presented the lips parted on the first day after surgery while restful (i.e., not eating or using the mouth for any intentional activity). As facial expressions are dynamic, individuals did not present these appearances during the whole recording time.

### Dilution of facial cues in group analysis

Group analysis showed remarkable dilution in facial cues. To evaluate the extent of variation in overall facial shape in post-surgery conditions, we performed a generalized Procrustes analysis on 308 facial samples. The variability in the shape space was assessed using a PCA. PCA showed substantial superimposition between Pre, D1, D3 and D7. The first 22 principal components (PC) explained 91% of the variance in the dataset (from 25.4% [PC1] to 0.7% [PC22]). We found that the largest differences in facial morphology were associated with the normal intrinsic anatomical differences that occur among female Japanese macaques. A confusion matrix was generated during discriminant analysis and showed that a classification accuracy of 91.9% (picture matching to the correct subject) was due to the underlying neutral facial morphology. The classification accuracy due to differences in individual pain condition was 38.6%. (Supplementary Data S3).

Twelve macaques had extractable images across all four conditions. Across the twenty-two PC components tested, only PC13 (F_3,44_ = 3.95, p < 0.01644) and PC19 (F_3,44_ = 4.547, p < 0.00896) scores showed significant differences between the conditions. Only for PC19, were average scores significantly different between Pre and D1 (p < 0.01974) and between Pre and D3 (p < 0.04058), where distinct differences in pain intensity were predicted.

**Figure 3:**
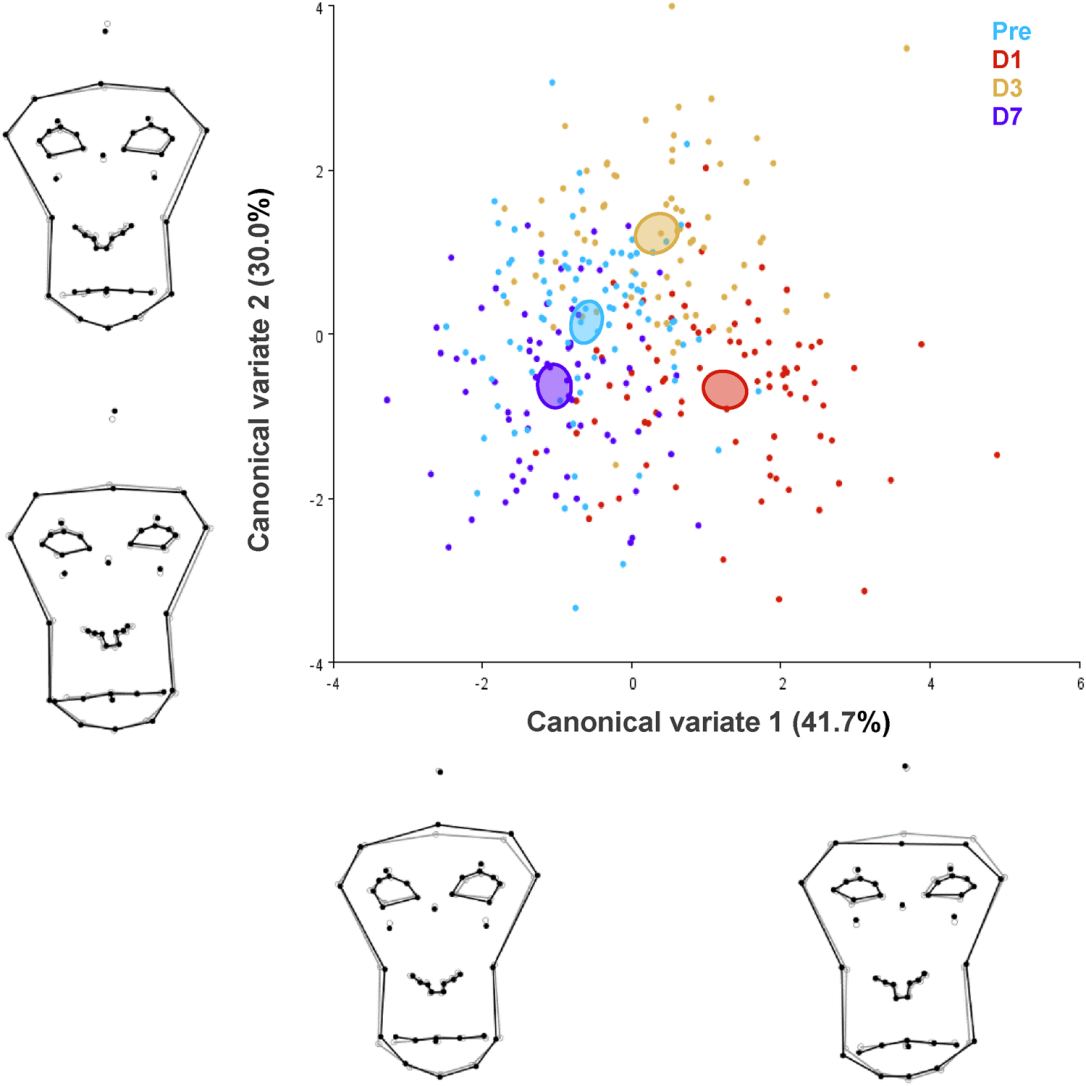
Facial shape variation between conditions after Canonical Variate Analysis. Each colour refers to one condition and confidence ellipses for means are given with probability of 0.9. The wireframes depict changes in shape along the two main canonical variates (black: variation per unit of within-subject variation (Mahalanobis distances) relative to the grey-mean shape. The amount of variation explained by each CV is shown in parentheses.

Differences related to the condition were located using discriminant analysis. Facial shape differences between conditions are given in a CVA scatterplot (see Fig. 3). Along CV1, D1 and D3 presented tightly closed eyelid or eye squeeze compared to Pre and D7. Along CV2, D7 showed less pronounced tightly closed eyelid. Mahalanobis distances among the four conditions are significantly different in all pairwise comparisons (p < 0.05), while Procrustes distances (p < 0.05) diverge (Table 1).

**Table 1:** Differences in the facial shapes between preoperative (Pre) and postoperative condition (D1, D3, D7) in Japanese macaques.

	Pre	D1	D3	D7	D0	D1	D3	D7
Pre	–	< 0.0001	< 0.0001	< 0.0001	–	0.2990	0.0284	0.1999
D1	2.2660	–	< 0.0001	< 0.0001	0.0105	–	0.1488	0.1942
D3	2.1474	2.1950	–	< 0.0001	0.0140	0.0123	–	0.5981
D7	2.0784	2.4469	2.3217	–	0.0111	0.0119	0.0091	–

Mahalanobis distances (left) & Procrustes distances (right): p-values (above); distances between groups (below).

### Individual analysis of variation in facial expression

CVA plots from each of the fourteen subjects were investigated across days. Changes regarding the differences between Pre and D1 were observed by comparing clustered areas on the CVA, which is represented by the wireframes. Pre shape was considered the baseline shape and variation was observed as the configuration changed towards D1. The clustered areas and its associated wireframes indicate that not all characteristics associated with pain are expressed by all individuals (see Supplementary Data S4). The expressions of pain are likely to vary between individuals across a range of facial areas, but tend to occur in the eye and mouth area (Supplementary Table 6).

**Figure 4:**
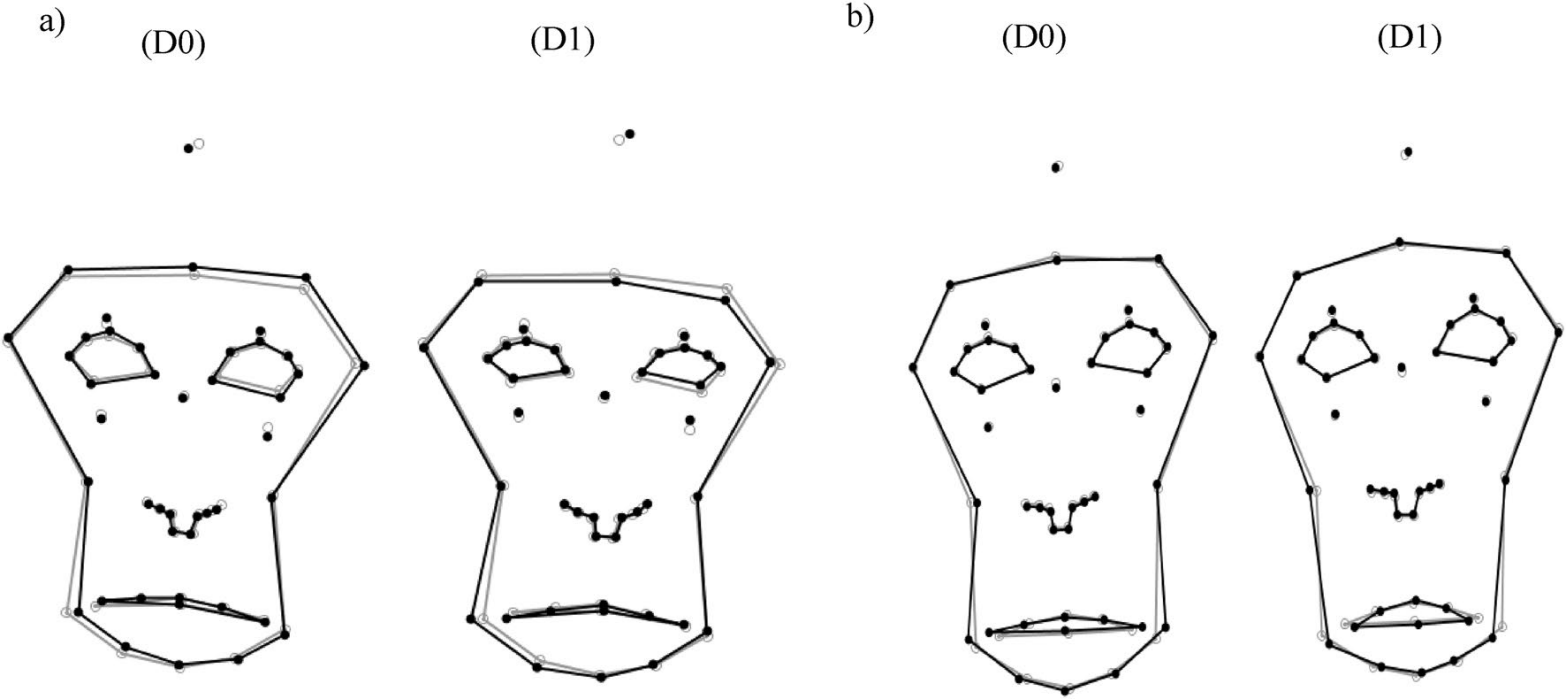
Geometric representation of the face of two representative Japanese macaques. Relative facial shape changes are shown one day after surgery (D1) compared to before surgery (D0; pain-free). Contraction of the eye and tensed lips (**a**) and lip separation (**b**) were observed.

*Eye area* The eyes showed slight or strong asymmetric contraction (Fig. 4). Orbital tightening to various extents were seen in nine subjects.

*Mouth area* Overall, the corners of the mouth (LM3, LM4) were extended toward the lateral areas of the face, in a slight downward change, and with an increased distance from the central point of the lips (LM1). The tension of lips to various extents was seen in ten subjects. Four subjects presented wider separation of the lips (LM1, LM2) compared to the preoperative condition (Fig. 4). In two subjects, the nasal area was shown to move downwards while the mouth line moved upwards, resulting in a smoothed philtral region. The mouth line extension was found to be larger than when compared to the pre-operative shape likely indicating mouth straightening (see Supplementary Data S4). Eight animals showed both orbital tightening and tension of lips.

## Discussion

The aim of the present study was to identify facial changes associated with postoperative pain in Japanese macaques. Expressions associated with pain were observed especially in the mouth and eye area. Animals presented orbital tightening of one or both eyes. All animals presenting unilateral change showed it on the left eye. This could be because emotional processing is believed to be asymmetrical with the right hemisphere dominance for the negative emotional valence, and therefore, greater involvement of the left side of the face in the production of emotional responses is expected^40,41^. Rhesus macaques display more expressive and earlier movements on left side of the face during the production of the fear grimace and the open mouth threat^40^. Such lateralization has also been found in other primates, including humans and chimpanzees^41,42^. Half-closed eyes may be sedation-related or indicate reduced wellness, as observed in rhesus macaques that were potentially unwell prior to their surgical procedure^43^. Accordingly, in our study, animals did not present this feature in Pre as they were considered to be healthy and active before surgery. Orbital changes were not consistent during the whole recording period as facial expressions are dynamic and change according to internal and external environmental factors. Reaction to noise or any perturbation of the surroundings was sufficient to modify the facial expression. When animals are standing or engaging in some activity, the stare is represented by wide open circular eyes. Orbital tightening has also been observed as a sign of pain in mice^17^, rats^44^, rabbits^45^, and sheep^46^. Cats in painful conditions are shown to present a slightly narrowed eye aperture and reduced distance between the cheeks, mouth and nose region”^20,26^. Horses experiencing pain, however, can present squeezing^19^ and widening of both eyes^47^. The eye area in cynomolgus macaques experiencing pain was described as “flinching of the facial muscles around the eyes and/or contraction of the skin at the back-top of the head”^48^. Ogawa et al.^49^ and Yano et al.^48^ have previously used this facial expression as an indicator of whether pain was present or absent during pain threshold tests in osteoarthritis and endometriosis macaque experimental models. This finding provides external convergent validity to our findings.

GMM of group analysis showed subtle differences in facial expressions across days. The shape deformations that explained the difference between painful and pain-free conditions were found in PCs representing a very small part of the total variance and therefore, not easily observable.

We observed that morphological differences between individuals were remarkedly more impactful on the total variation of shape than the difference between groups according to their surgical condition. This was confirmed with the confusion matrix that demonstrates high accuracy in the classification of individuals (92%) compared to a 39% hit rate when assigning images to the condition. Although all females were raised in similar captivity conditions, variation due to genetics, age, body mass and specific facial features impact general facial shape.

Individual analysis showed that the corners of the mouth were extended toward the lateral areas of the face. This indicates that the lips were being pulled, flattened, tensed likely due to changes caused by surgery. Human studies have shown that horizontal stretching and pulling at the corner of the lips were associated with pain^49,50^. Lip tightening was more frequently reported in rhesus macaques before receiving analgesic medication compared to a baseline pain-free condition^43^. Flattened and tightened lips were also observed in lambs experiencing pain as a result of tail-docking and appeared more like a horizontal line during frontal head pictures^51^. Japanese macaques do not have pronounced lips that are easily observed during landmark (LM) annotation. LM1 and LM2 were annotated in the central point of the upper and lower lip, respectively. However, the other landmarks of the mouth (LM3-LM6) were annotated in the apparent junction of the upper and lower lip because the boundaries between the two structures were not clear as observed in other species such as humans and chimpanzees. The separation is visible only when the lips are parted, which was observed occasionally. Therefore, the flattening of the mouth line observed in the wireframes also gave the impression of a more elongated and tenser mouth. Co-occurrence of lip tension and orbital tightening was observed in eight subjects in this study, while co-occurrence of orbital tightening and lips parted was observed in two. In two subjects, the nasal area was shown to move downwards while the mouth line moved upwards, resulting in a smoothed philtral region. The lower lip drawn back caudally, jawline straightening, and shortening and narrowing of the philtrum was also observed in the ‘painful face’ of sheep^46^.

Facial action units used to evaluate pain are species-specific. The evaluation of said action units depends on anatomy, context-specific behaviours, angle of observation, and can be affected by an observer’s anthropocentric bias. In the mouse^17^ and in the ferret^52^, for example, cheek bulge is evaluated, while description of mouth or lip features is absent. The ear action unit has been described in virtually all the species evaluated for the presence of pain, with most of the subjects showing robust changes in painful faces^53^. The ear movements of Japanese macaques have the same musculature basis as rhesus macaques^34^ and play an important role in macaque communication^54^. In the present study, evaluation of ear landmarks was not possible in frontal pictures because, unlike rhesus macaques, the ears of Japanese macaques are covered by hair. Profile pictures may show ears more clearly, but this also depends on the length and density of the individual’s hair. Further investigation of changes in ear movements is required in order to better understand what role the ears may play as a pain cue in these species. Moreover, landmark 16 was annotated on the top of the head with the aim of exploring hair displacement due to piloerection. This phenomenon is rarely described in macaques and seems to be associated with severe viral infections^55,56^. In our study, no remarkable changes were observed in this area.

Captive macaques have been reported to change their behaviour^57^ and mask pain in the presence of an observer and in the period immediately after an observer leaves the room^11^. This phenomenon may be due to several factors such as evolutionary pressure as a prey species, or the risk of losing social rank in the presence of conspecifics, and/or habituation to researchers and caretakers. On the contrary, other prey species such as horses do not seem to suppress changes after a painful stimulus in the presence of an observer and, in addition, increase contact-seeking behaviour^47^. This might reflect differences in the human-animal relationship, associated with domestication or more proximate developmental factors (life-time experience—ontogeny). In the present study, an observer was absent in order to avoid interference with the macaques’ behaviour and enable the recording of spontaneous facial expressions. Idiosyncrasies and personality traits could also contribute to the degree of pain expressed by each individual.

Substantial inter-individual differences are observed in response to internal and external physical and social environments. Pain expression has been reported to be linked to personality in horses^58^ and humans^59,60^. Studies looking at thermal stimuli in horses showed that some animals responded with a skin twitch, although others did not and that this was not necessarily related to their breed^61^. In cats, pain scoring is shown to be affected by demeanour. Shy and fearful individuals may present with high scores of pain which might confound findings and potentially result in inappropriate administration of analgesia^62^. However it should also be acknowledged that an increase in the expression of emotions like fear can arise when an animal is in pain, as it builds fearful associations in anticipation of nociceptive responses or in uncertain environments^63^. In this study, while different changes in facial configuration can be associated with pain, we do not expect them to occur in all subjects at any given time or necessarily over identical timeframes in relation to the same tissue damage. Indeed it has been observed in both humans^64^ and rhesus macaques^43^ that not all individuals show all facial changes associated with pain.

Analgesia treatment may also have contributed to some of the subtle expressions observed. It is likely that residual effects of buprenorphine and carprofen favoured this finding. For ethical reasons and due to the intense and persistent pain of laparotomy, analgesia was not withheld for the purpose of this study. Response to deliberately evoked non-clinical pain stimuli might provide stronger evidence of a “pain face” as documented in mice^17^, rats^44^ and horses^47^. It is also possible that the less acute nature of postoperative pain^65^, and residual effects of anaesthetics resulted in less obvious pain expression over time. However, assessment and description of pain facial features were successfully achieved in animals undergoing surgeries such as castration and ovariohysterectomy^19,26^.

Although an evident homology of facial musculature and similar facial expressions are found between humans and non-human primates, caution should be used in their interpretation with respect to their meaning. The description of facial signals of pain should be species-specific due their individual anatomic features and social context. In addition, experience with the species is shown to be associated with better judgment of emotional states^66^. Recently, automated recognition of pain has been shown to be accurate in horses, both discriminating the presence (88.3%) and level of pain (75.8%)^67^. Automated pain recognition was also successful in cats^68^. Computer vision and machine learning techniques hold promise for unveiling animal emotions with considerably more detail hidden from the human eye, less risk of anthropocentric biases, and the potential to outperform human evaluation^44,67,69^. There are still hurdles to overcome before this technique can be broadly applied, noting that a large number of observations are necessary and depending on the form of learning used, individual labelling may need to be performed by humans to train the new system.

We acknowledge several limitations to this study, but these do not undermine our main conclusions. First, image standardization was challenging. Subjects were awake, conscious and unrestrained within their housing area in order to minimize the disruption of naturally occurring facial expressions. Previous studies have also reported subjects moving in their housing area as well as angle and light changes making it difficult to assess some facial regions^51^. Second, correcting for the effect of orientation may result in loss of biological information not geometrically correlated with head rotation. Third, we assumed that changes in facial expression after surgery were associated with pain, however there is a possibility that this is not the case. The complexity of facial expressions does not exclude other emotional states such as fear or anxiety, although this was beyond the scope of the present study and future studies may be able to investigate such differences. And fourth, the development and validation of structured indices of pain for macaques are required for benchmarking the present findings.

In conclusion, geometric morphometrics was a useful tool for evaluating subtle changes in the facial expressions of Japanese macaques, with excellent overall inter-rater reliability. Facial changes associated with pain include orbital tightening, asymmetrical aperture of the eyes, tension of the lips and elongated mouth line. These findings highlight how pain is not expressed in a single or uniform facial activity pattern and cues are subject- and intensity-dependent. GMM is a potentially useful addition to behaviour-based pain evaluation, which will improve the recognition of pain and ultimately the welfare of macaques in captivity.

## Supplementary Information

Dataset S1.

Dataset S2.

Dataset S3.

Dataset S4.

## Data Availability

The datasets generated and analysed during this study are included in this published article (and its Supplementary Files). Data used in PCA and CVA using MorphoJ: full dataset (n = 1074) and sub population (n = 308) are available as Supplementary Data S2. Data used for ANOVAs are available as Supplementary Data S3. Further inquiries can be directed to the corresponding author.
