# Author Correction: Investigating subtle changes in facial expression to assess acute pain in Japanese macaques

**DOI:** 10.1038/s41598-023-40053-4

**Published:** 2023-08-09

**Authors:** Vanessa N. Gris, Nelson Broche, Akihisa Kaneko, Munehiro Okamoto, Juri Suzuki, Daniel S. Mills, Takako Miyabe-Nishiwaki

**Affiliations:** 1 Primate Research Institute, Kyoto University, Inuyama, Japan; 2 Center for the Evolutionary Origins of Human Behavior, Kyoto University, 41-2 Kanrin, Inuyama, Aichi 484-8506 Japan; 3 Department of Life Sciences, University of Lincoln, Lincoln, UK

Correction to: *Scientific Reports* https://doi.org/10.1038/s41598-022-23595-x, published online 16 November 2022

The original version of this Article contained an error in Supplementary Information file 4, where the wireframe images of Figure S2 in Dataset 4 were incorrectly located.

The Supplementary Information file 4 that accompanies the original Article has been corrected.

### Supplementary Information

Dataset S4.

