# Supplementary material for: Author Correction: Investigating subtle changes in facial expression to assess acute pain in Japanese macaques
Source: Sci Rep. 2023 Aug 9;13:12931. doi: 10.1038/s41598-023-40053-4 (PMC10412631; doi:10.1038/s41598-023-40053-4)
Supplement: Supplementary file 1 — Dataset S4. [file 41598_2023_40053_MOESM1_ESM.docx]

**Supplementary Information**

Investigating subtle changes in facial expression to assess acute pain in Japanese macaques

Vanessa N Gris^1,2^, Nelson Broche Jr.^1,2^, Akihisa Kaneko^1,2^, Munehiro Okamoto^1,2^, Juri Suzuki^1^, Daniel S Mills^3^, Takako Miyabe-Nishiwaki^1,2*^

^1^ Primate Research Institute, Kyoto University, Inuyama, Japan

^2^ Center for the Evolutionary Origins of Human Behavior, Kyoto University, Inuyama, Japan

^3^ School of Life Sciences, University of Lincoln, Lincoln, United Kingdom

Corresponding author: *miyabe.takako.2s@kyoto-u.ac.jp*

**Supplementary Figure 1. Principal Component Analysis of facial shape in Japanese macaques.** Group shape variation in four conditions. (a) PC1 and PC2 overall explain 36.4% of the total variance. (b) PC13 (1.6%) and PC19 (0.95%) showed separation between conditions. The wireframes depict changes in shape along the two main axes of variation (black: the maximum and minimum values on the axis [Mahalanobis distances] grey - mean shape). The confidence ellipses for means are given with a probability of 0.9.

(b)


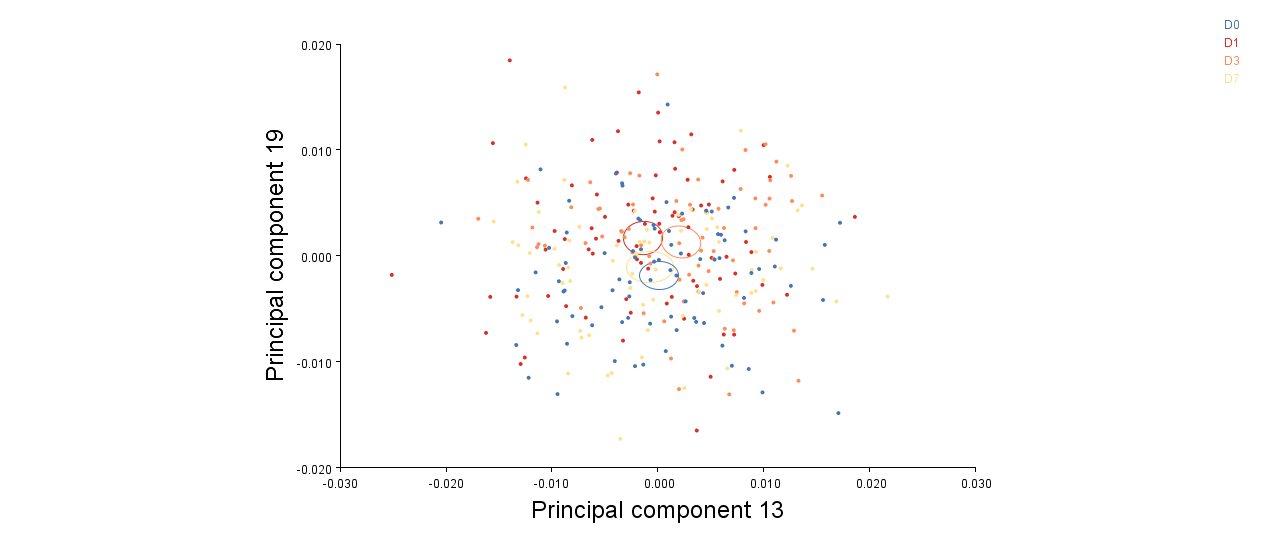

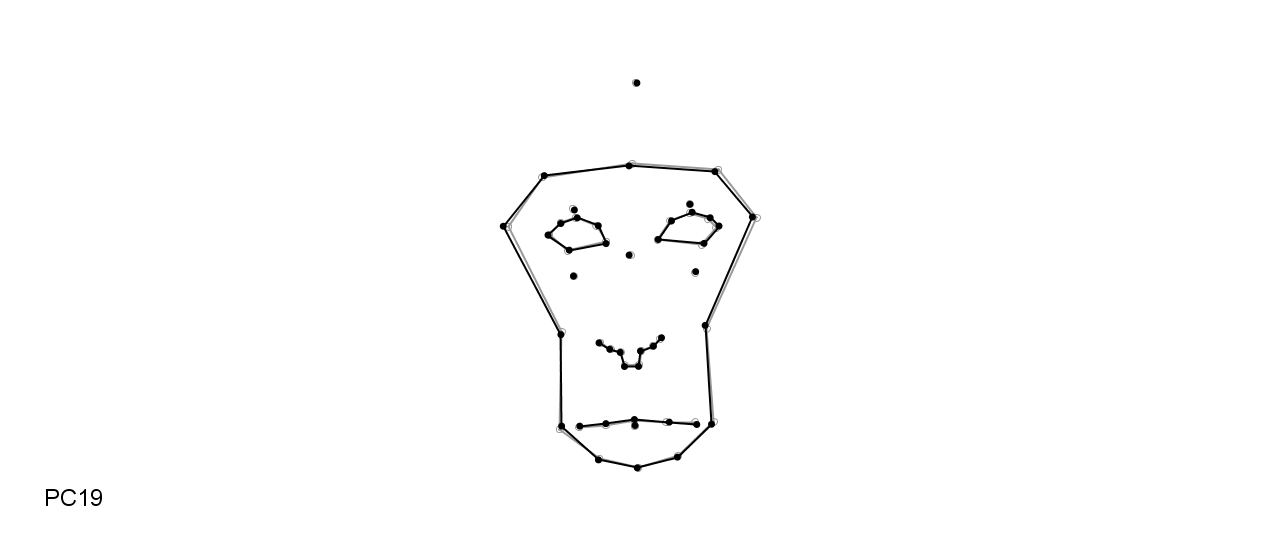

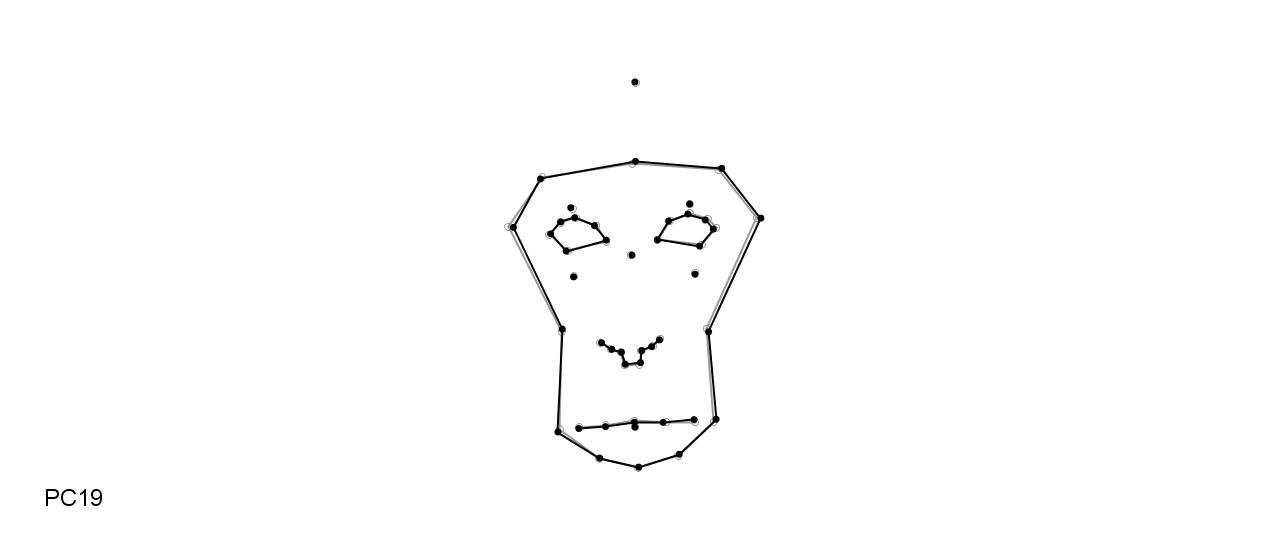

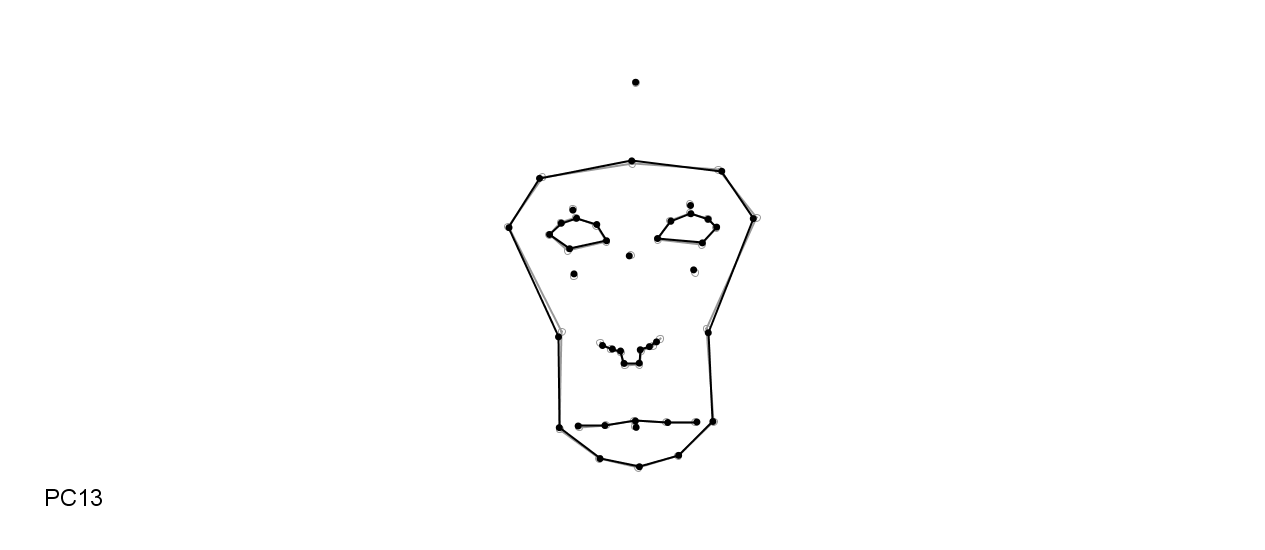

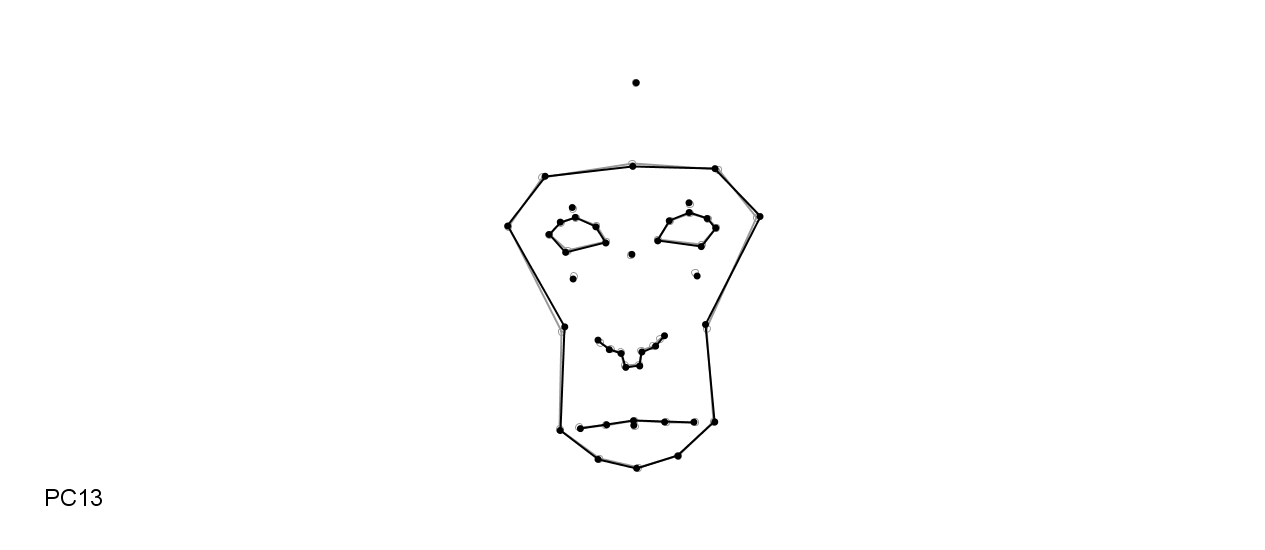


Pre

D1

D3

D7


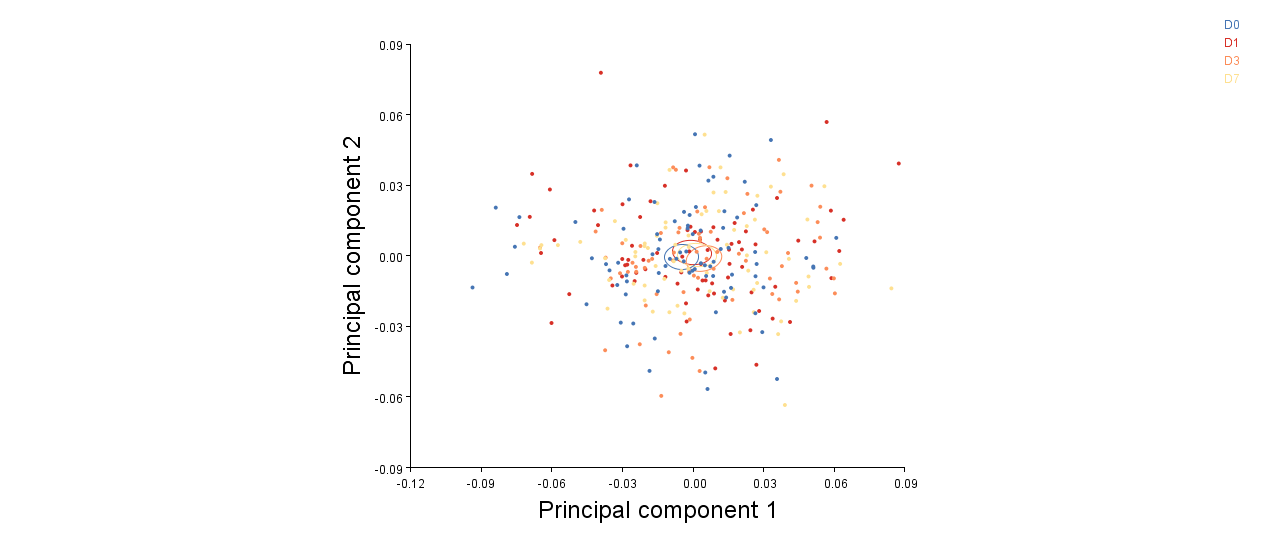


(a)


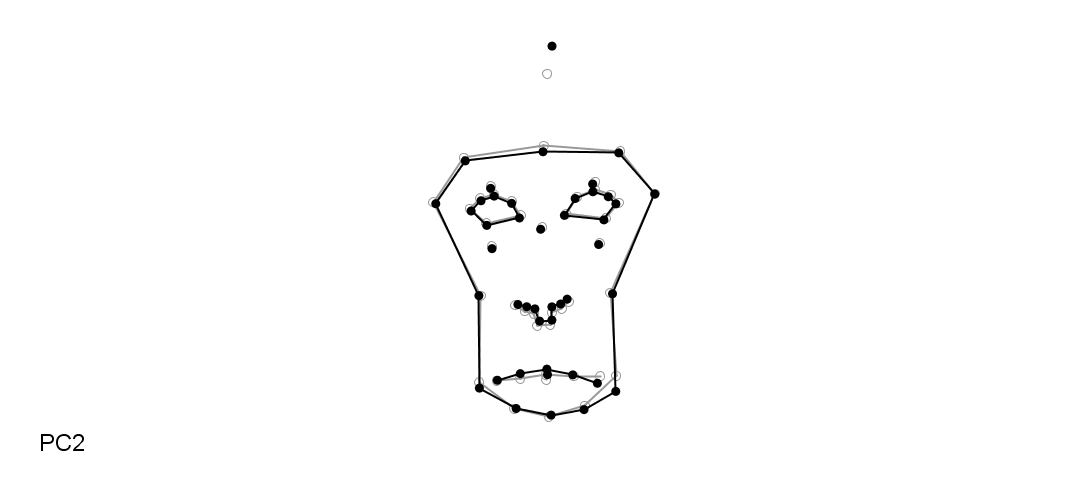

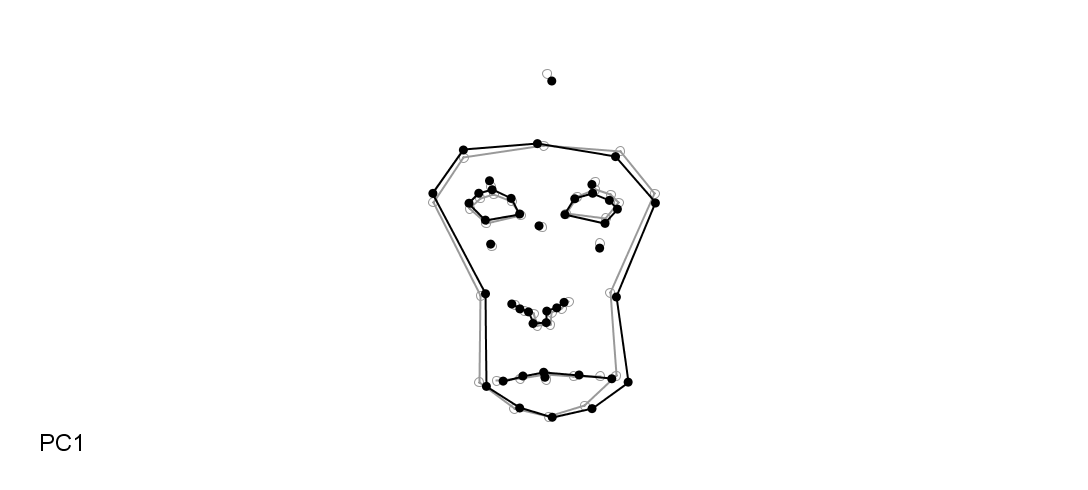

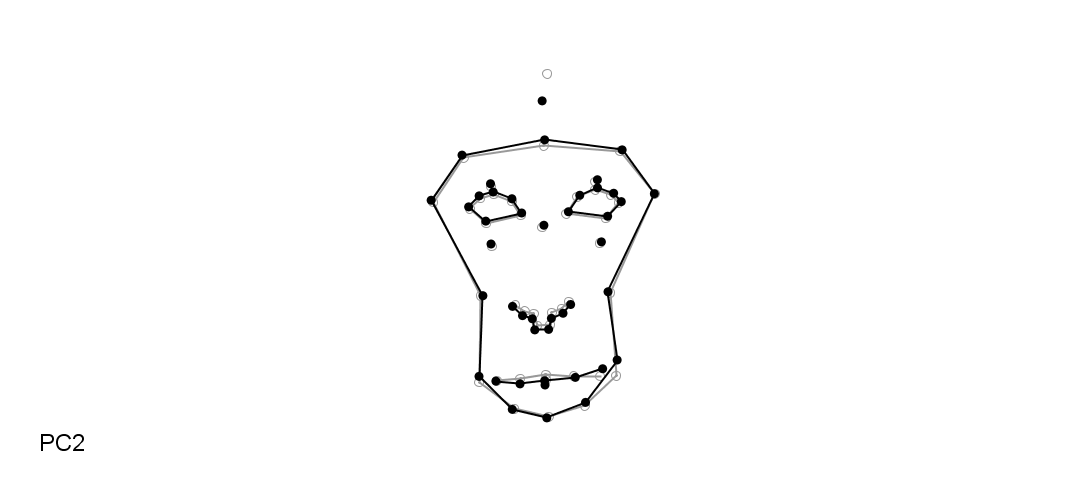

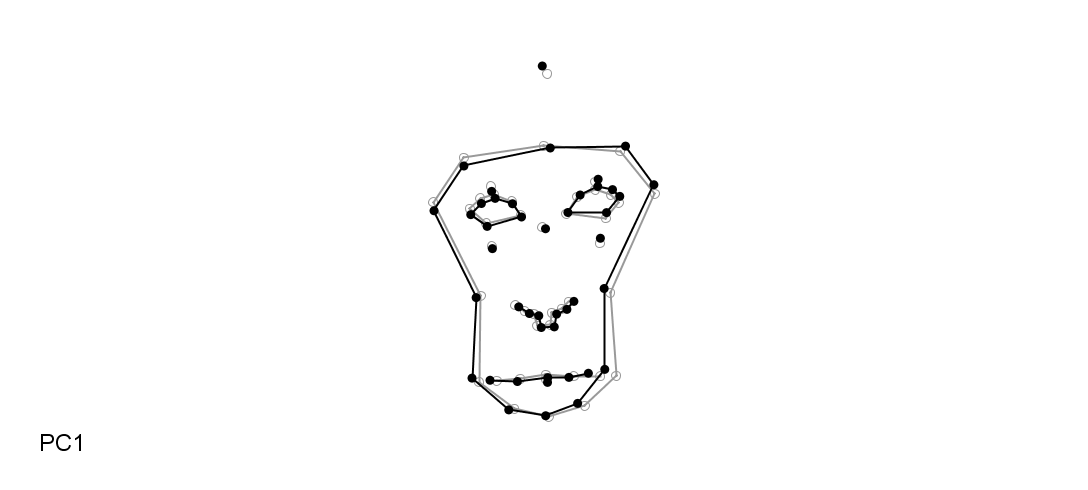


Pre

D1

D3

D7

**Supplementary Figure 2.** **Canonical Variate Analysis showing facial shape variation among female Japanese macaques.** Each colour refers to one subject across the four conditions with 90% confidence ellipses of population means. The wireframes depict changes in shape along the two main canonical variates (black: variation per unit of within-subject variation (Mahalanobis distances: [- 6 to + 9]) relative to the grey - mean shape). The amount of variation explained by each CV is shown in brackets.


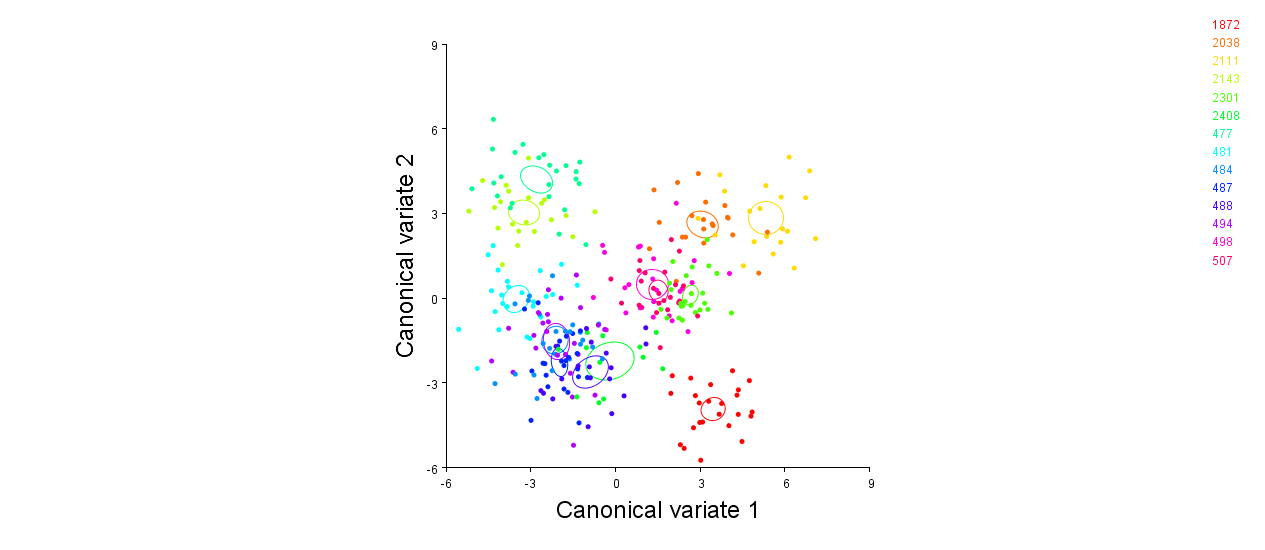

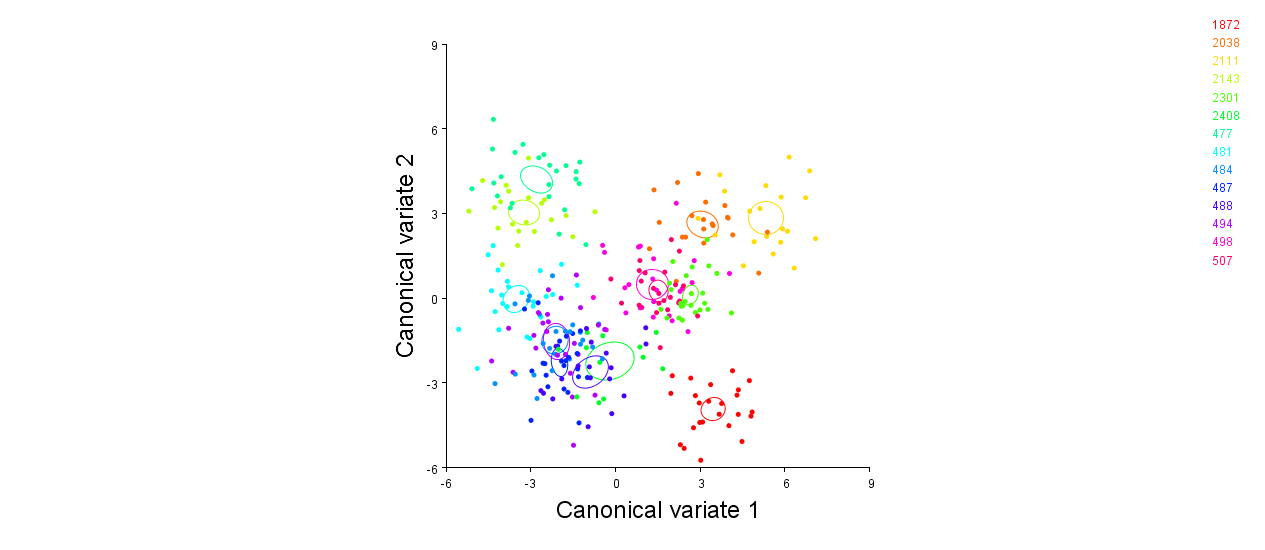


**Canonical variate 1 (20.5%)**

**Canonical variate 2 (15.0%)**


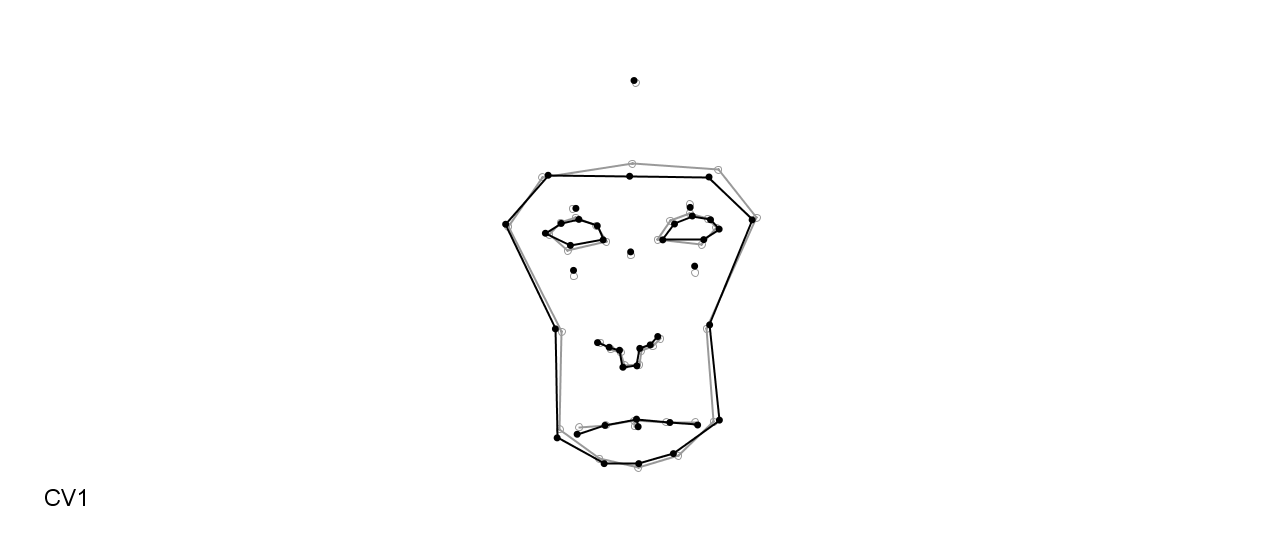

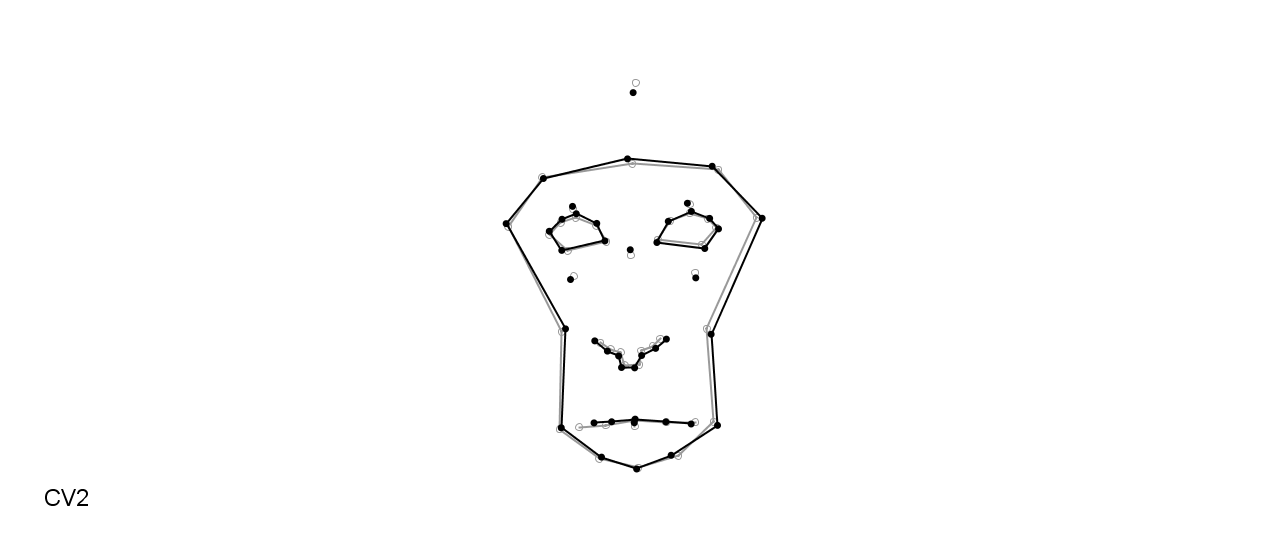

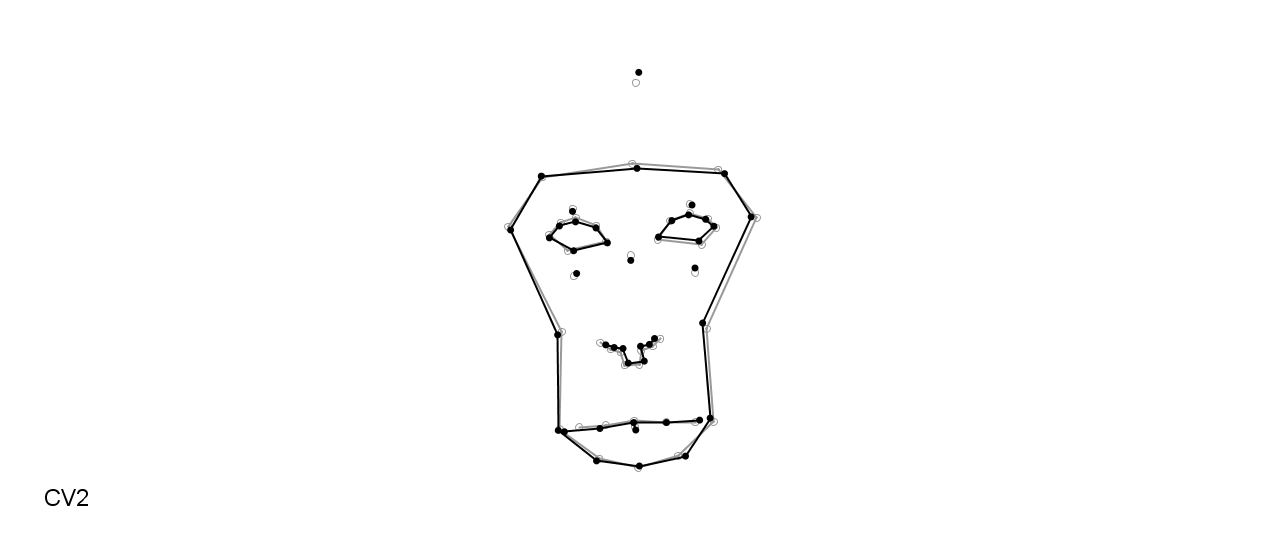

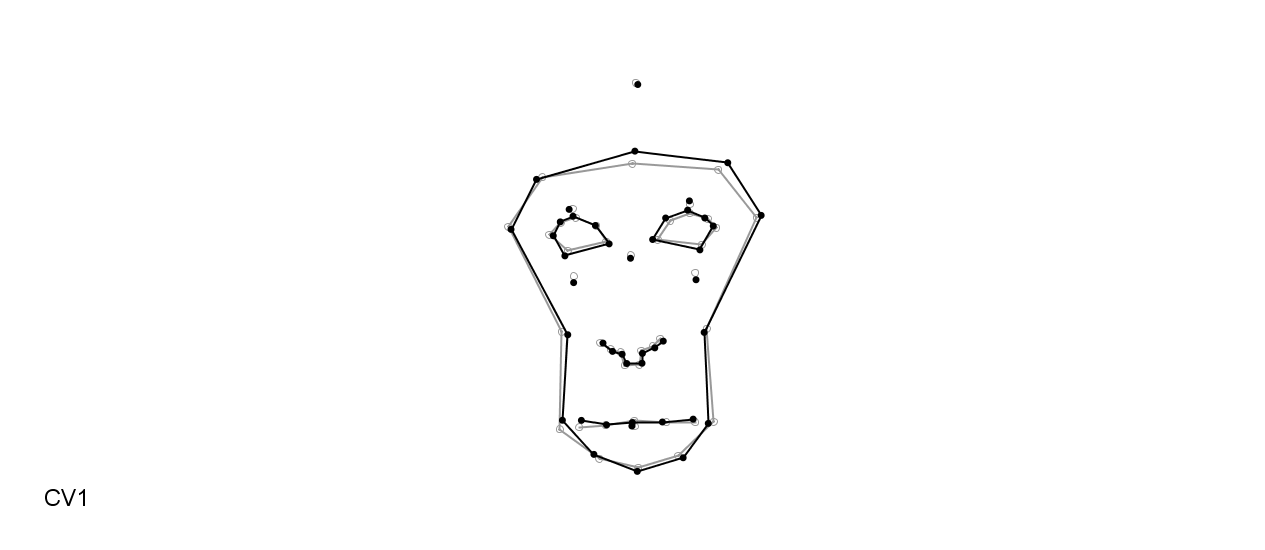


**Supplementary Figure 3. Canonical Variate Analysis of the Procrustes of fourteen Japanese macaques across preoperative (Pre) and postoperative (D1, D3, D7) conditions.**  The wireframes depict changes in shape along the two main axes of variation (black: the maximum and minimum values on the axis [Mahalanobis distances] grey - mean shape for each axis). The confidence ellipses for means are given with a probability of 0.9.


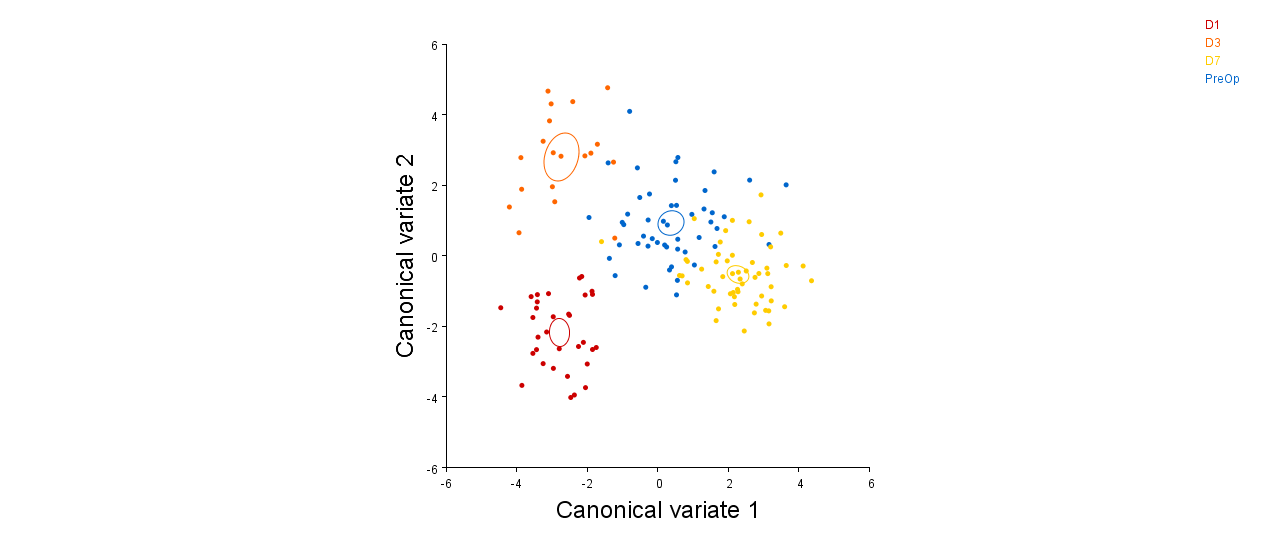

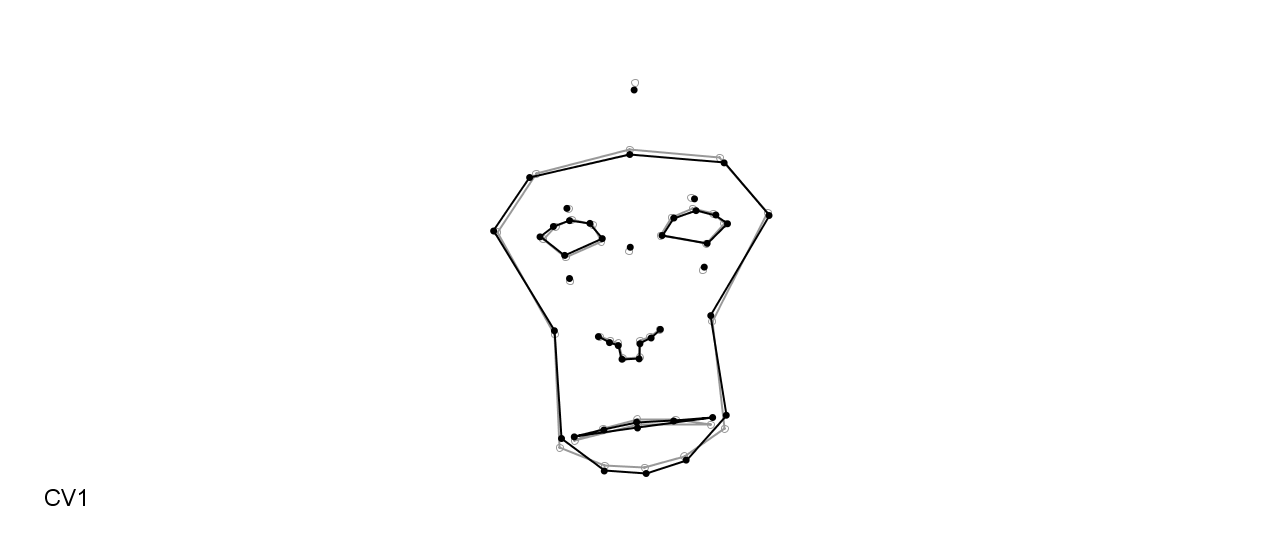

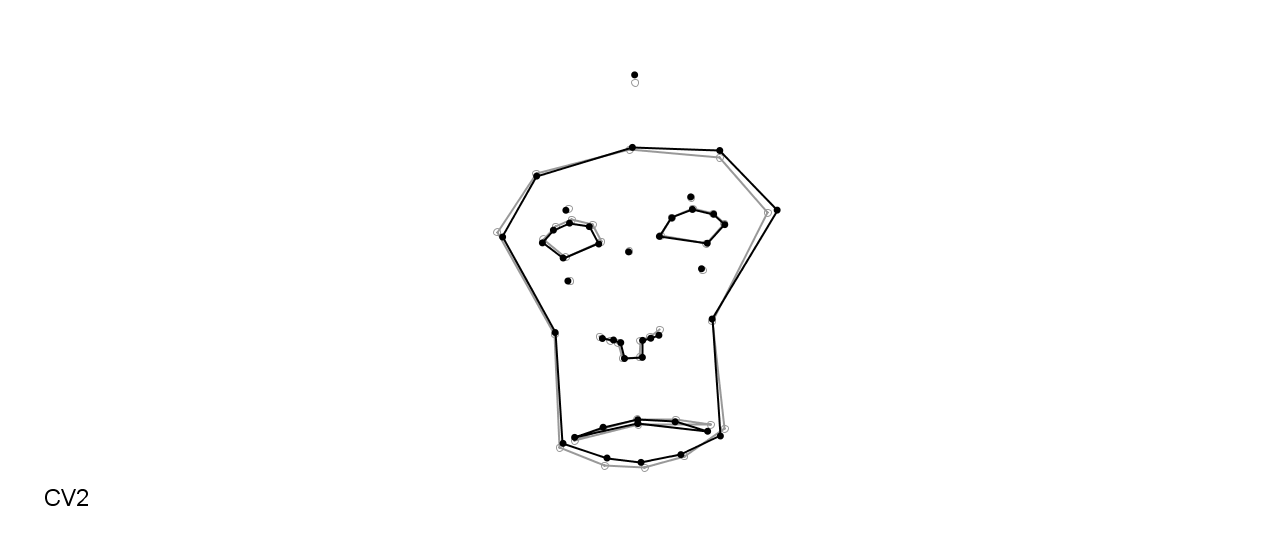

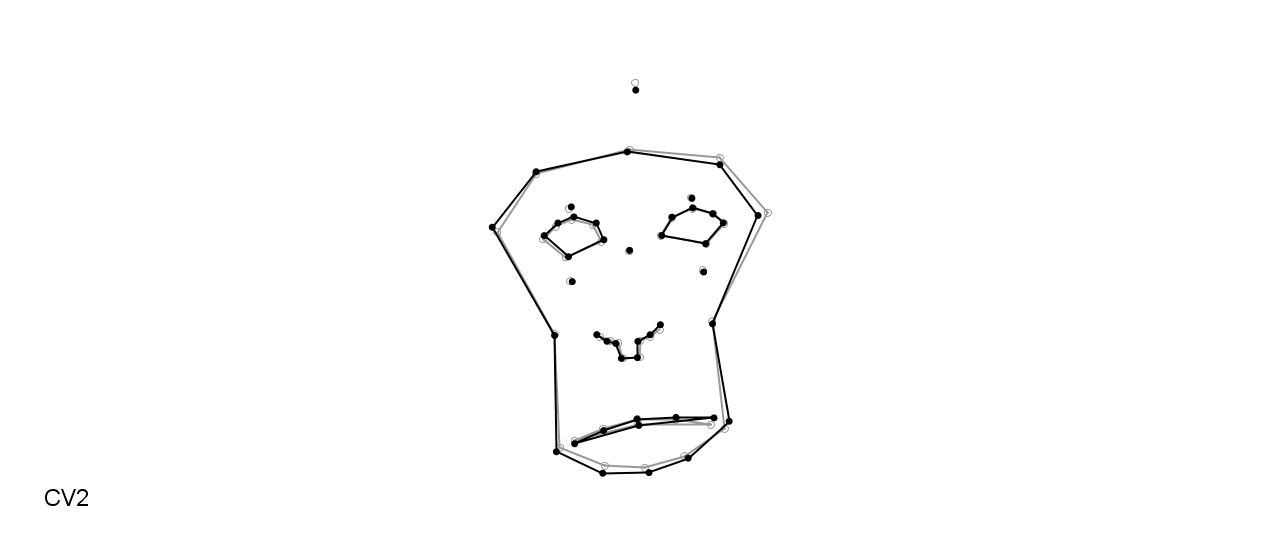

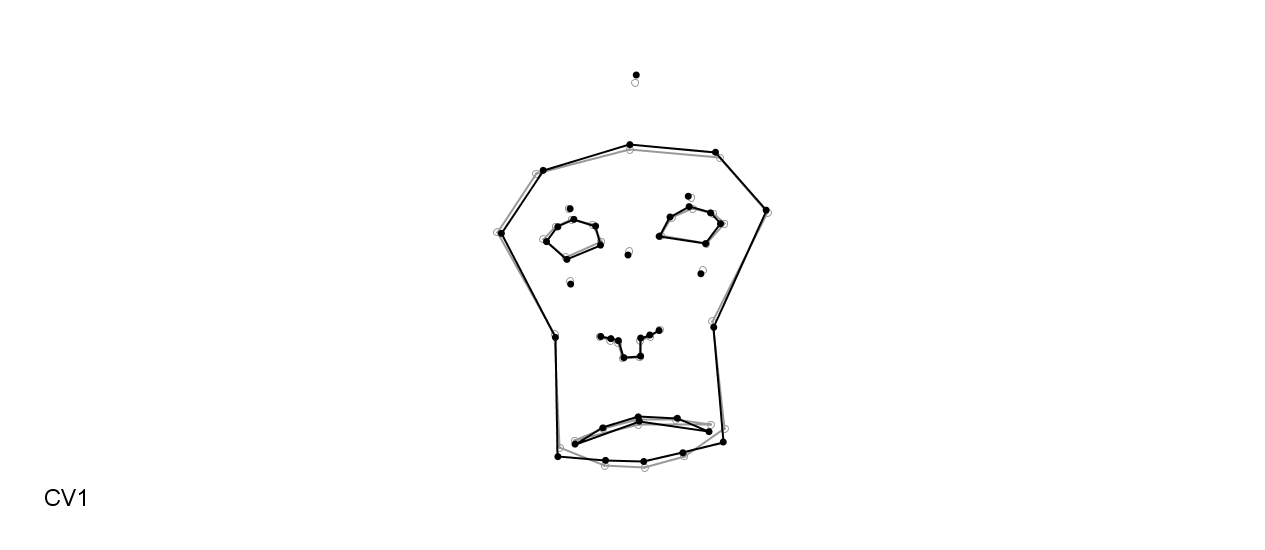


Pre

D1

D3

D7


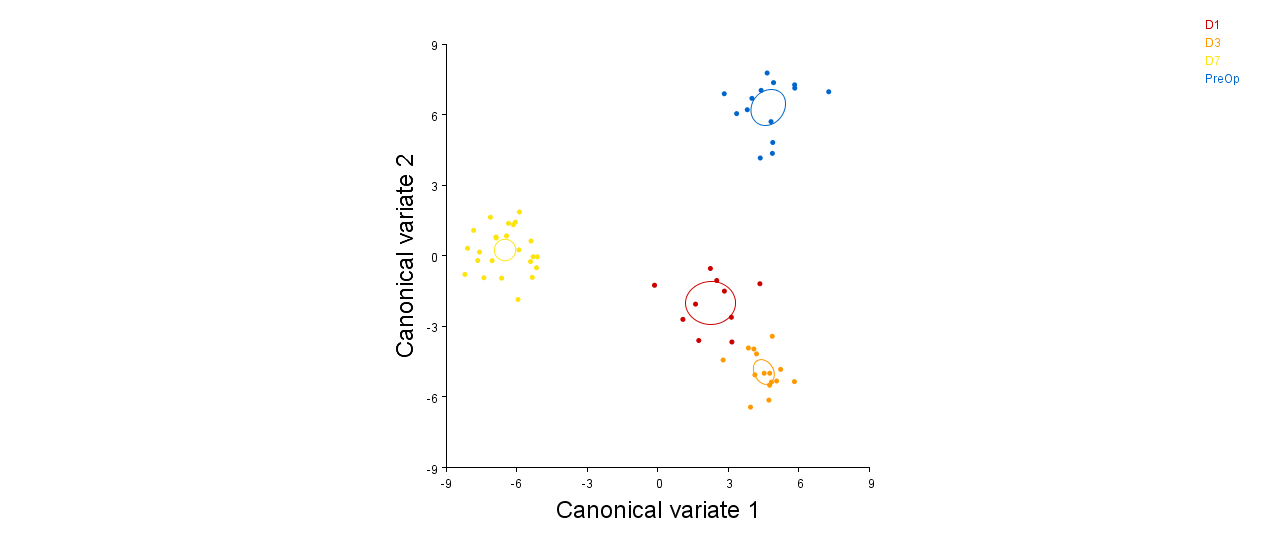

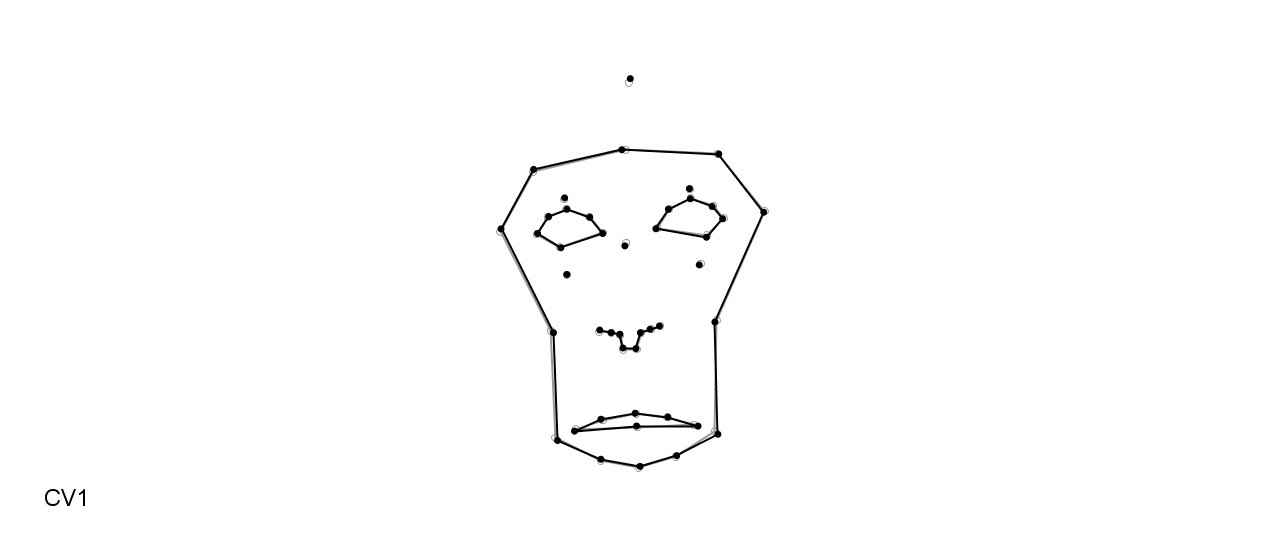

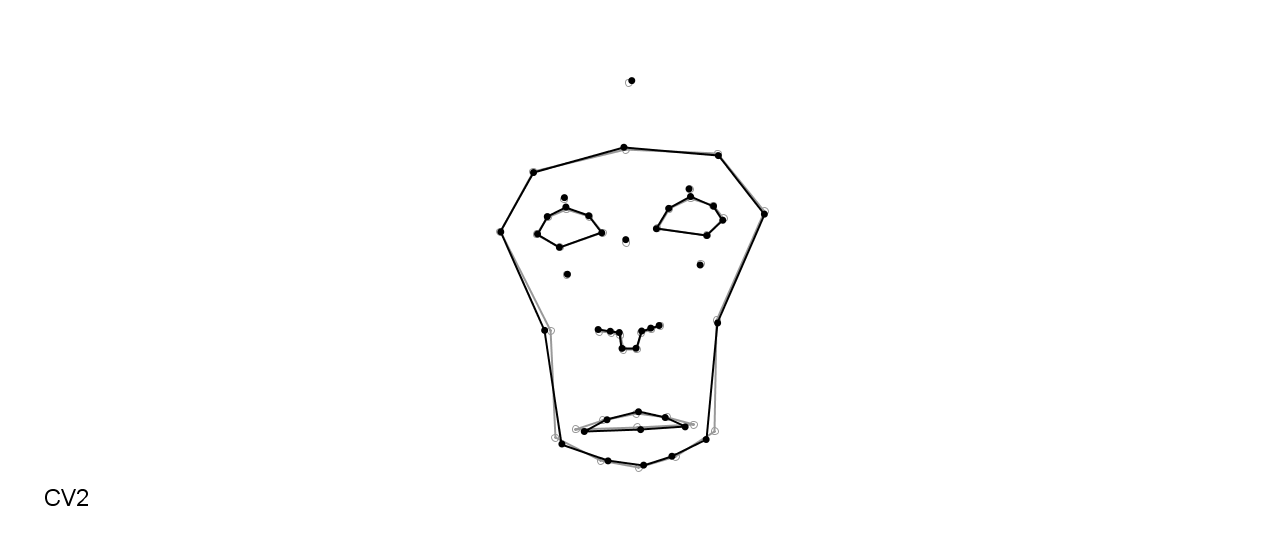

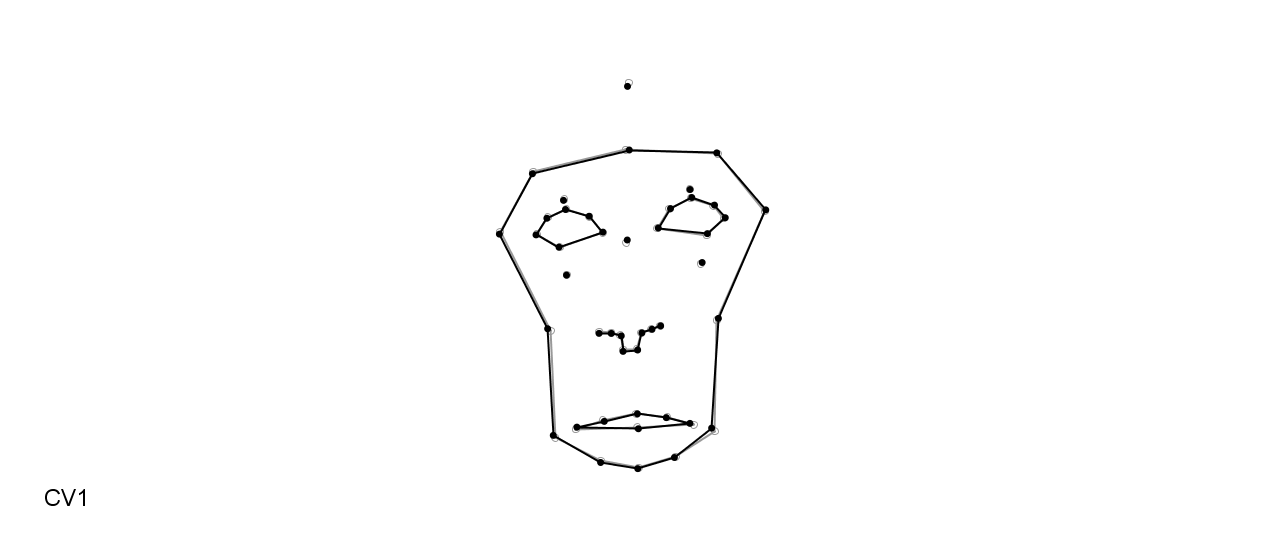

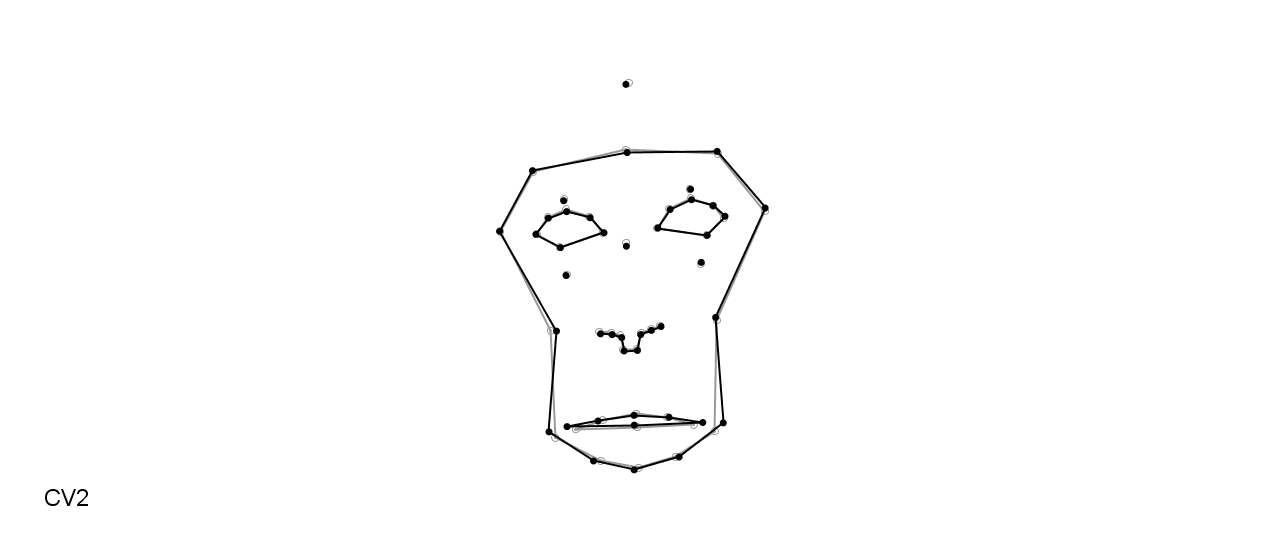


Pre

D1

D3

D7


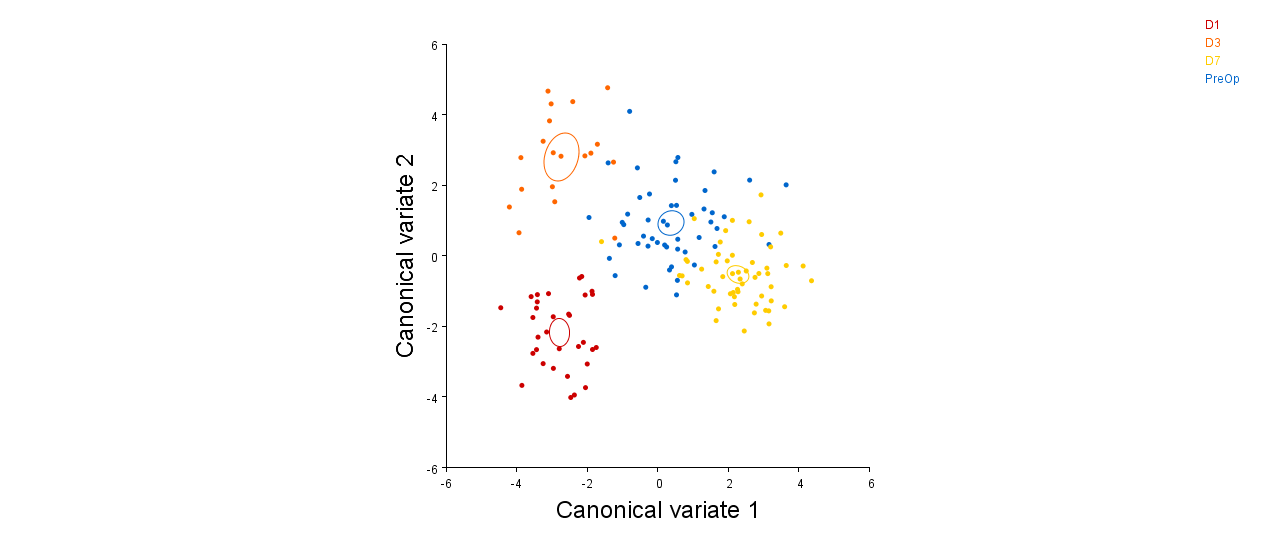

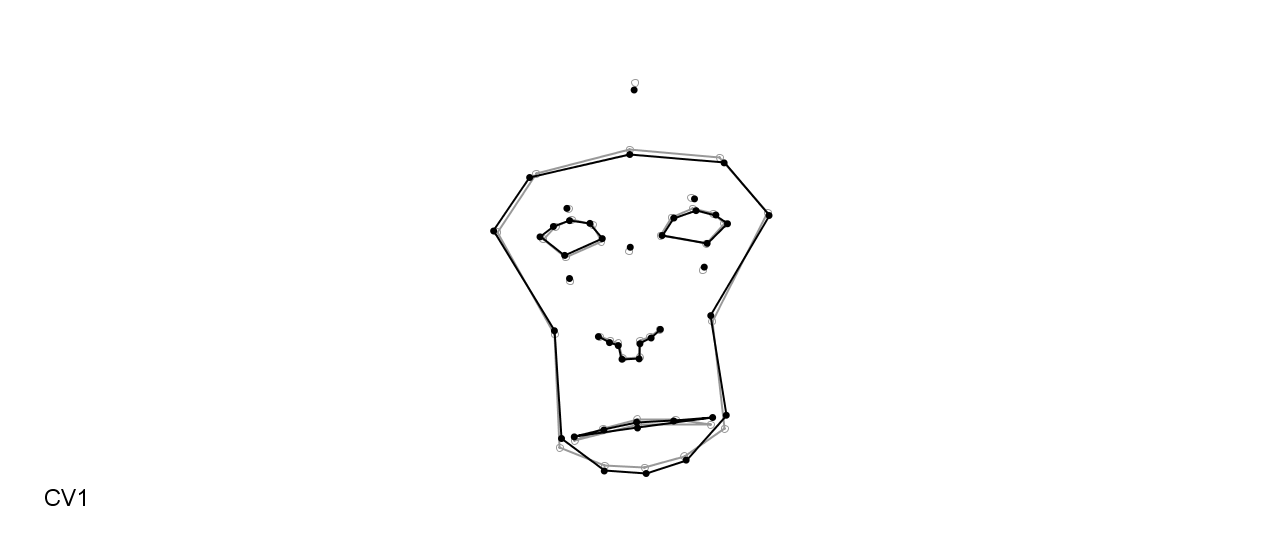

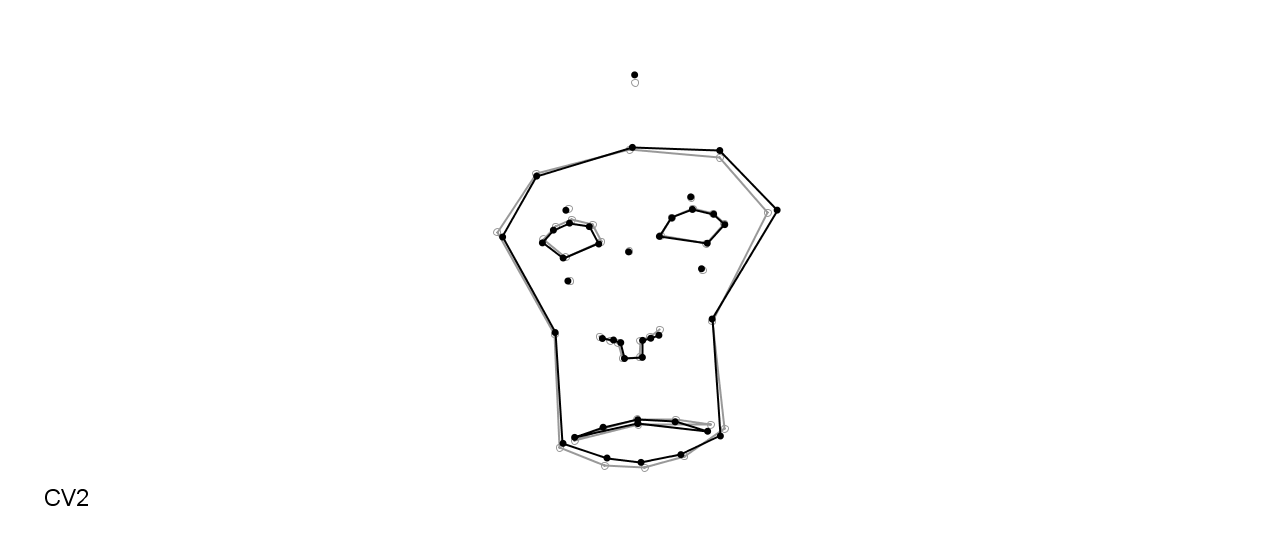

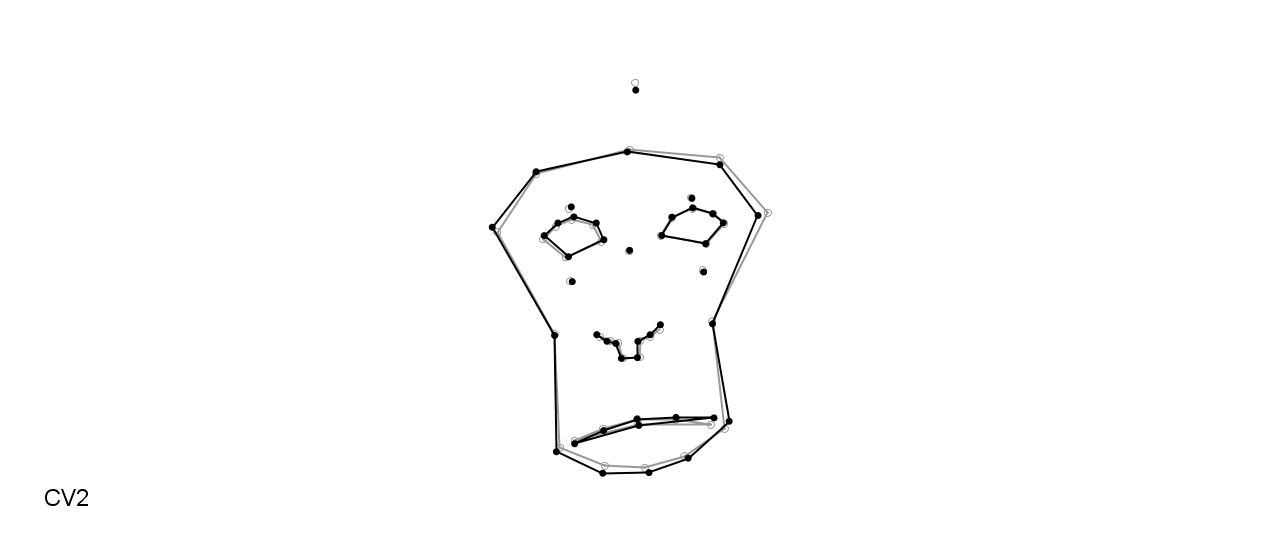

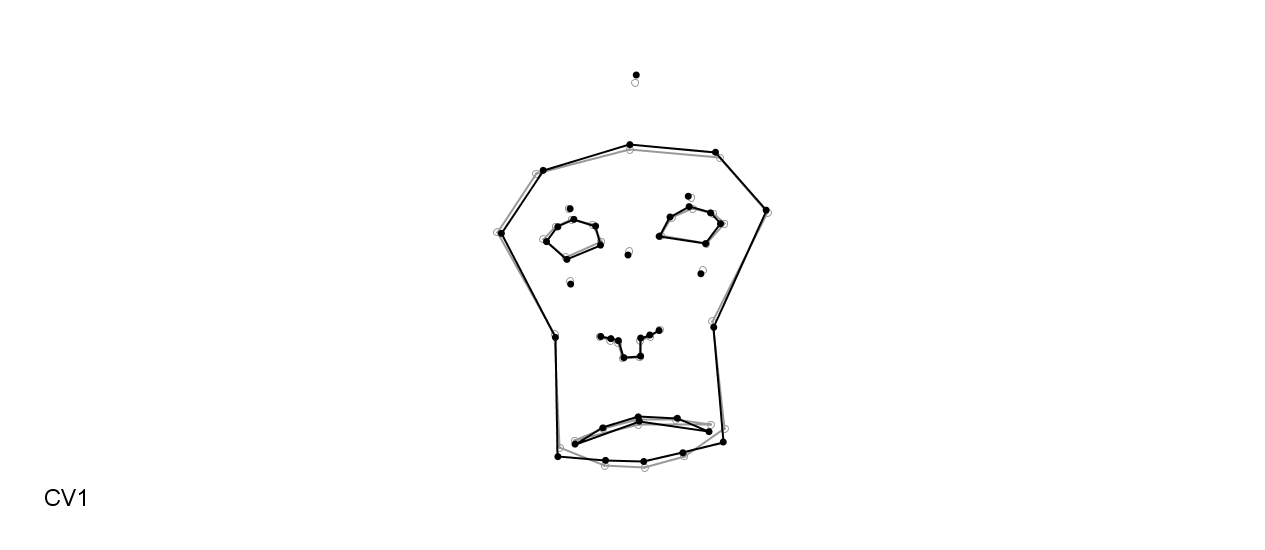

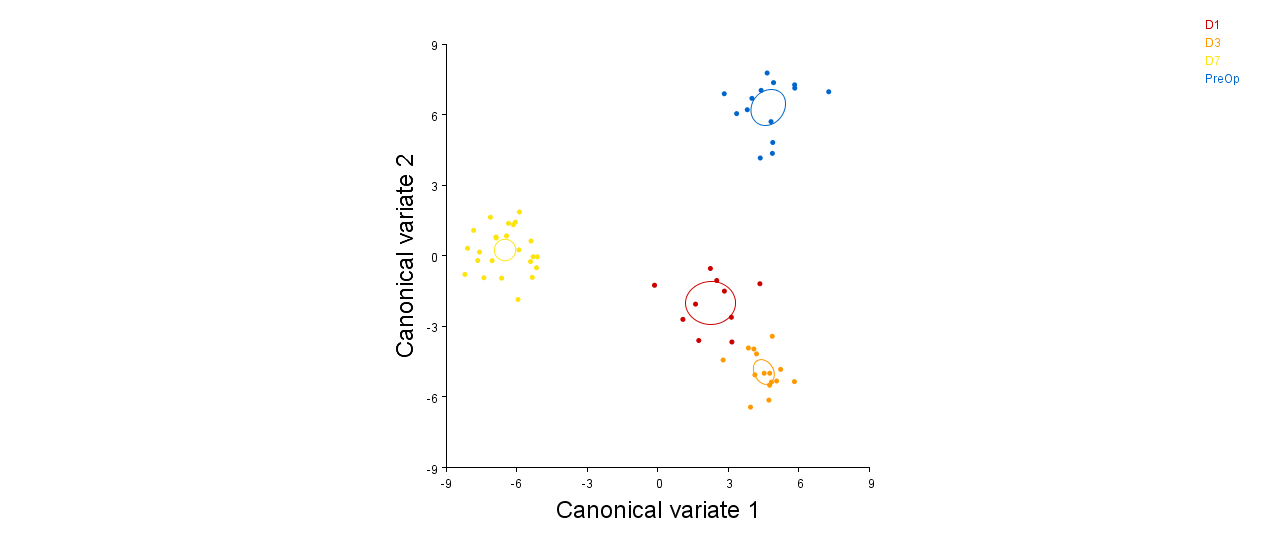

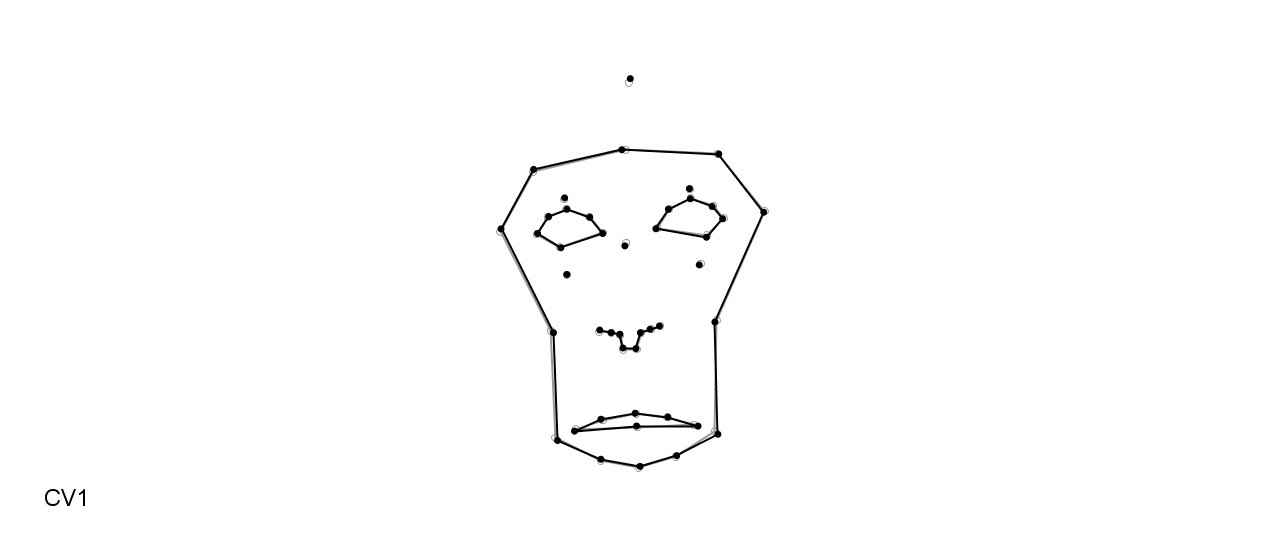

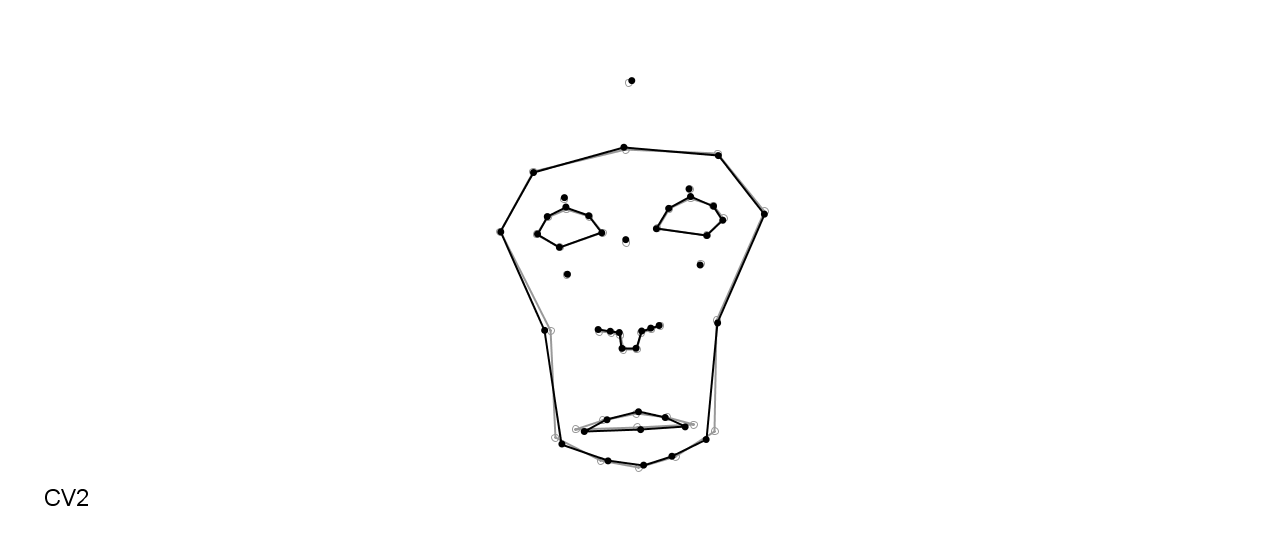

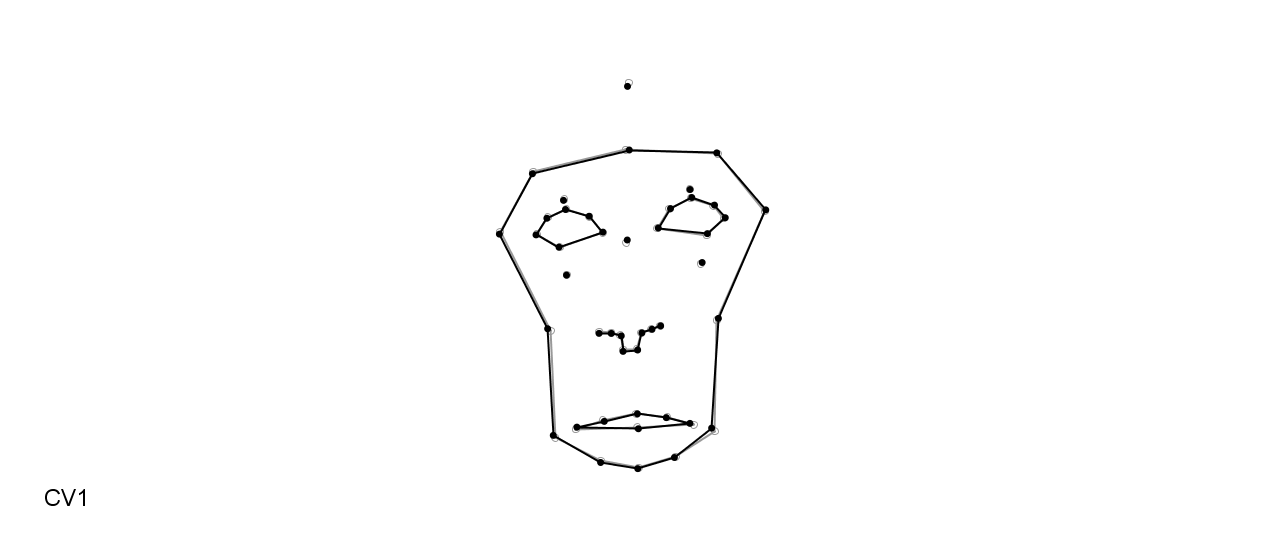

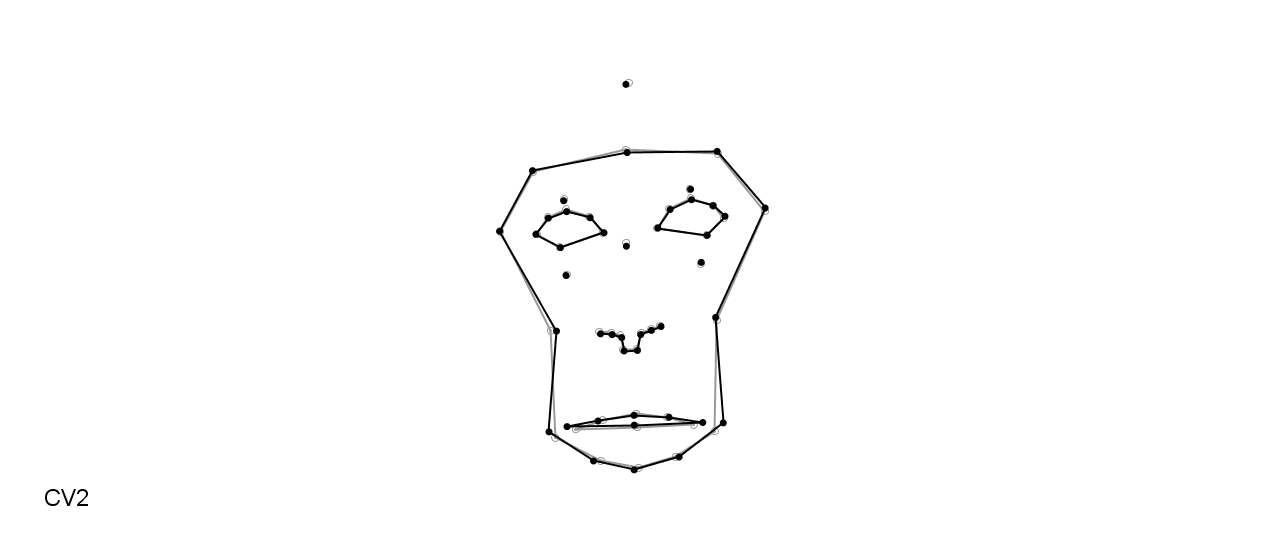


(4)


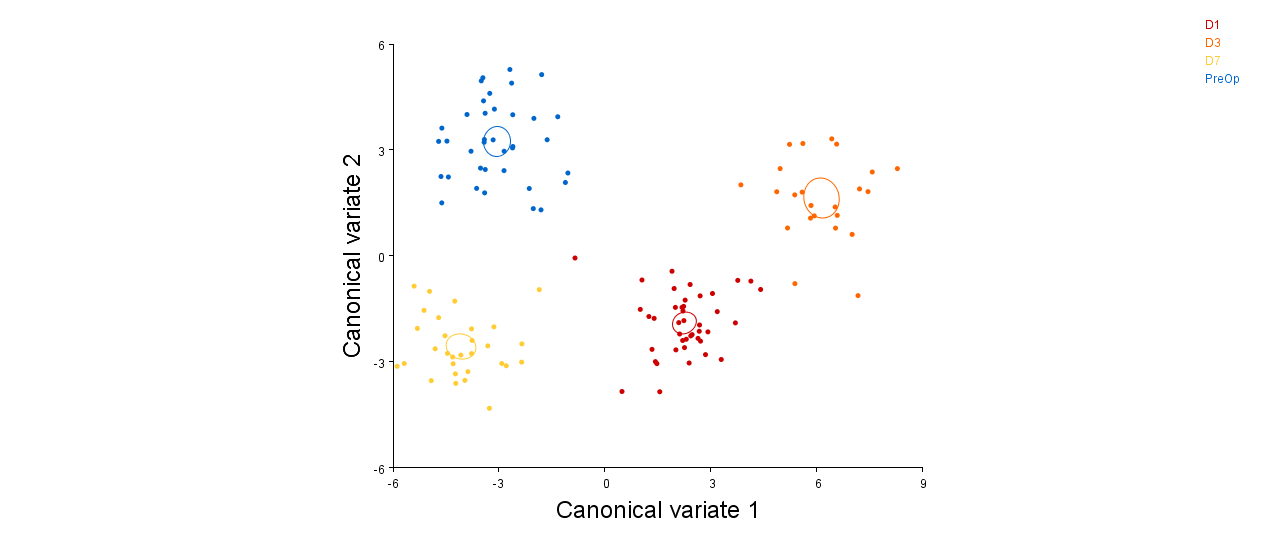

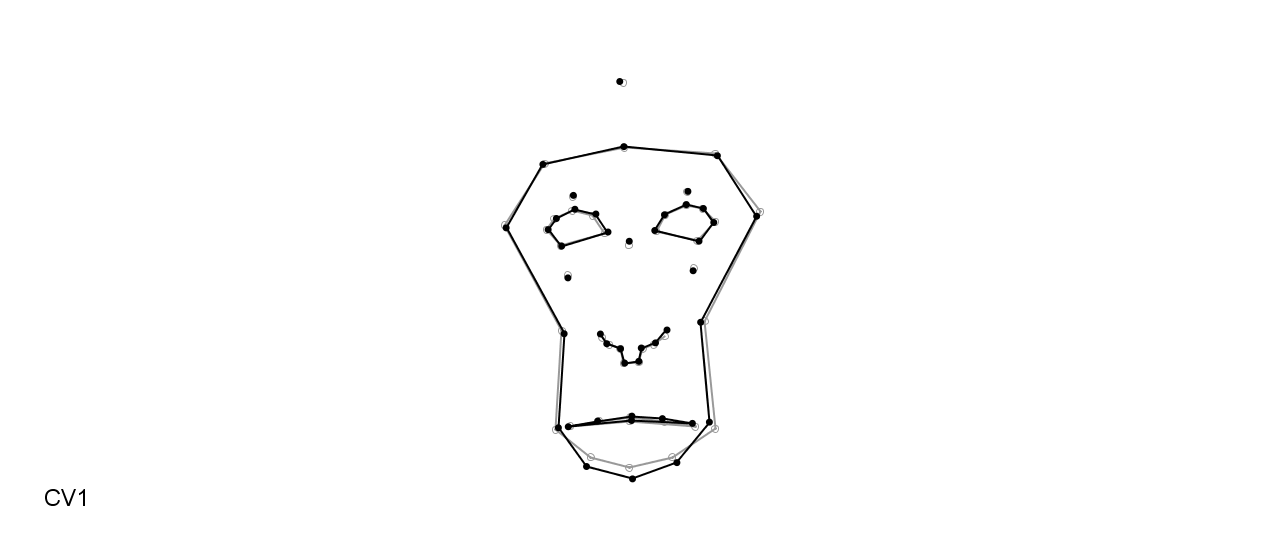

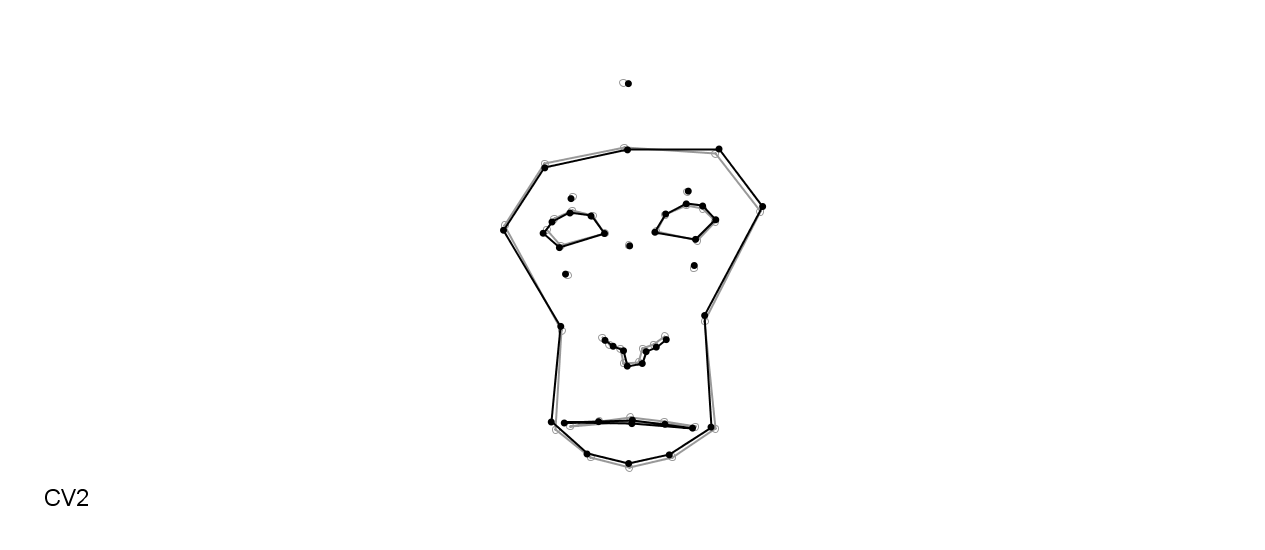

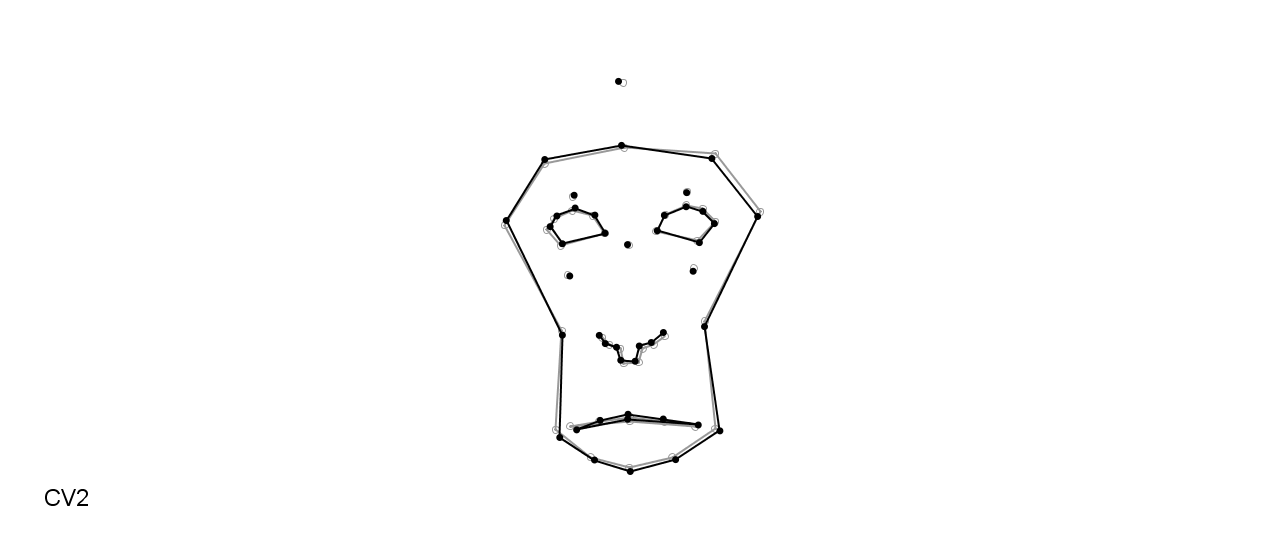

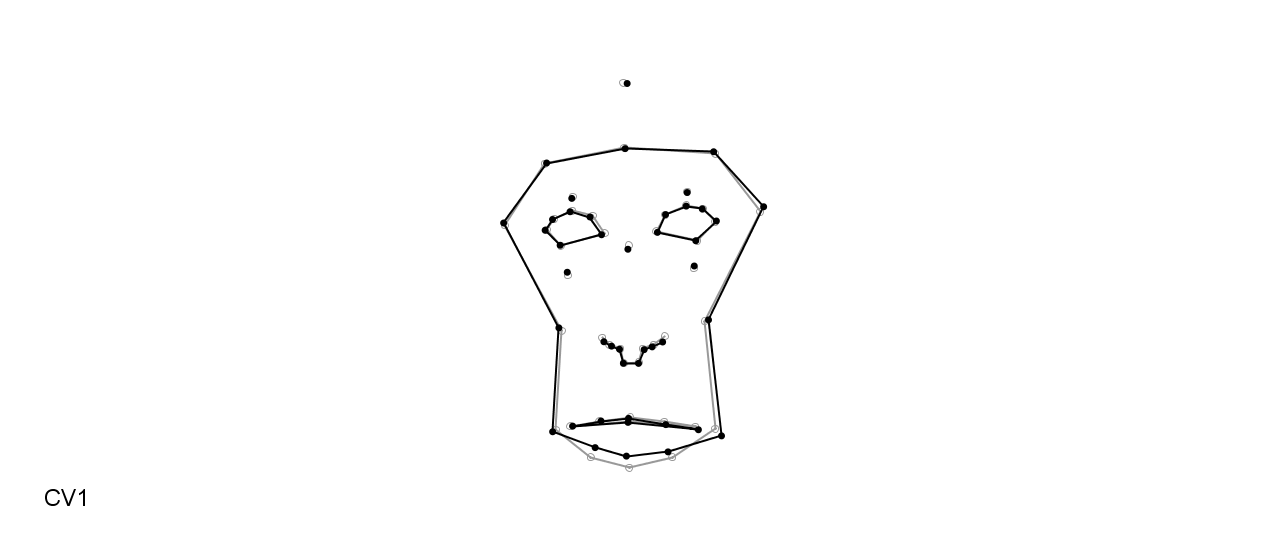


Pre

D1

D3

D7


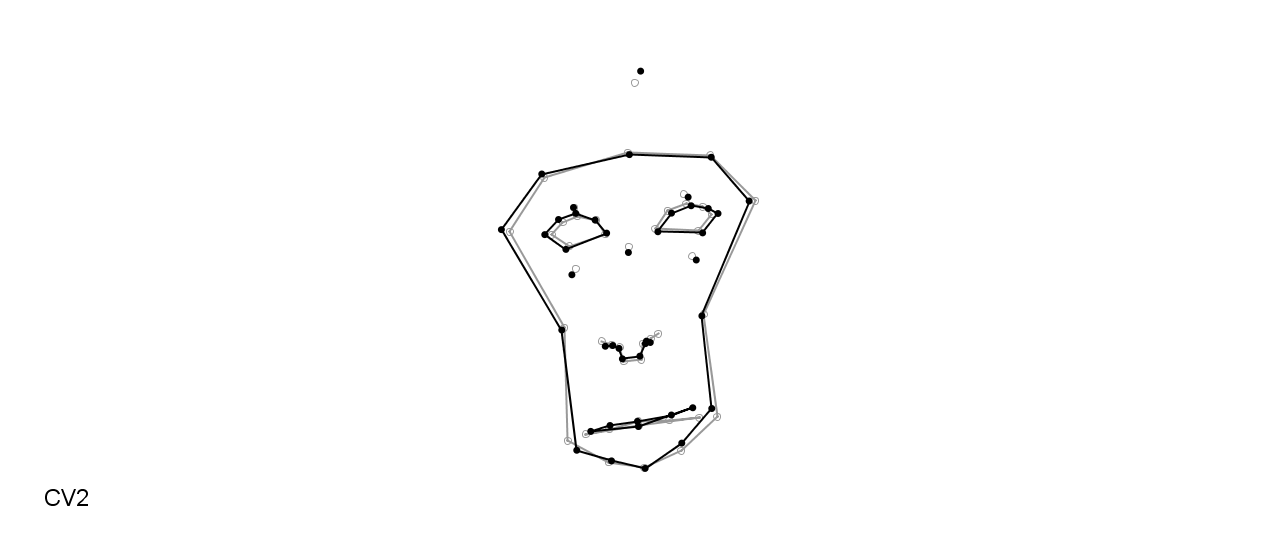

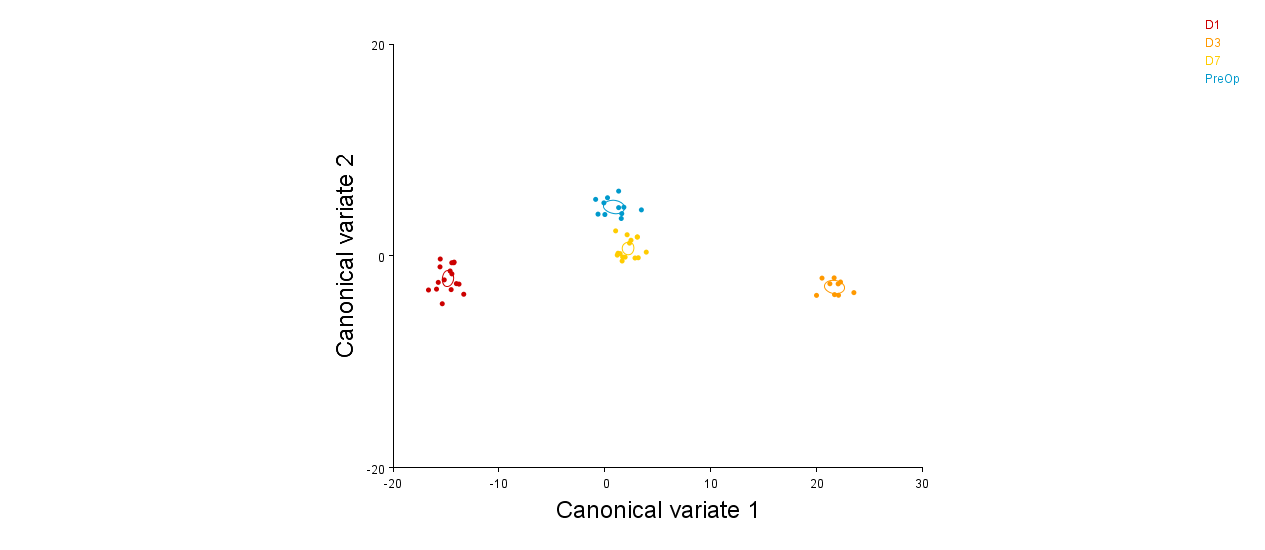

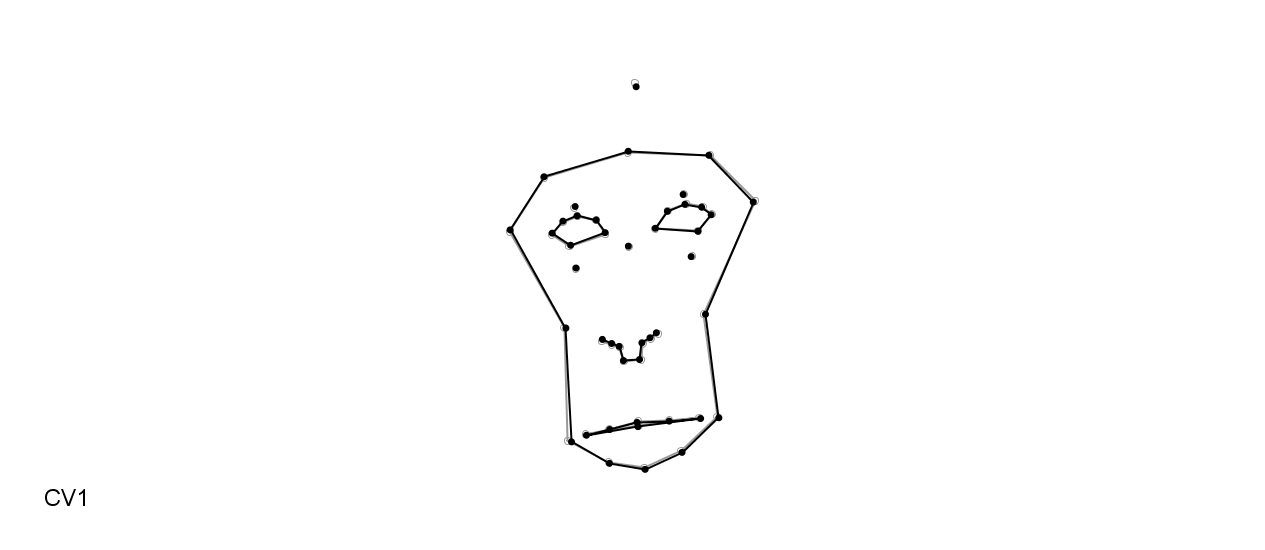

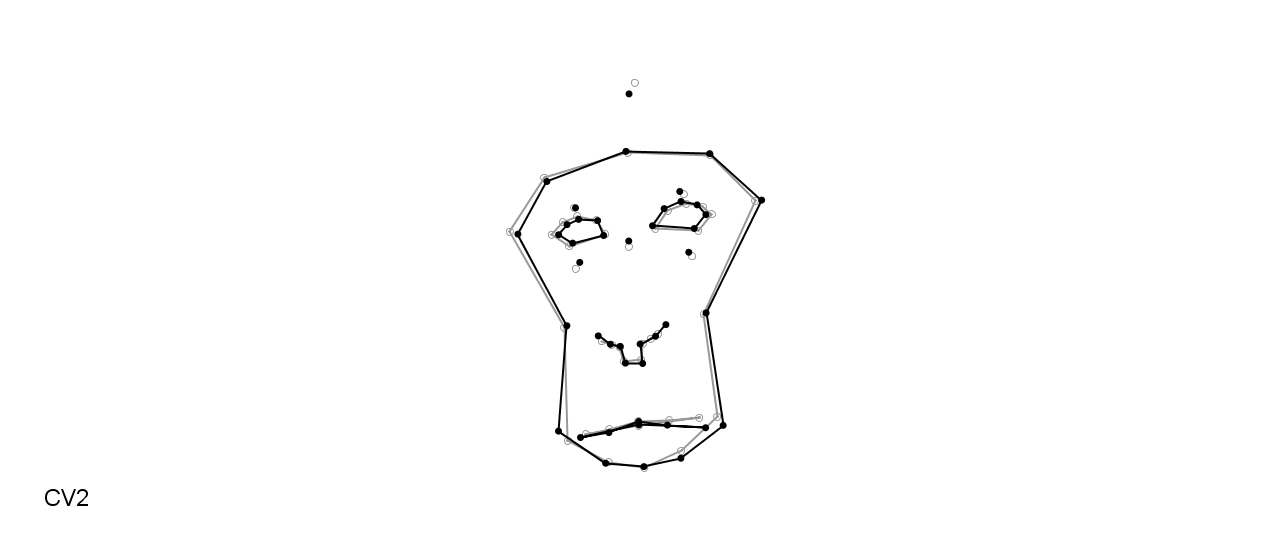

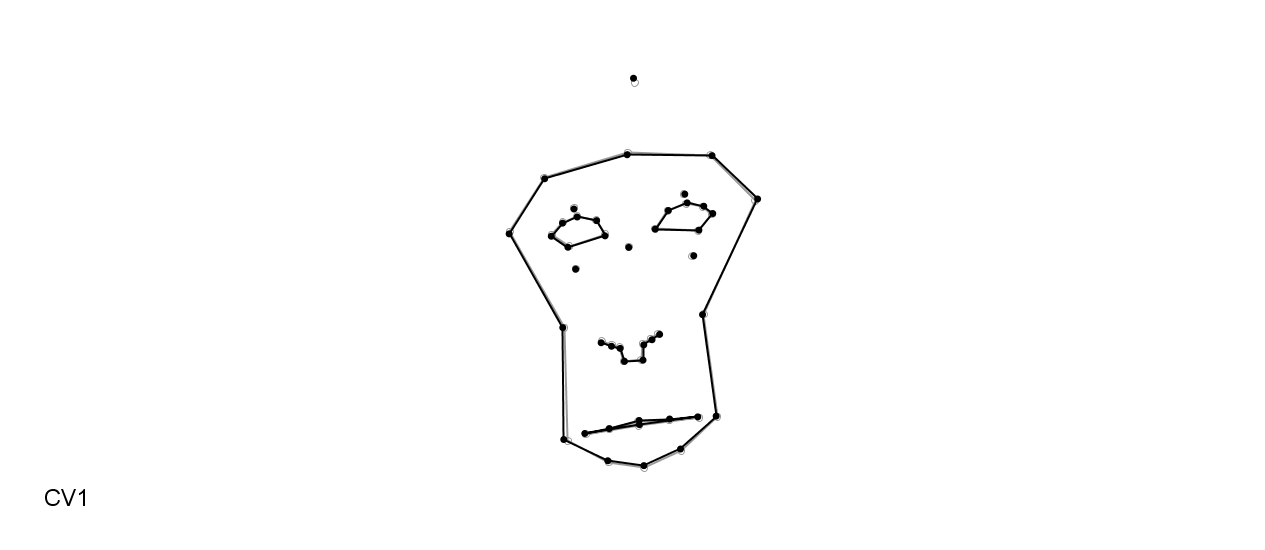


(3)

Pre

D1

D3

D7


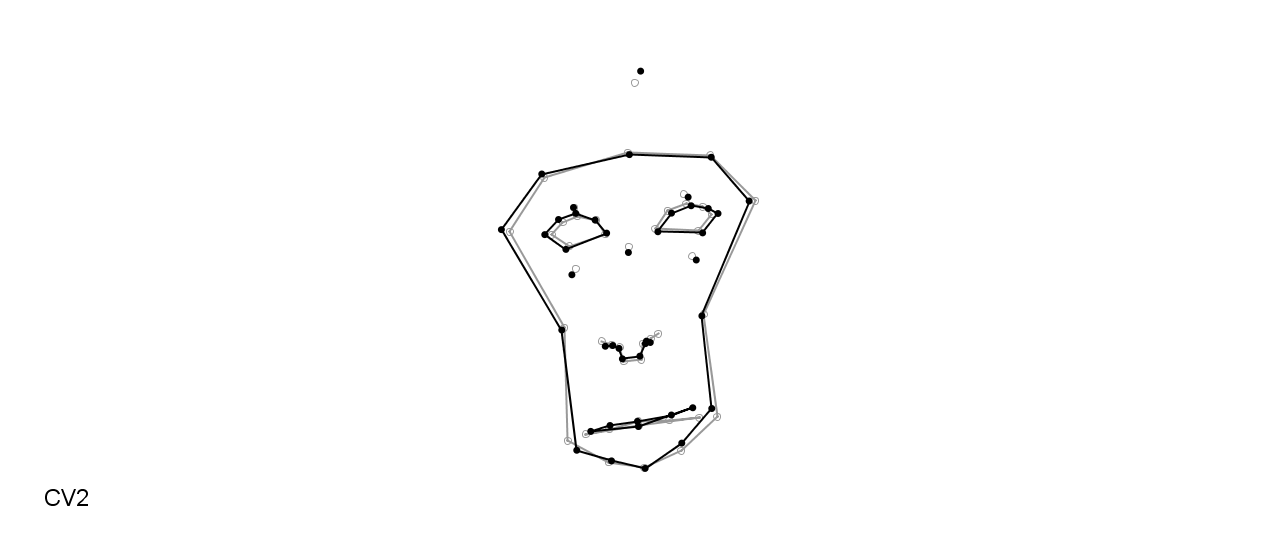

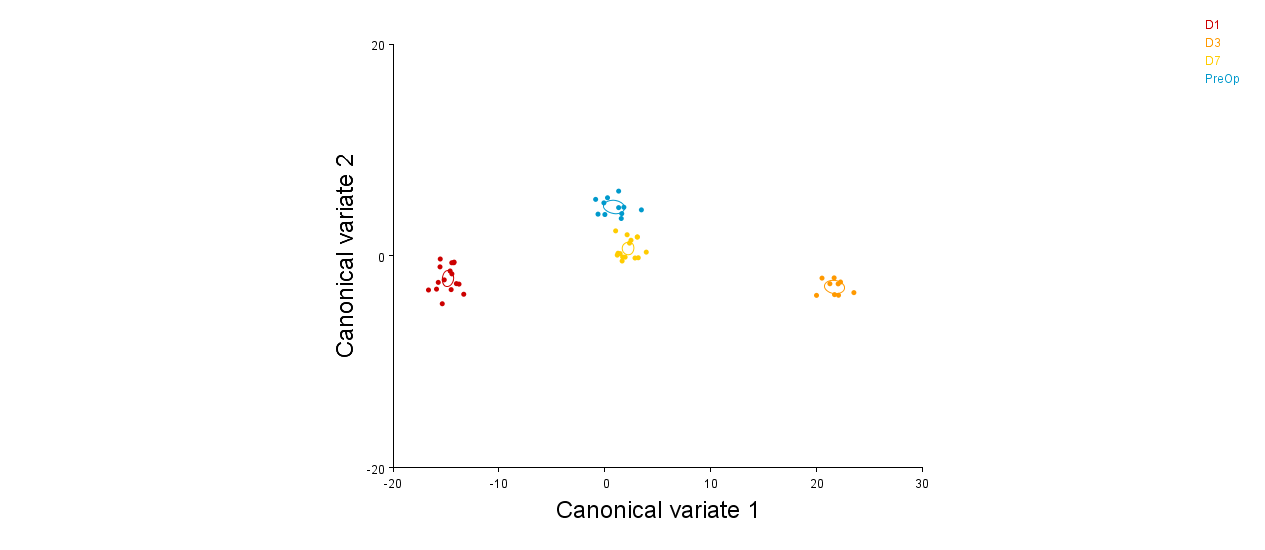

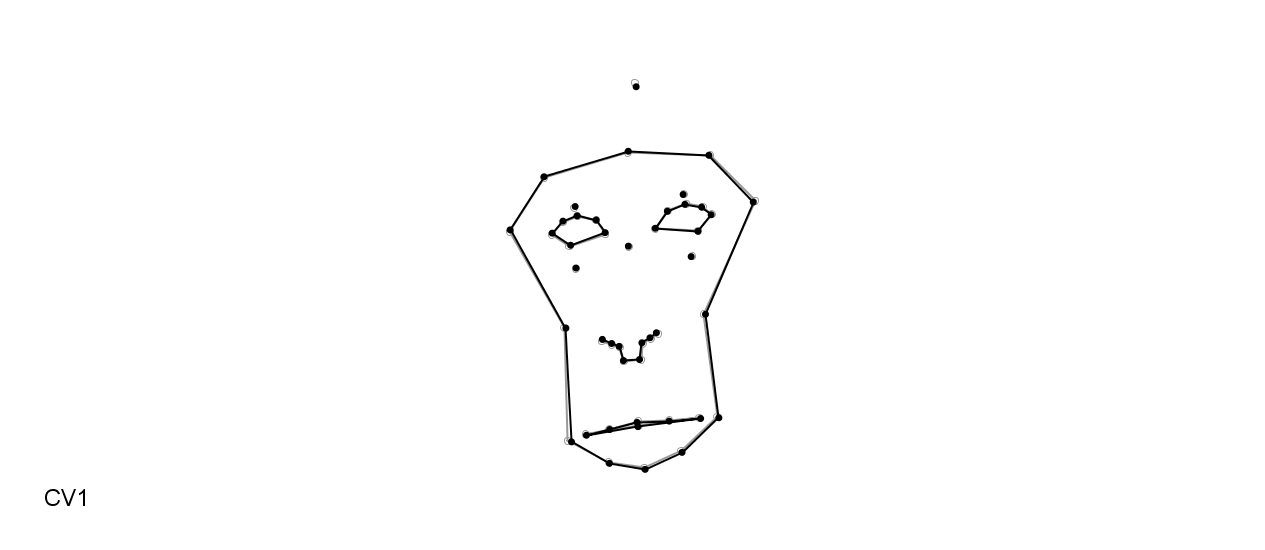

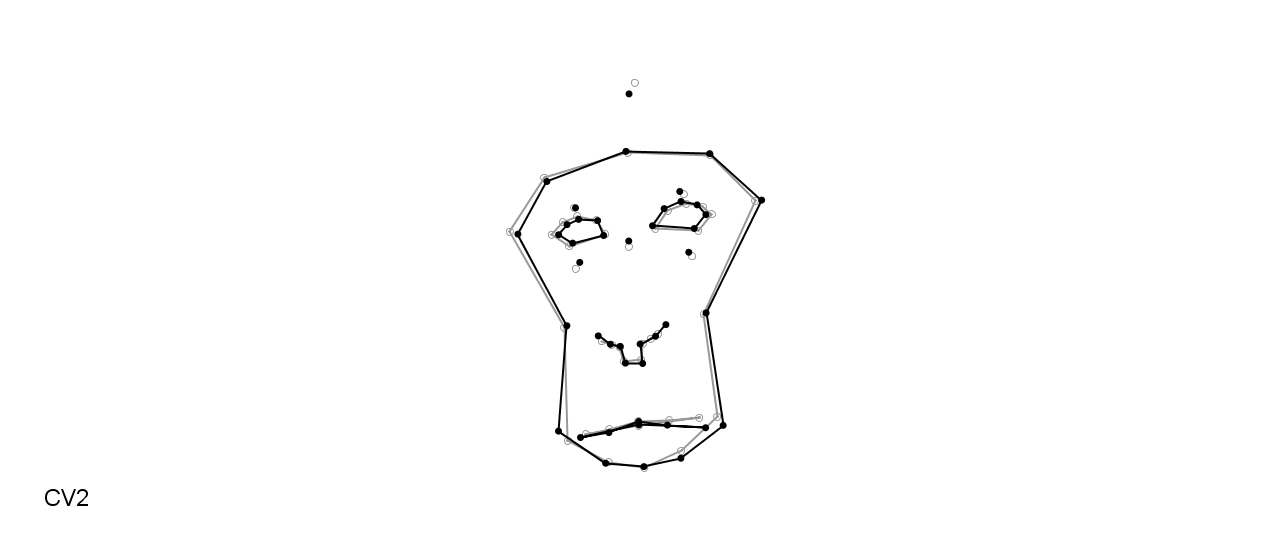

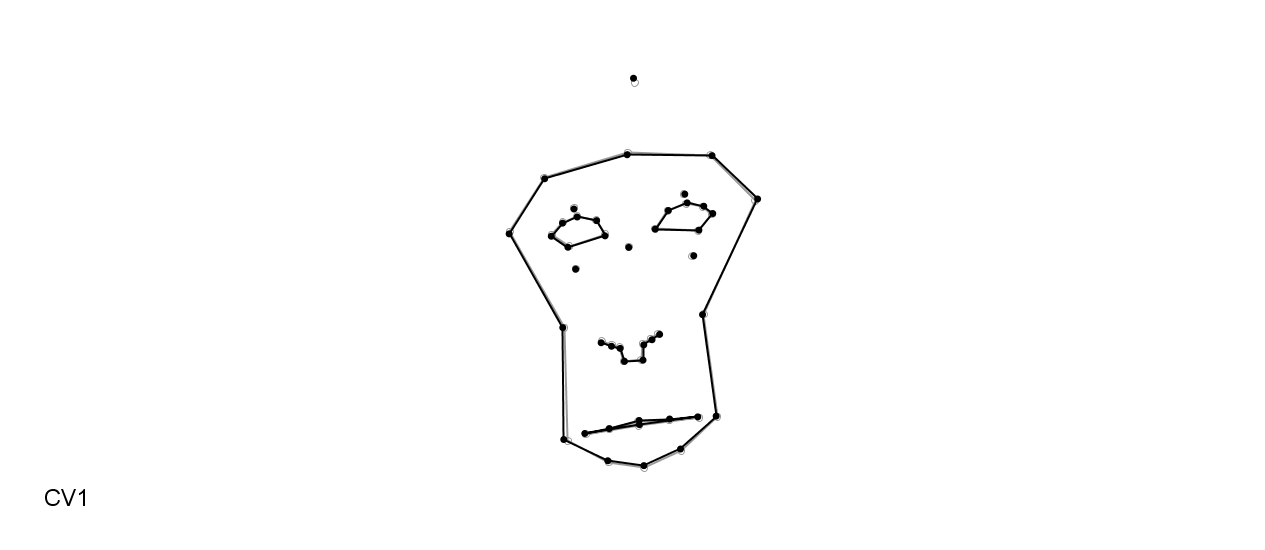

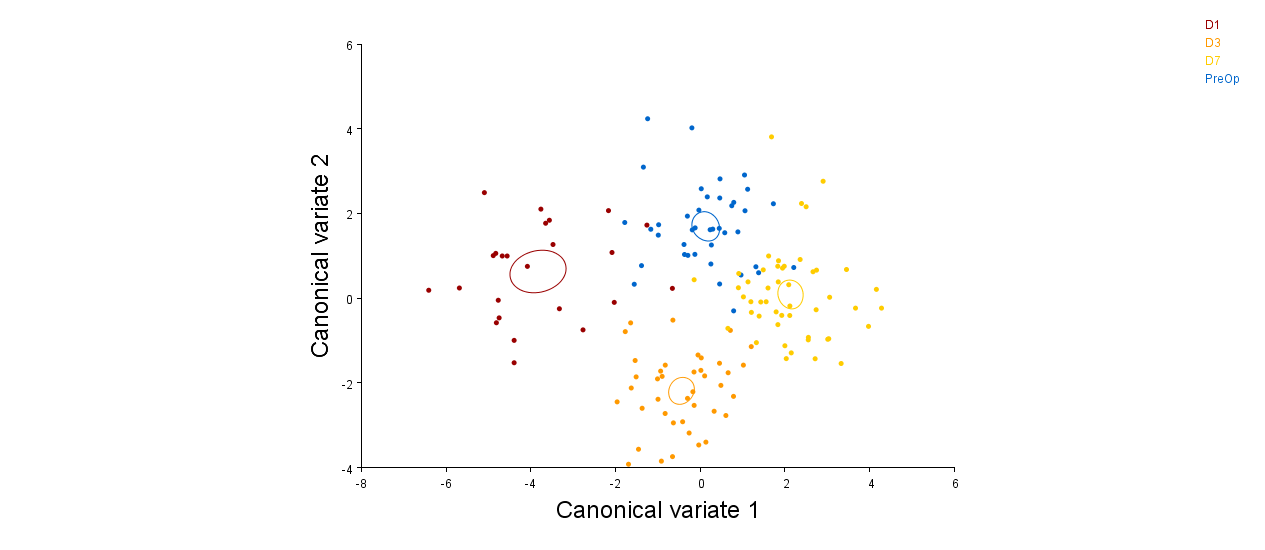

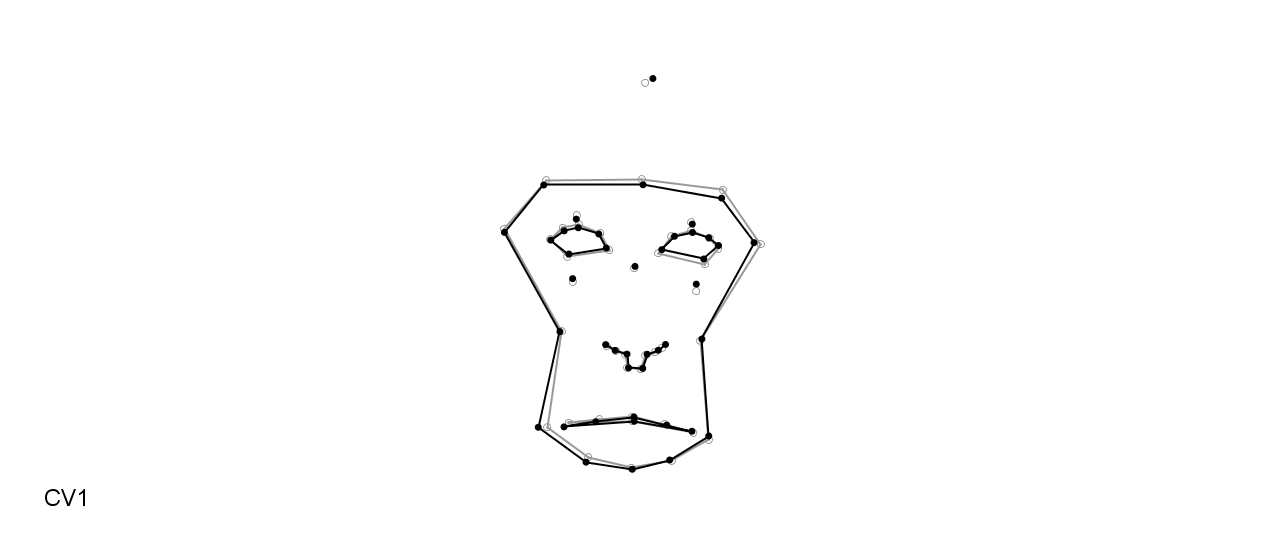

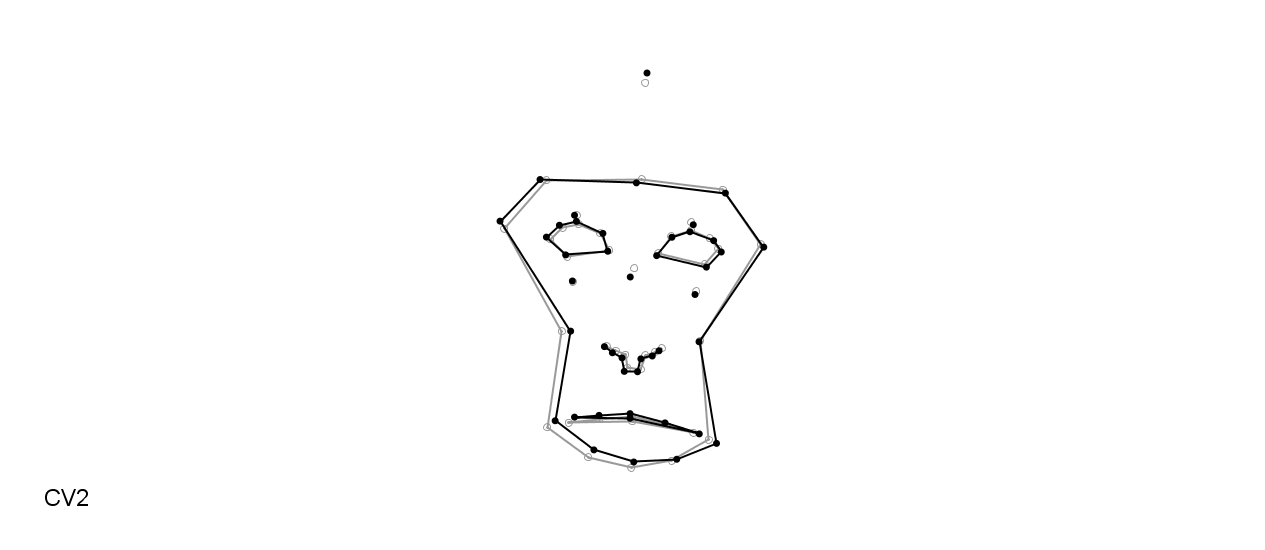

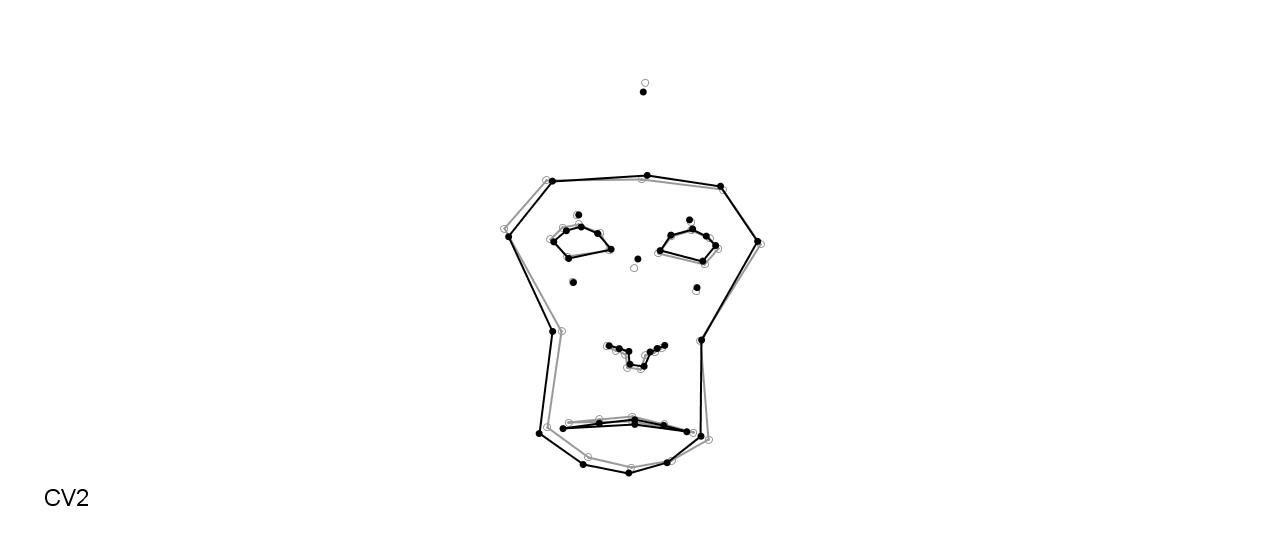

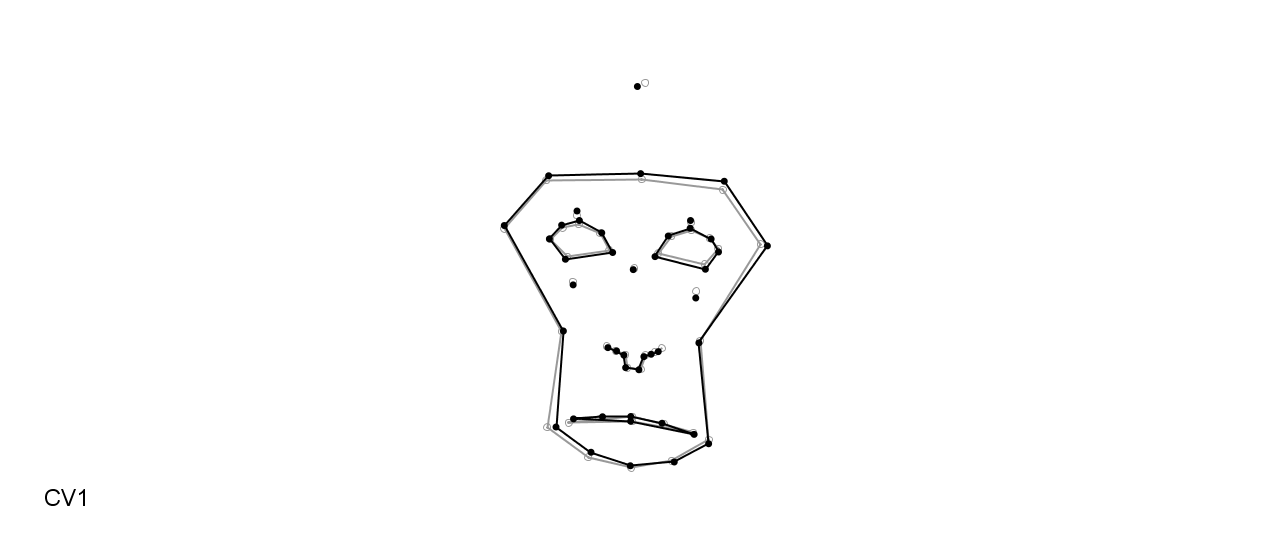


Pre

D1

D3

D7


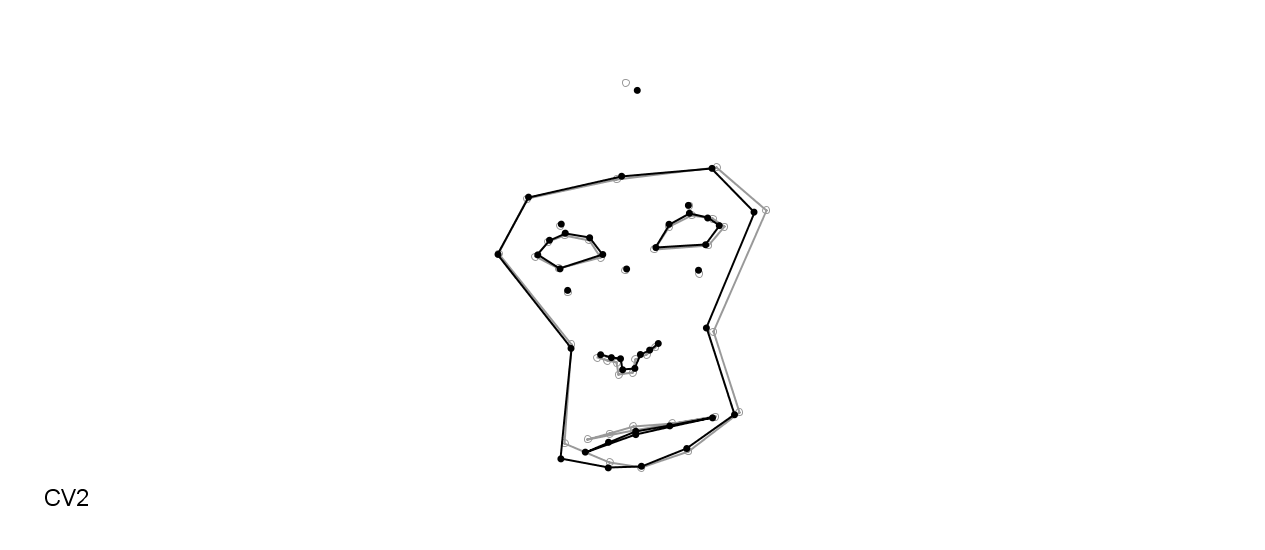

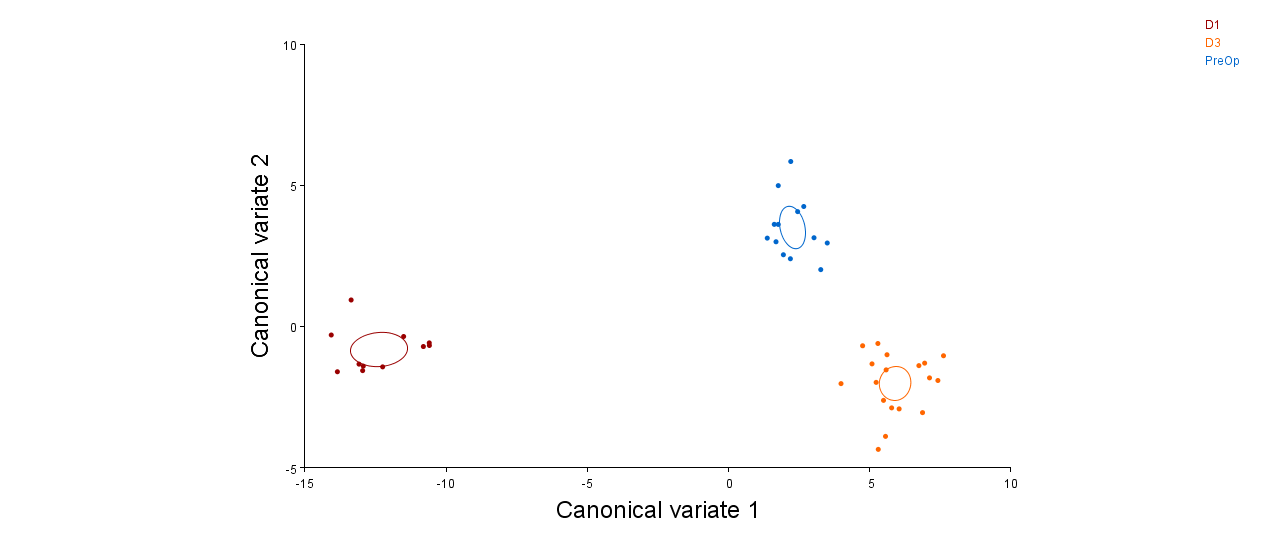

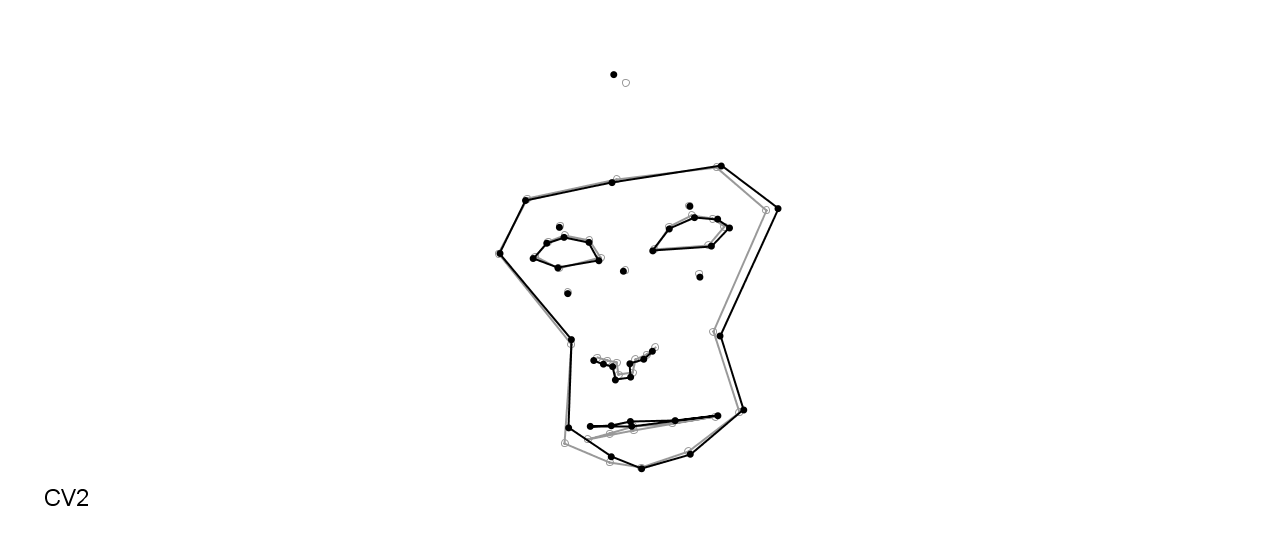

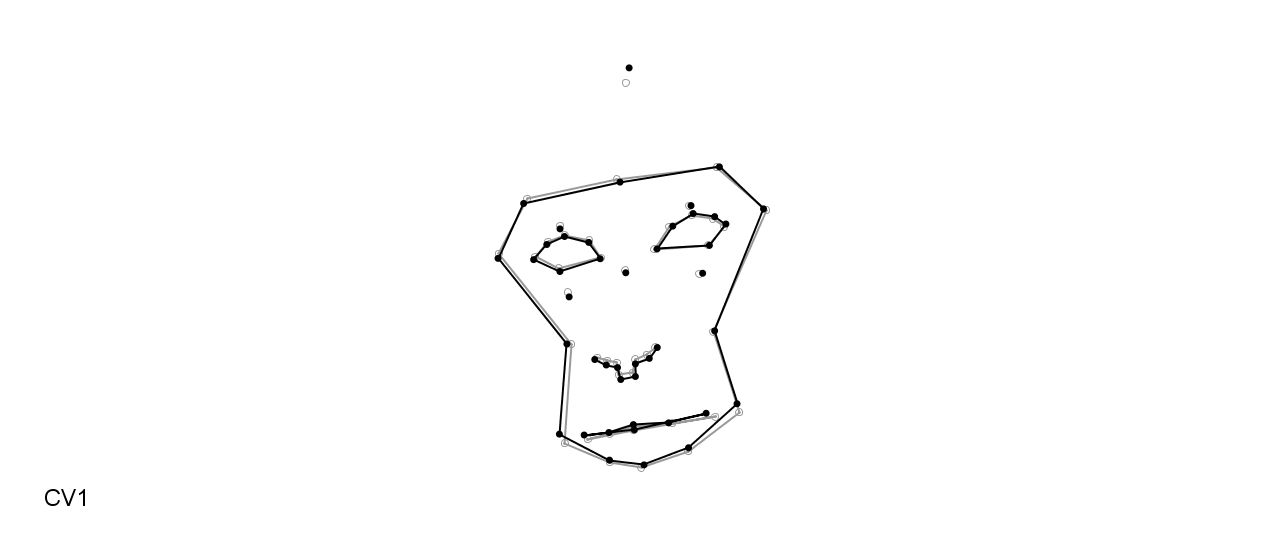

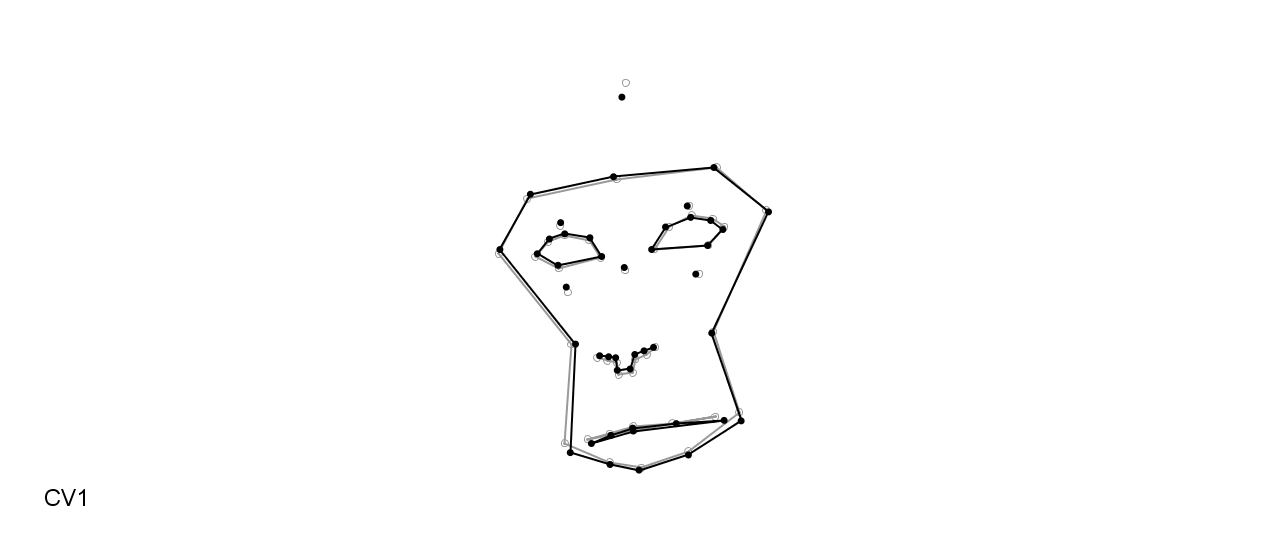


Pre

D1

D3

(6)

(5)

(8)


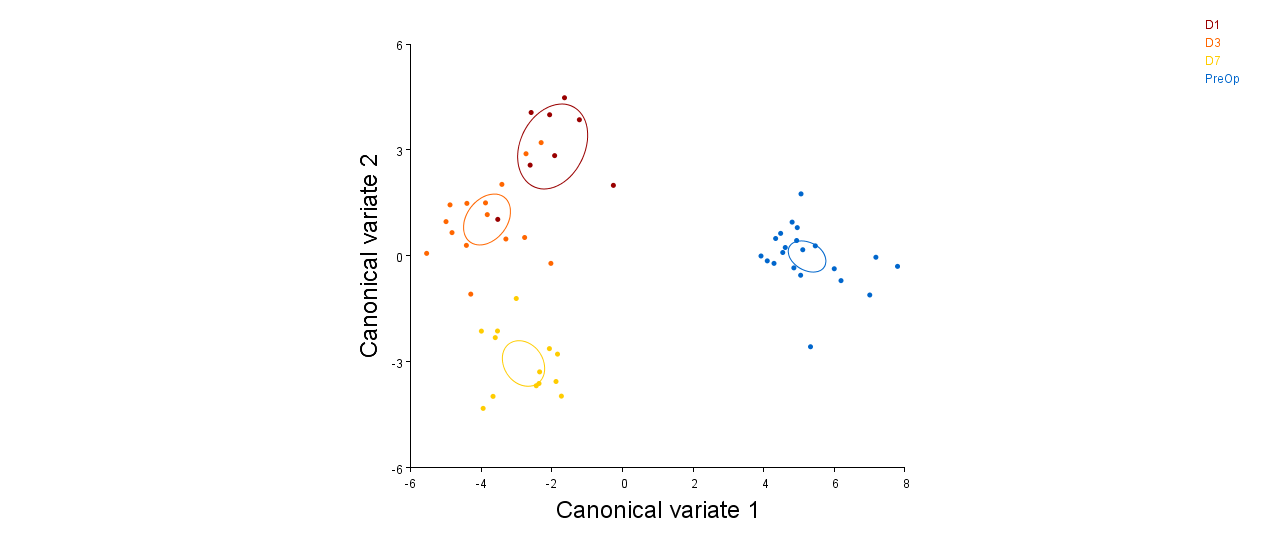

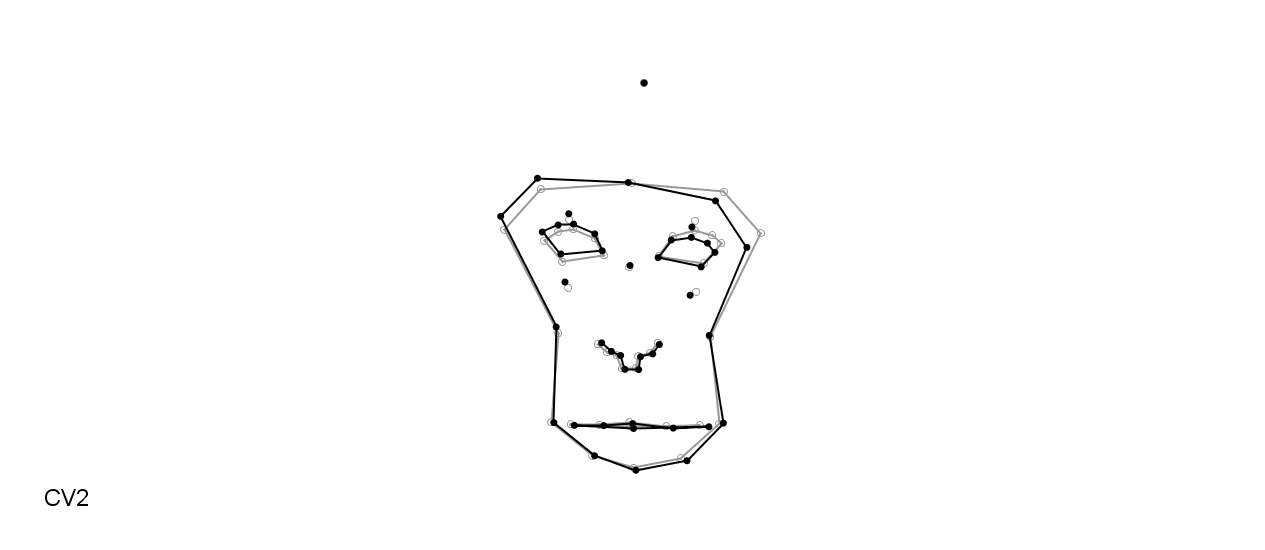

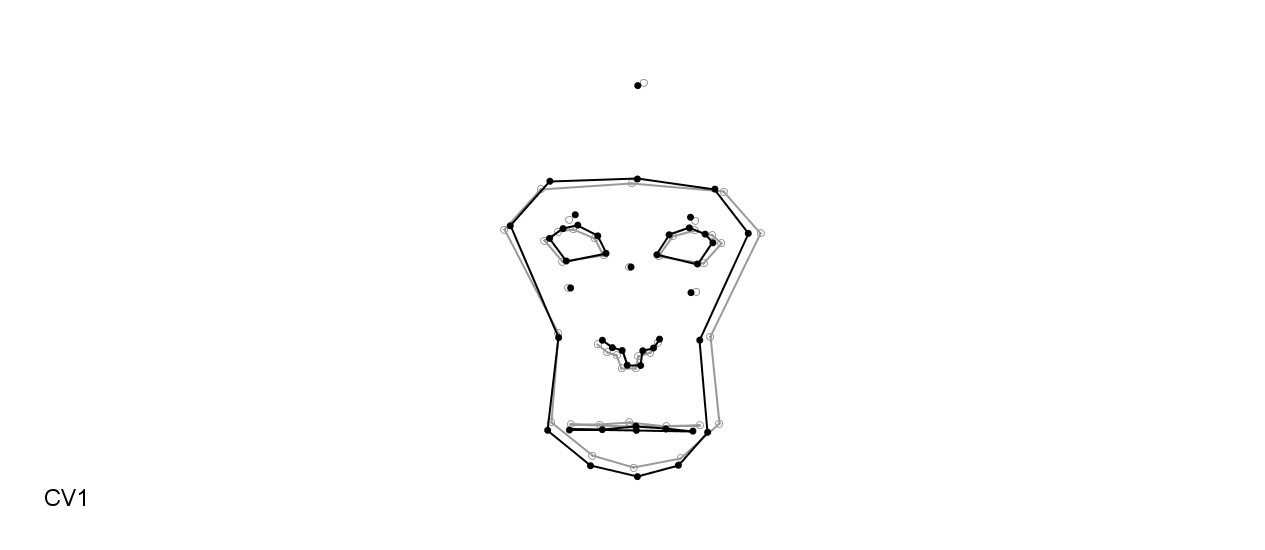

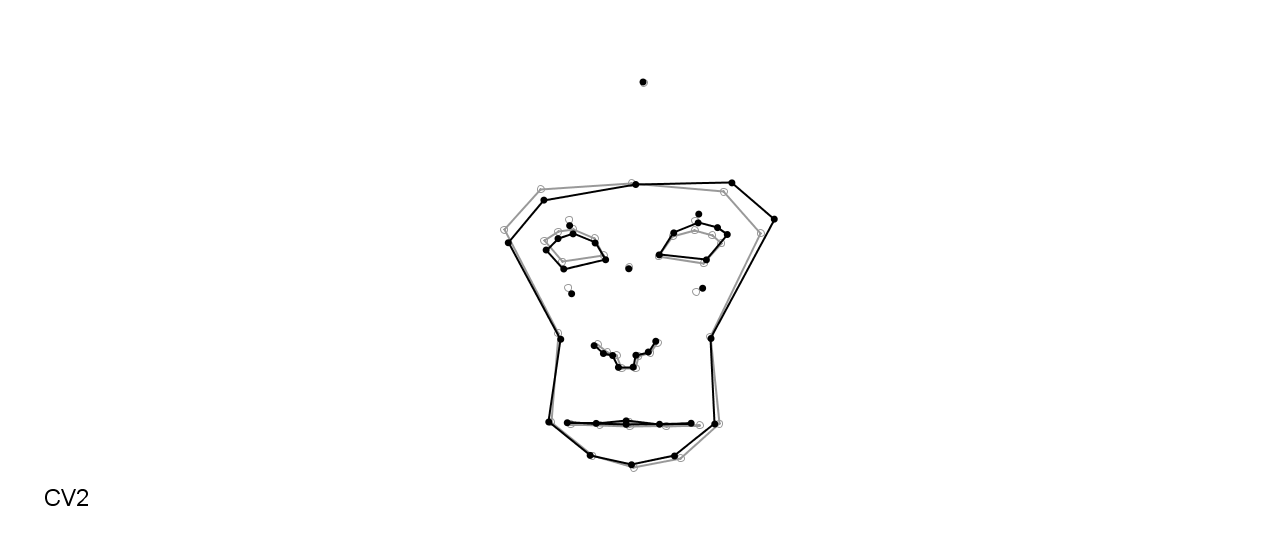

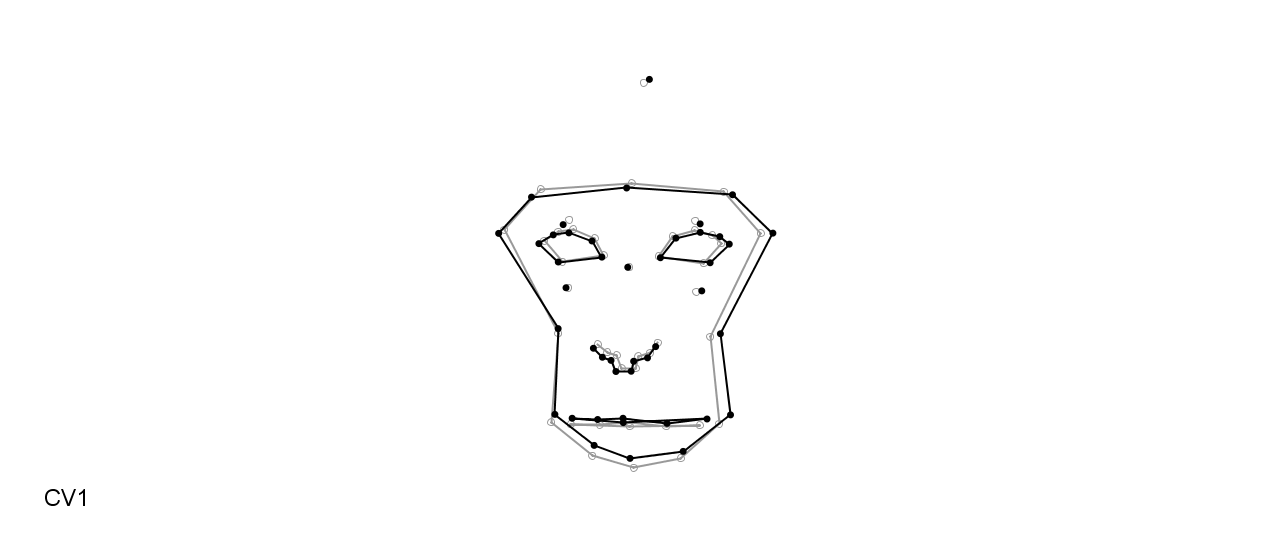


Pre

D1

D3

D7

(7)


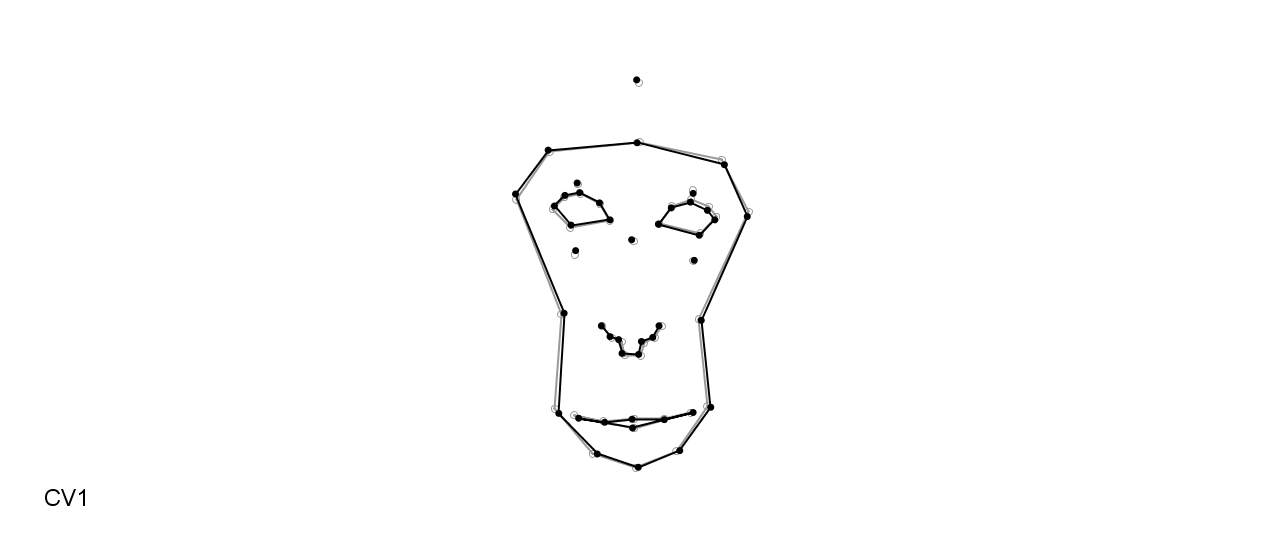

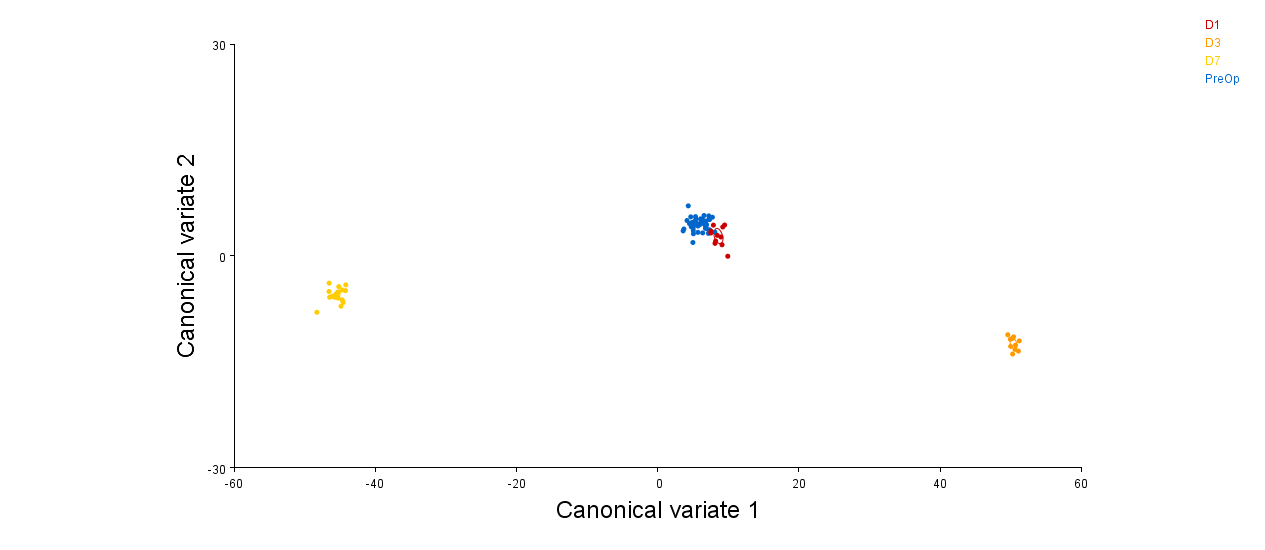

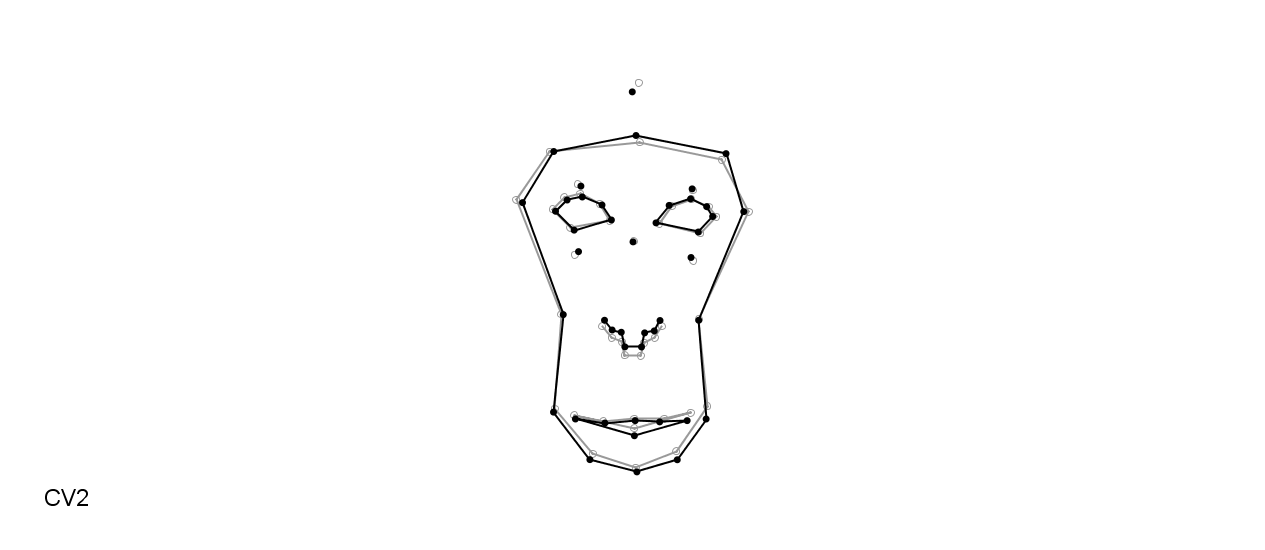

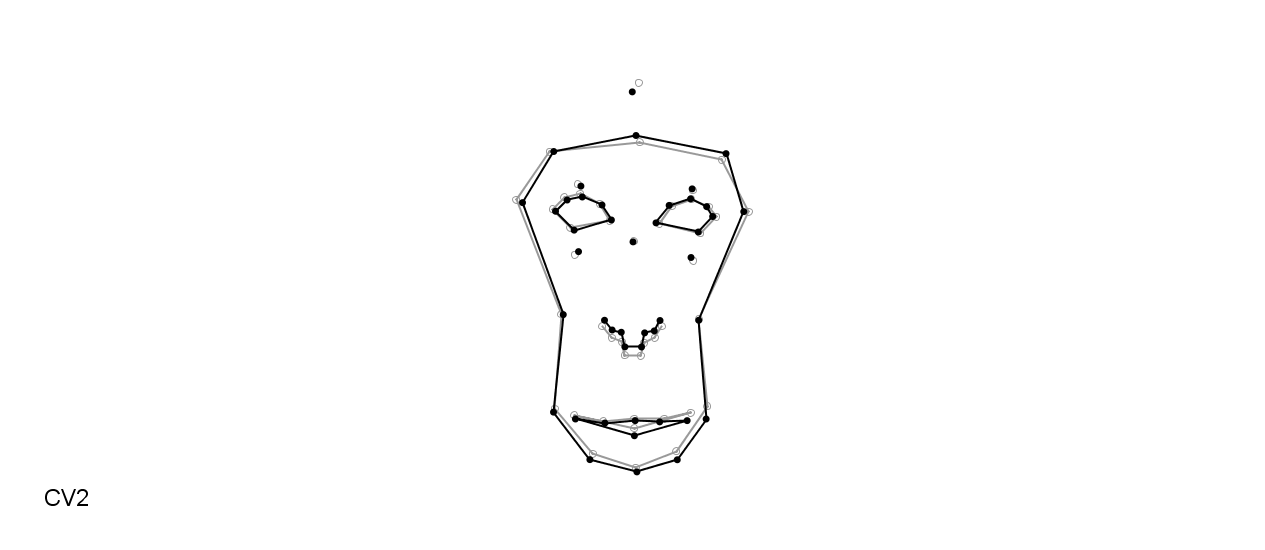

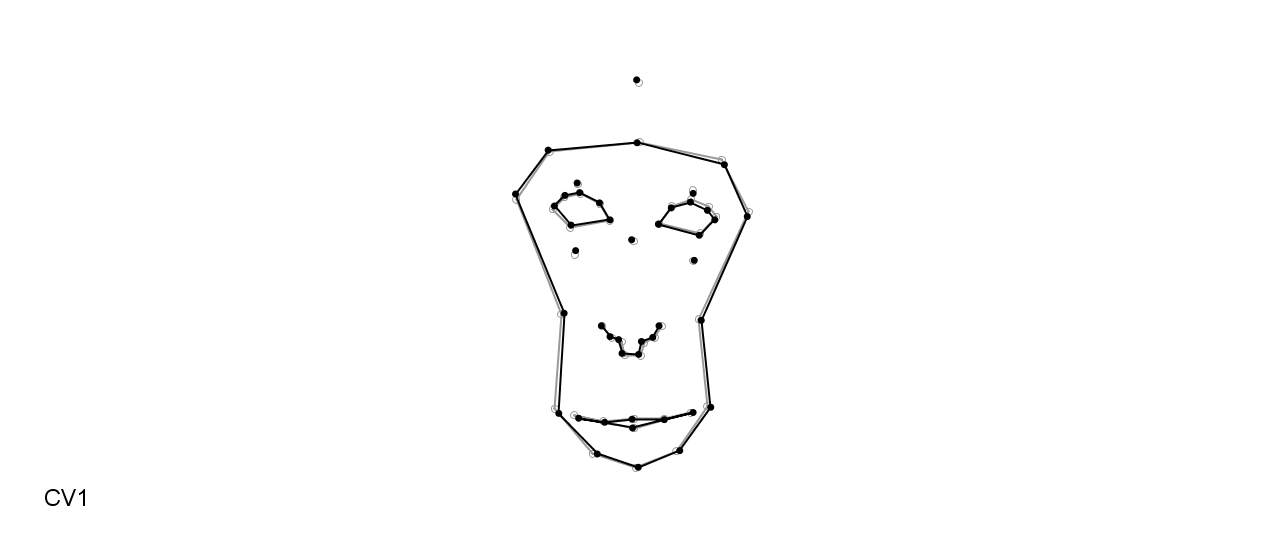


Pre

D1

D3

D7

(9)


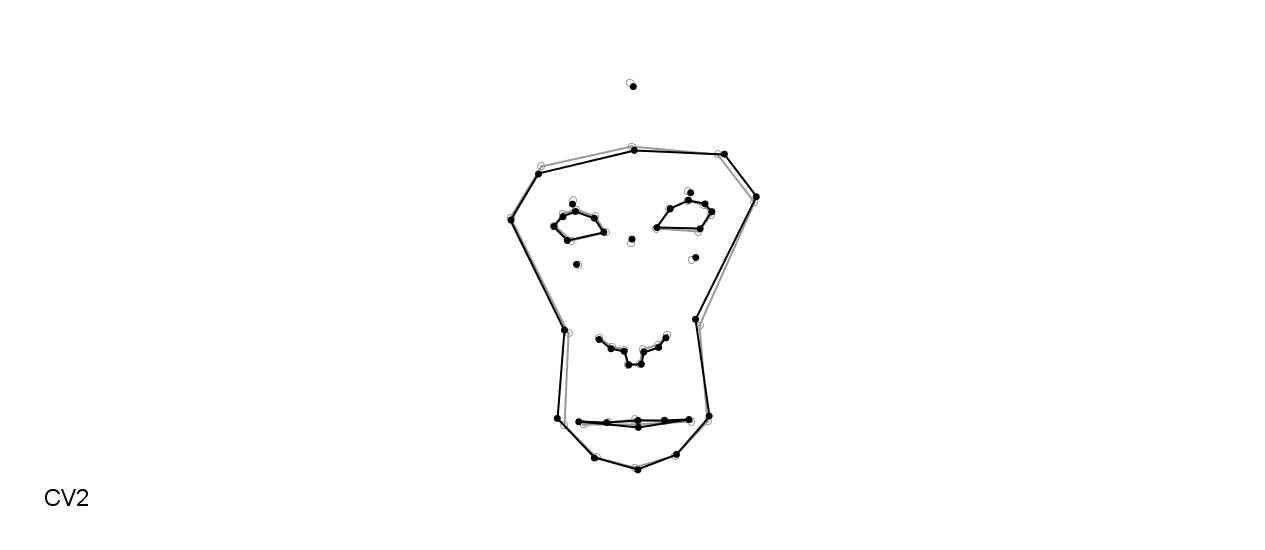

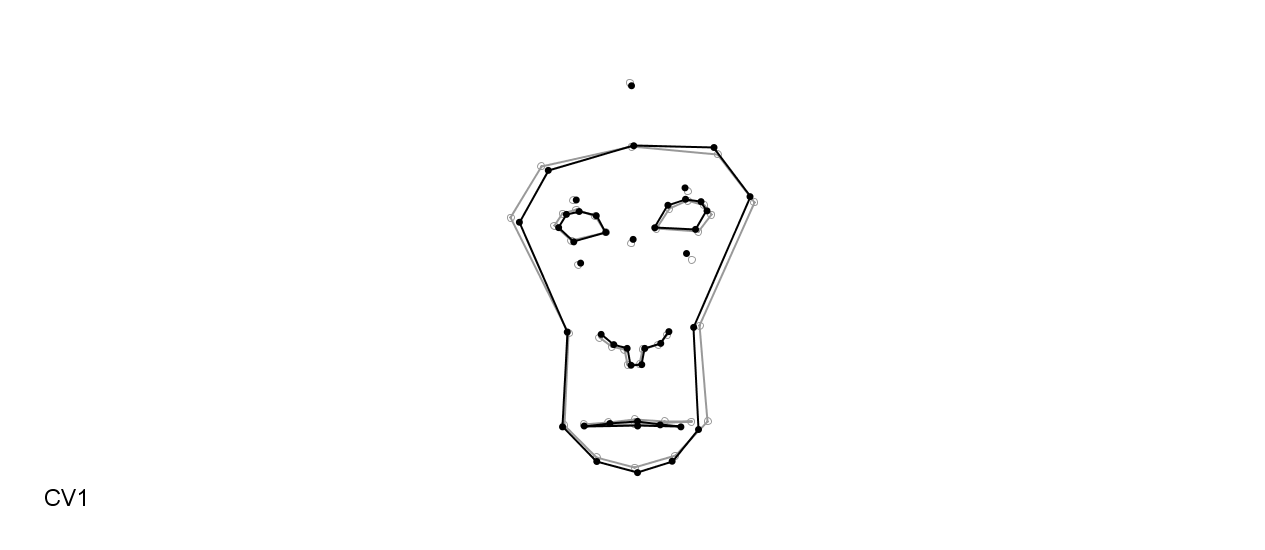

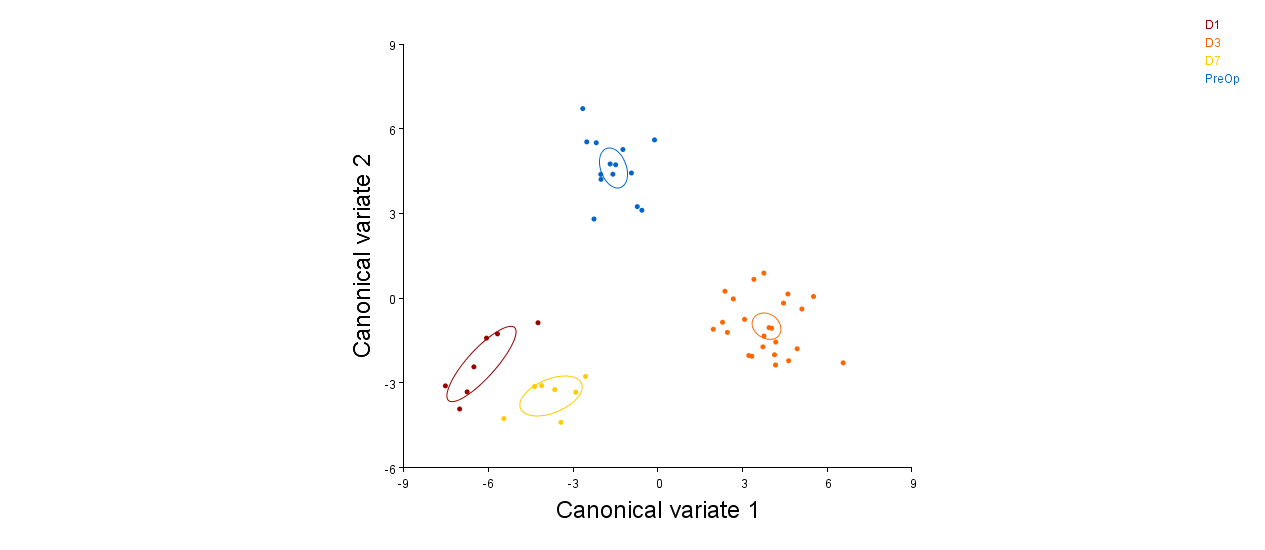

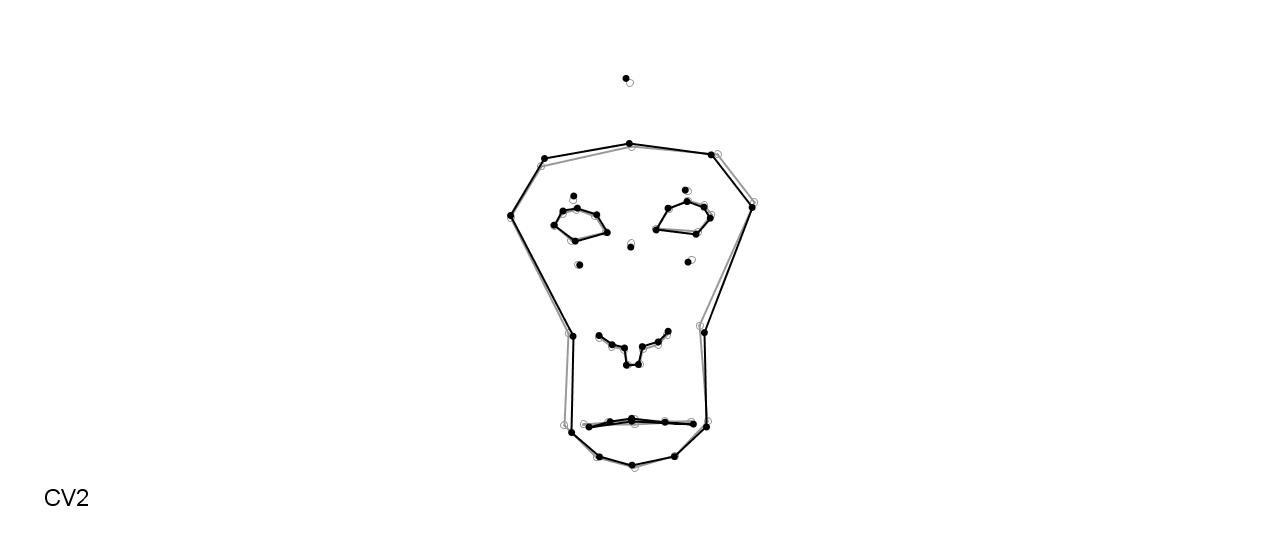

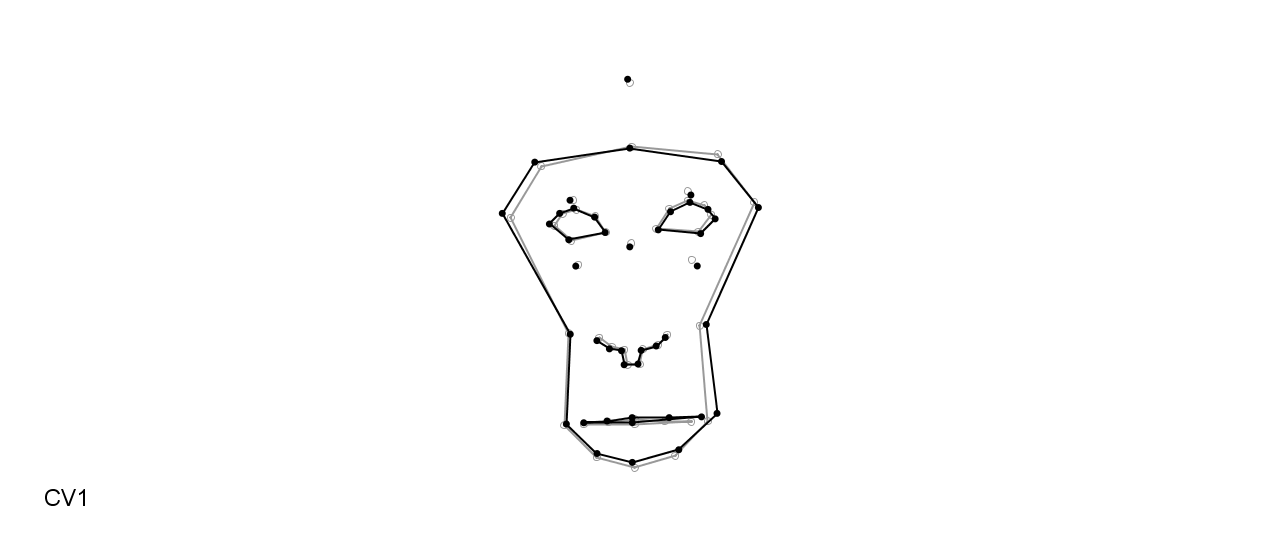


Pre

D1

D3

D7


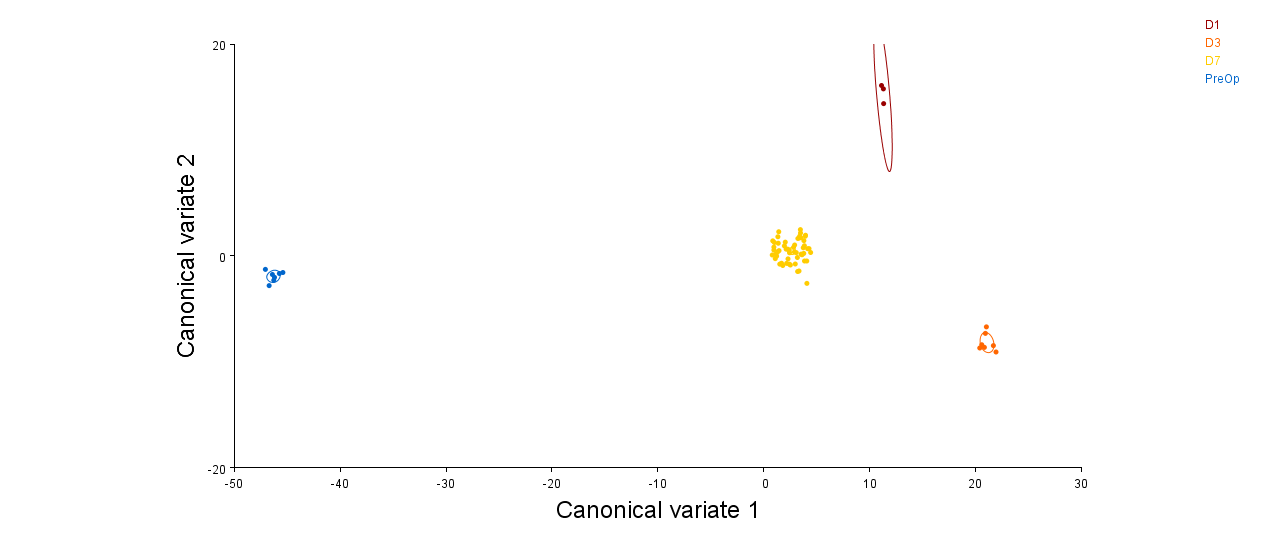

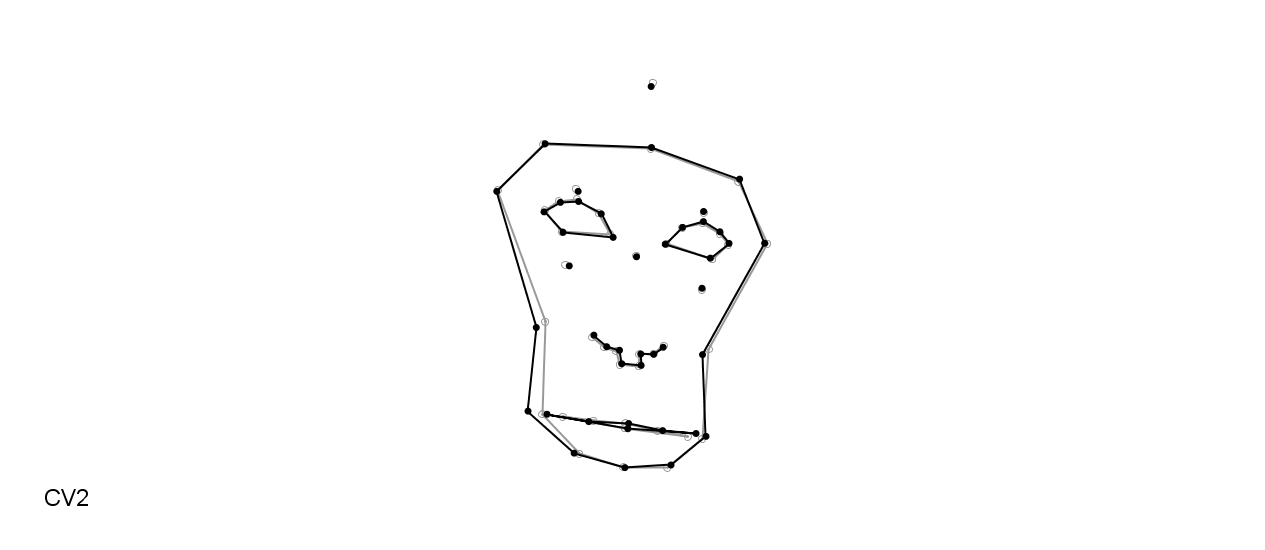

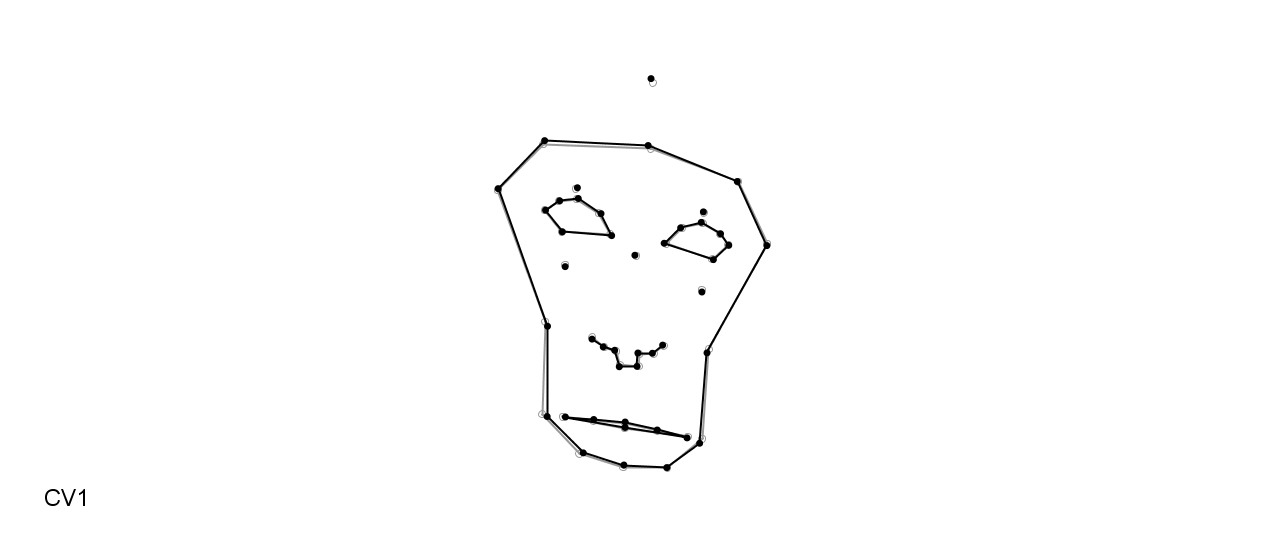

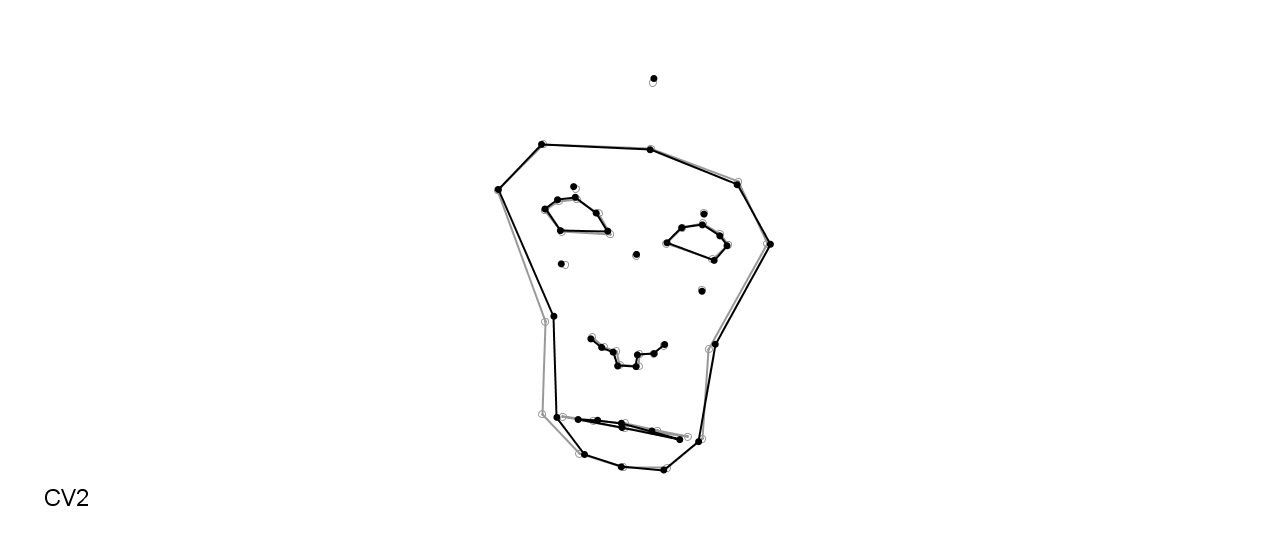

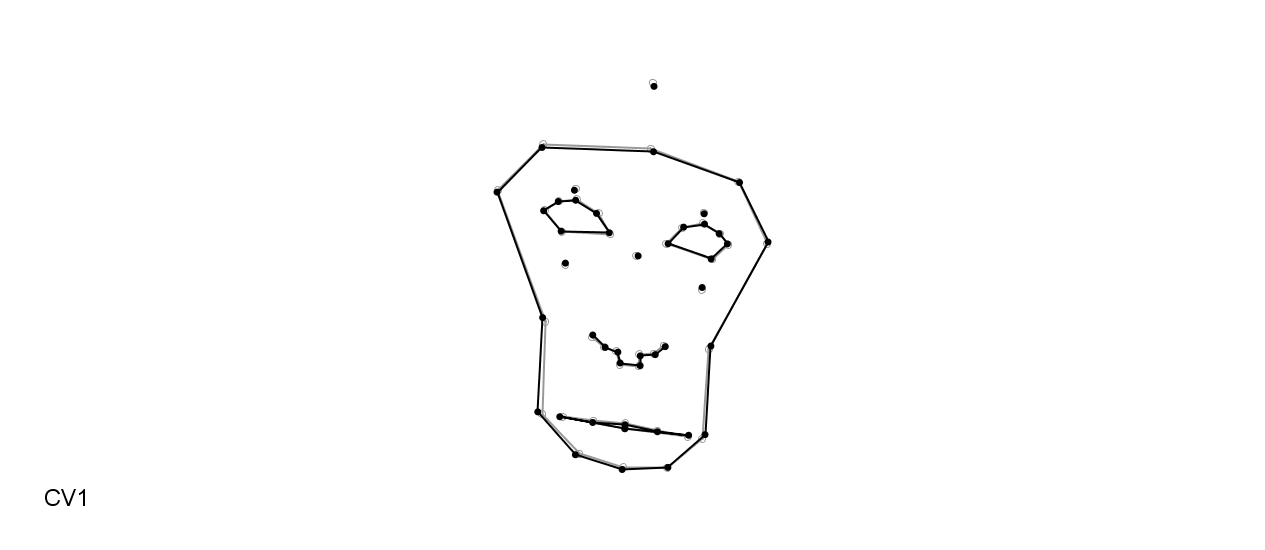


(10)

Pre

D1

D3

D7


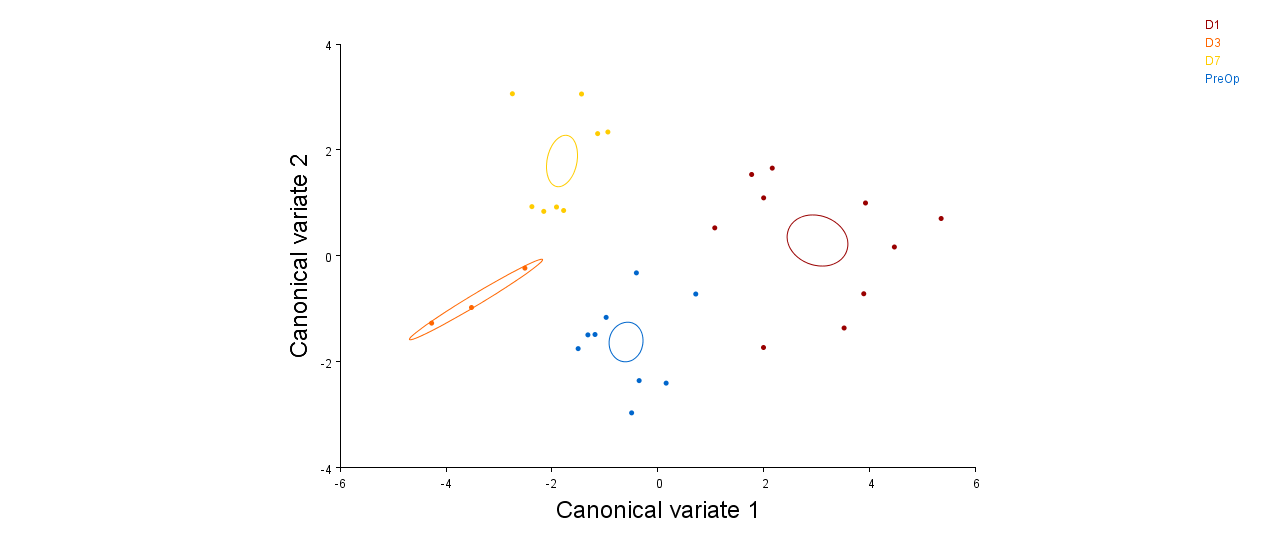

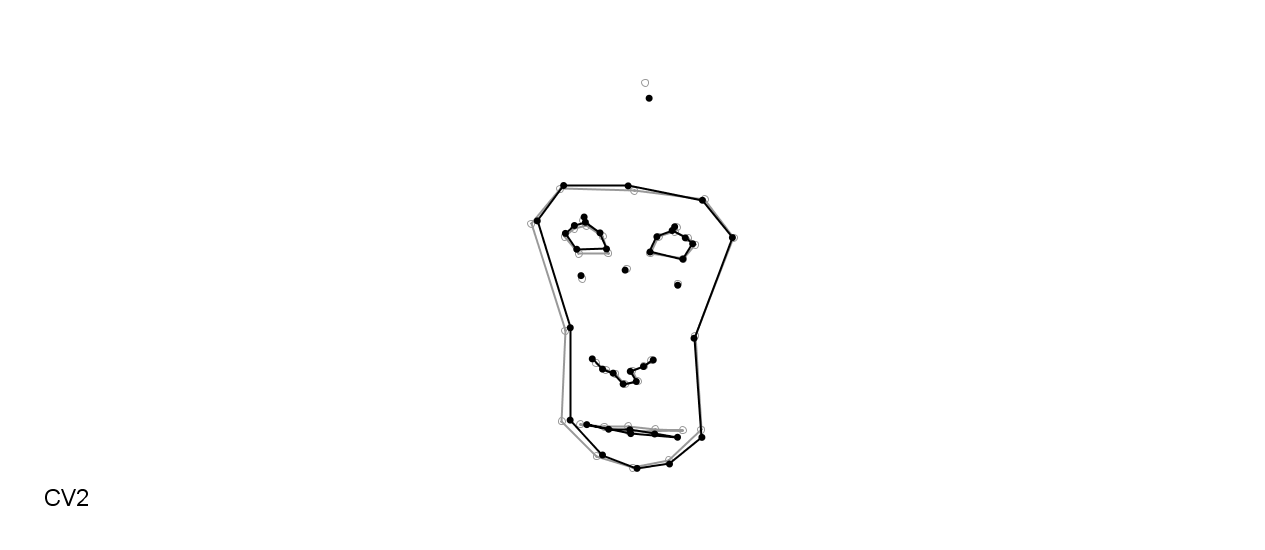

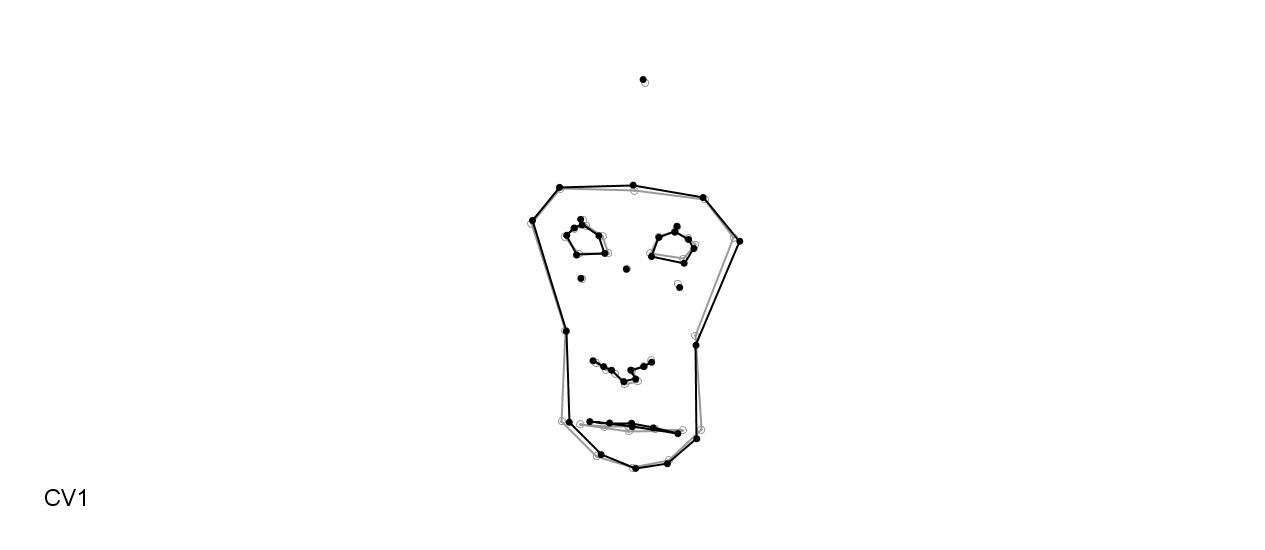

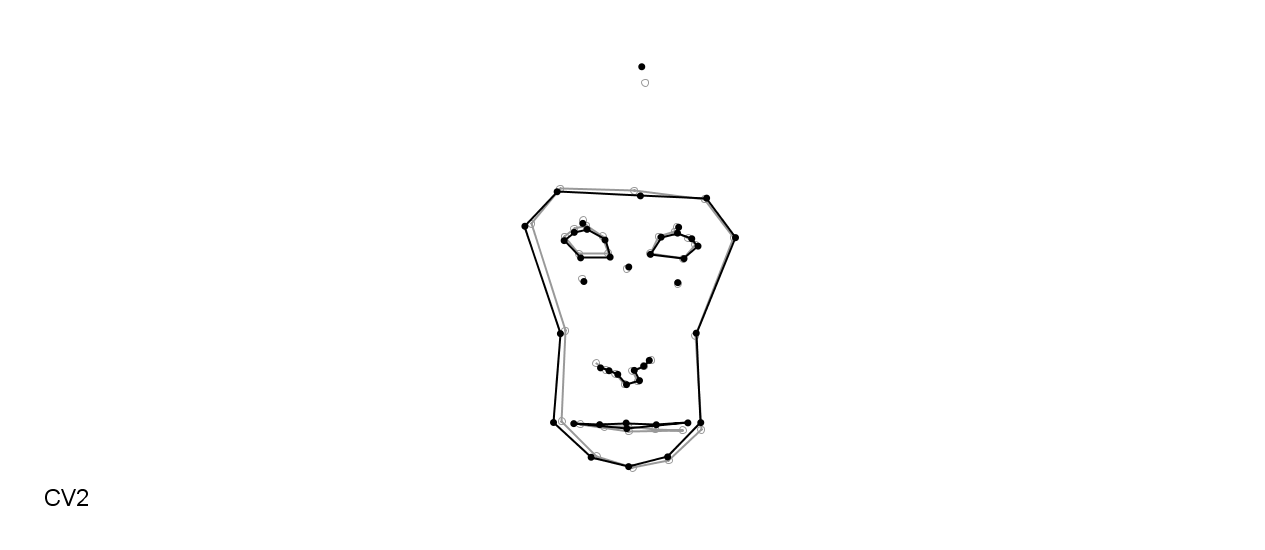

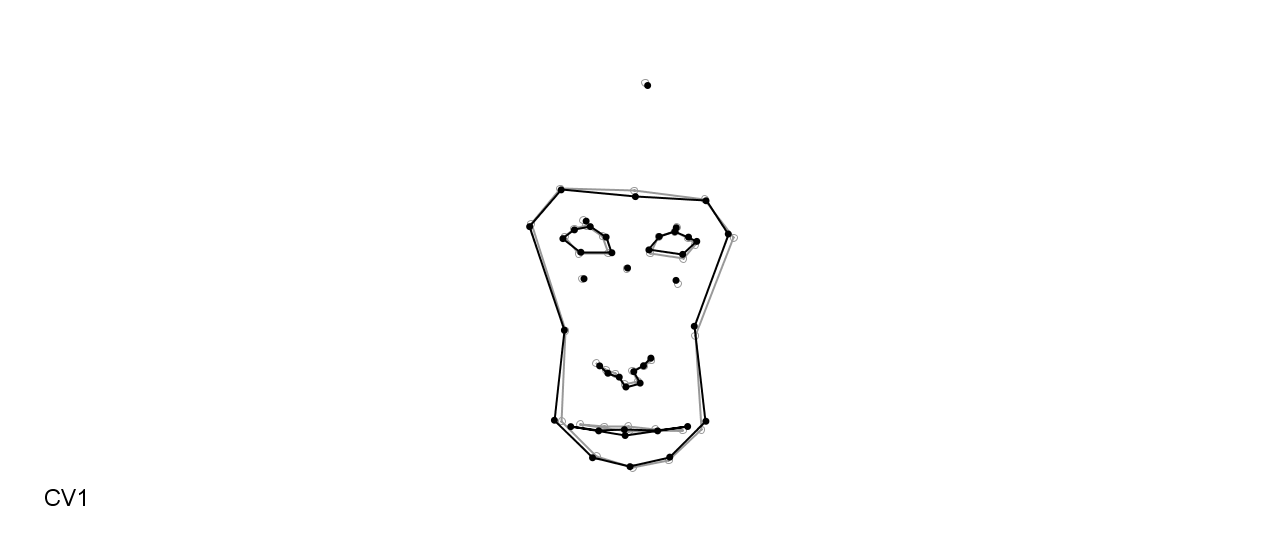


(12)

Pre

D1

D3

D7


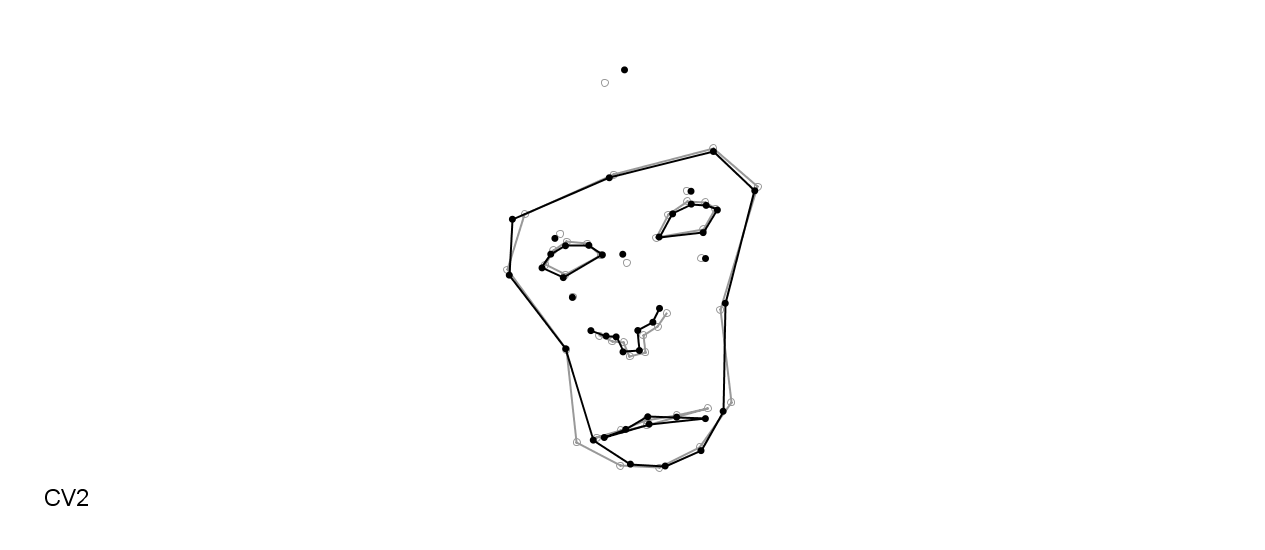

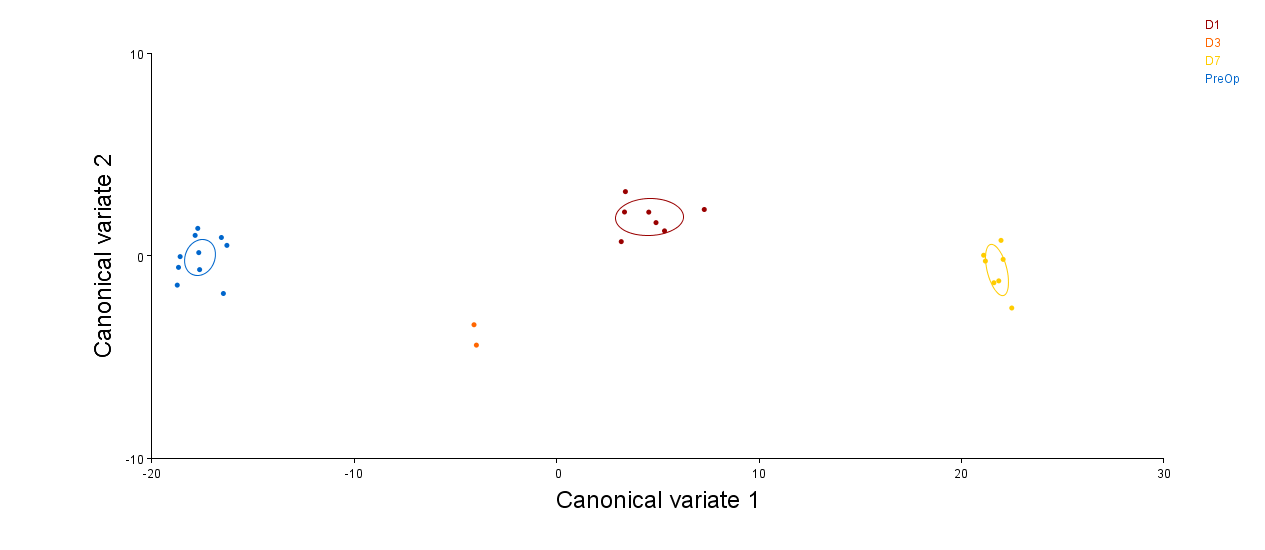

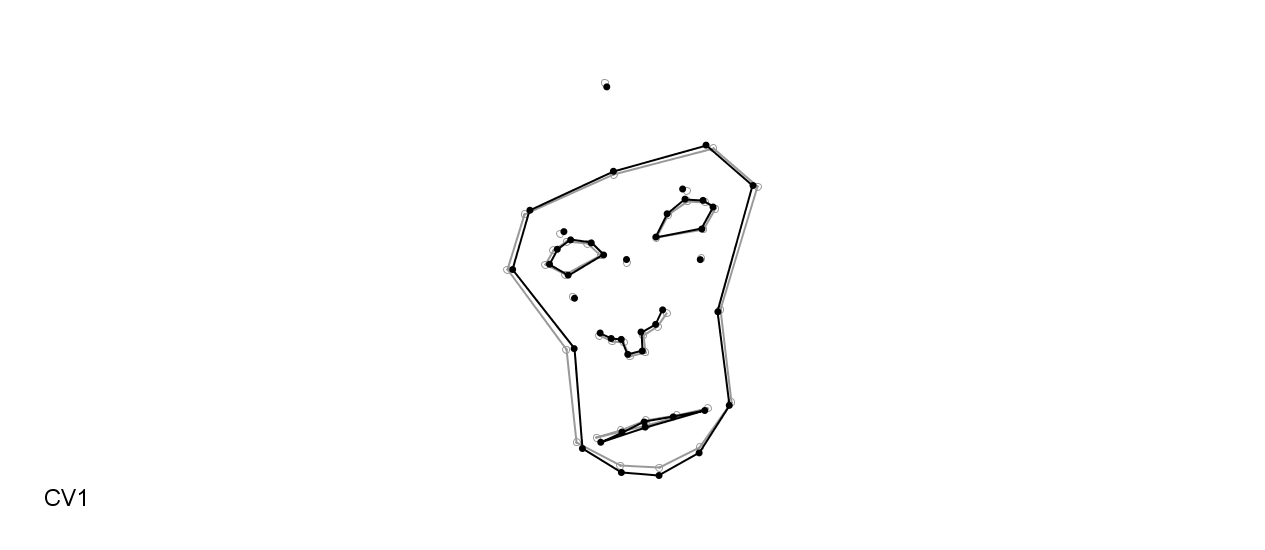

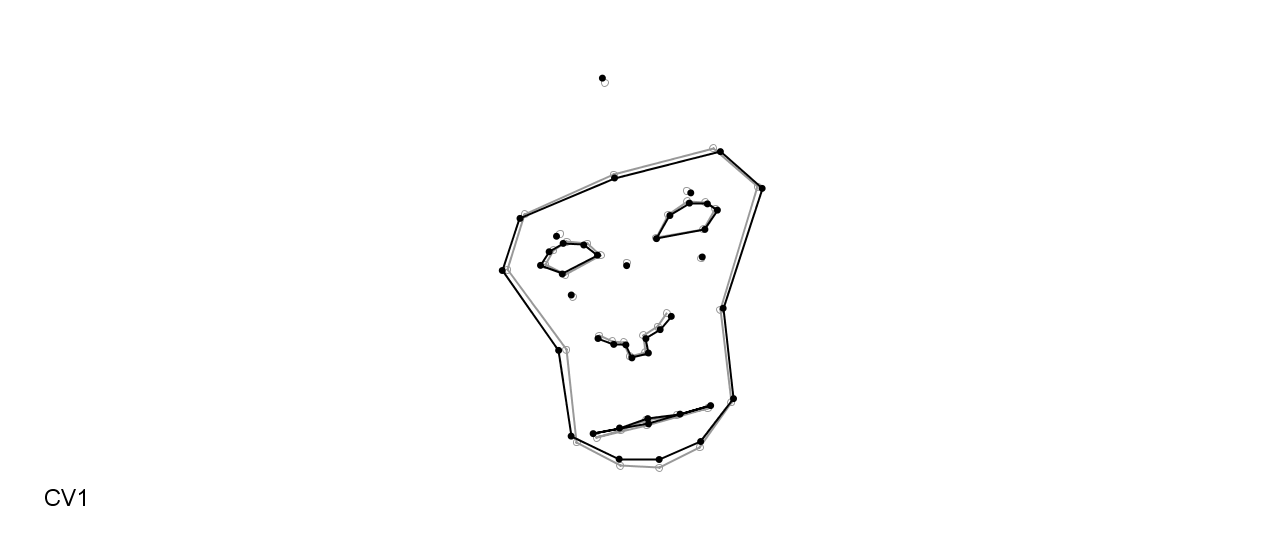

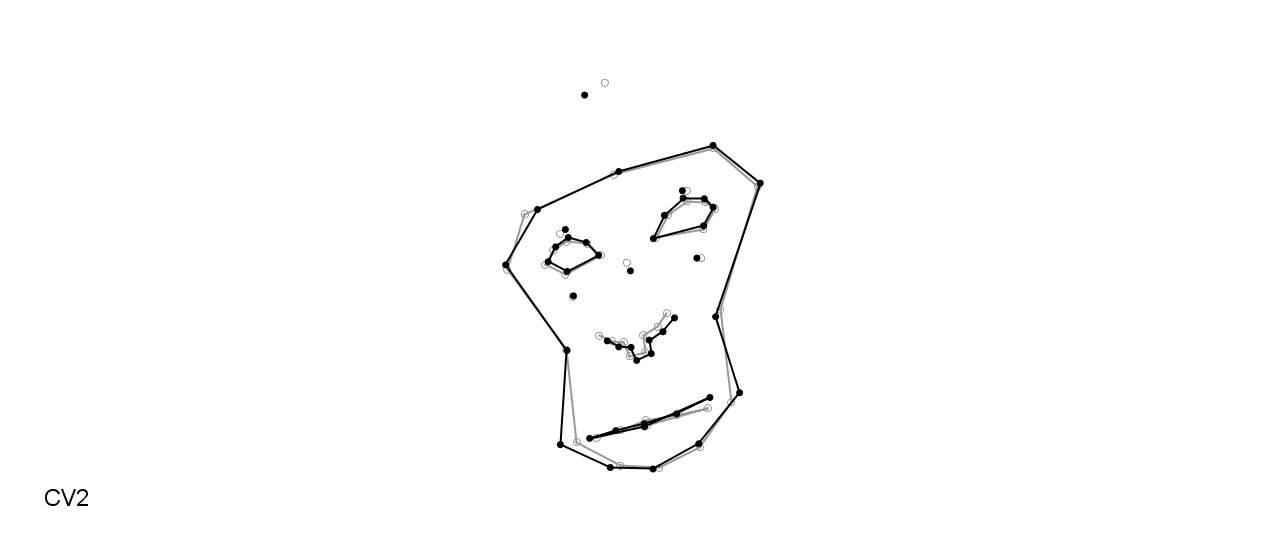


(11)

Pre

D1

D3

D7


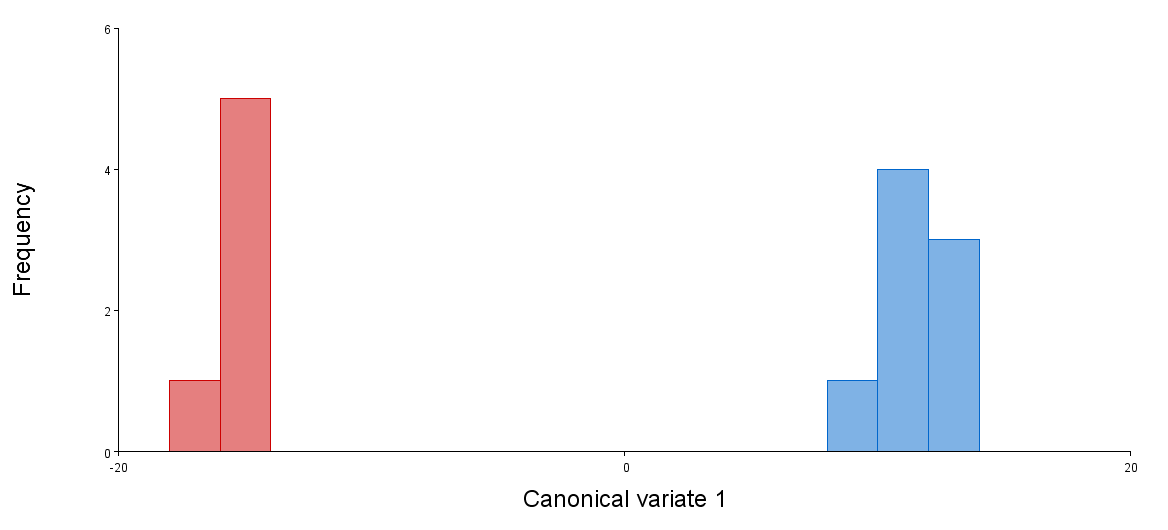

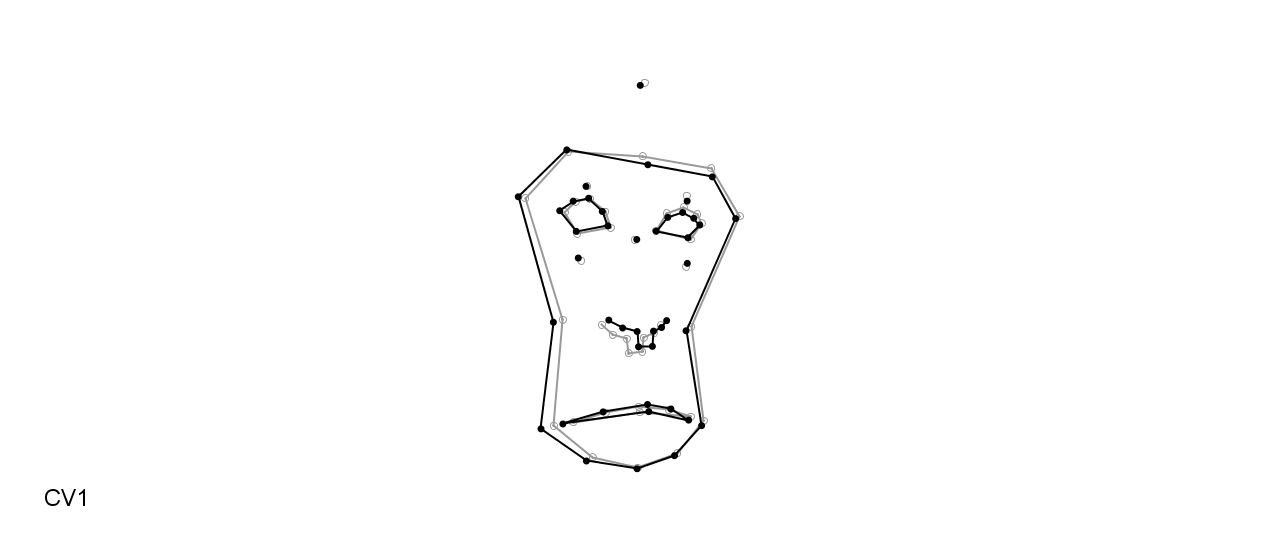

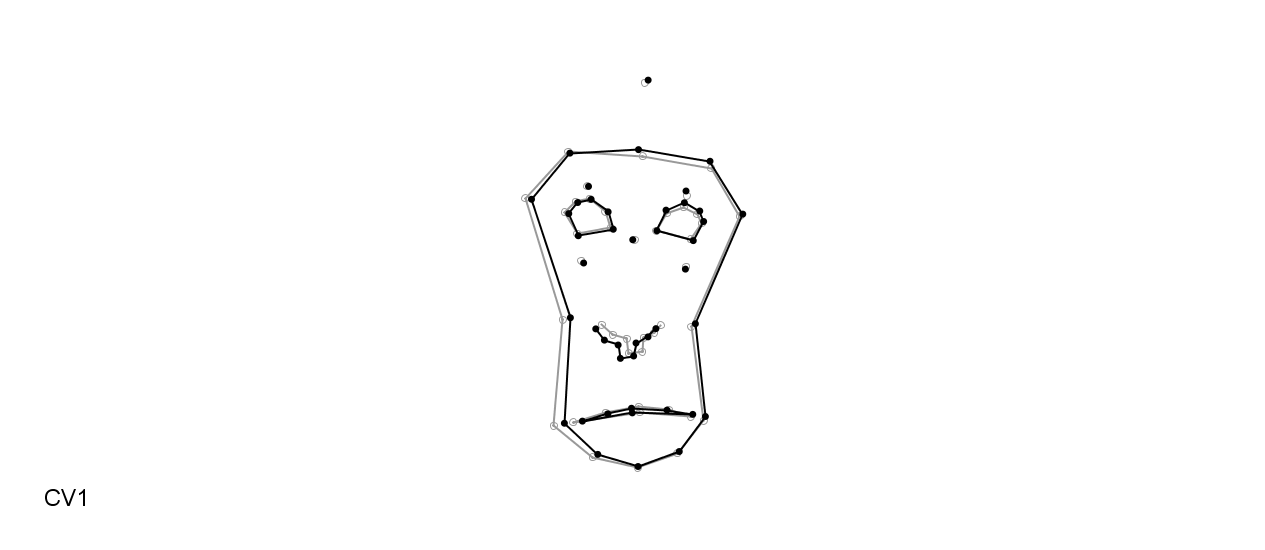


(14)

Pre

D1


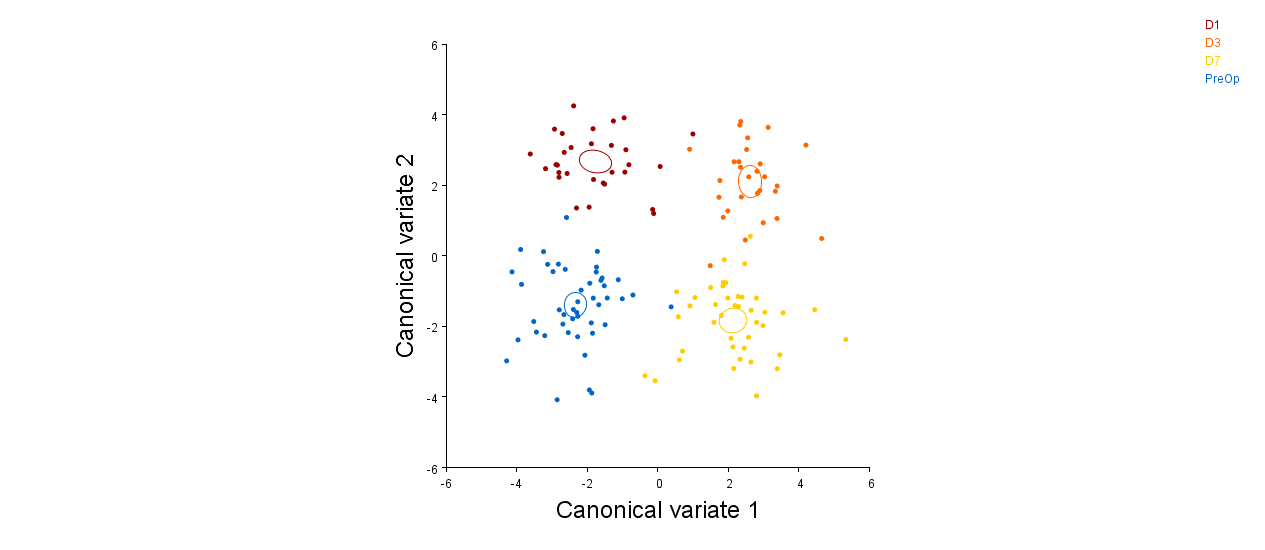

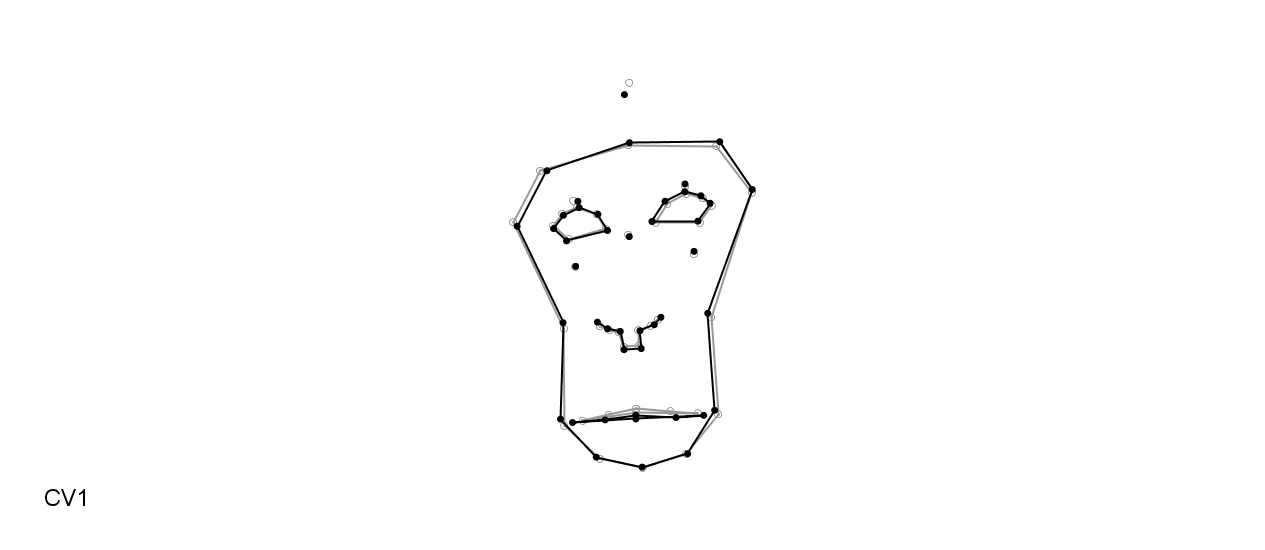

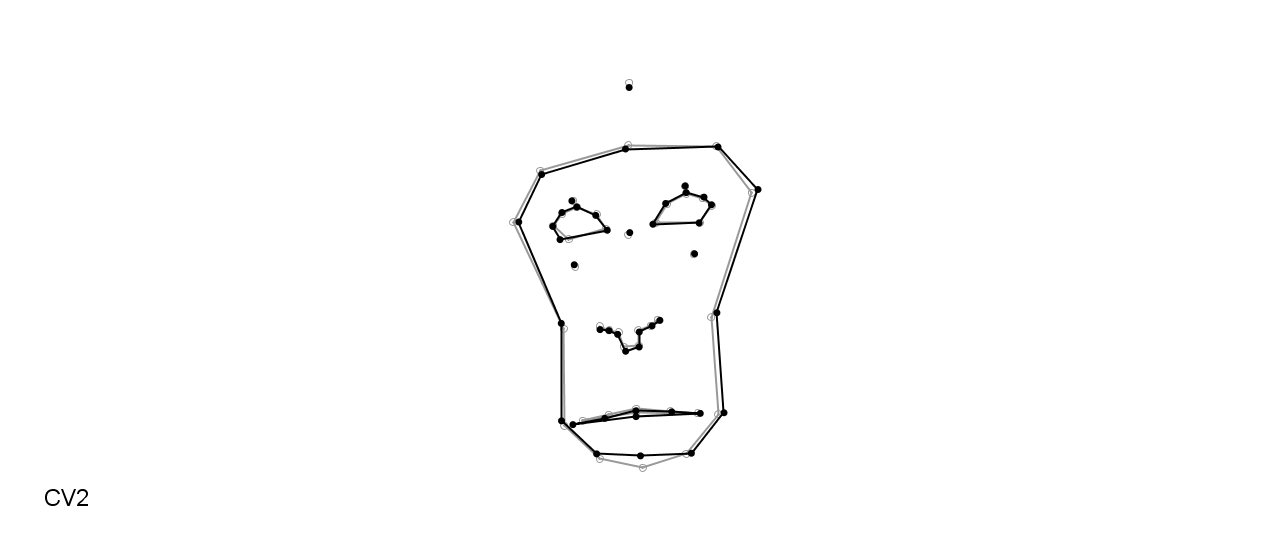

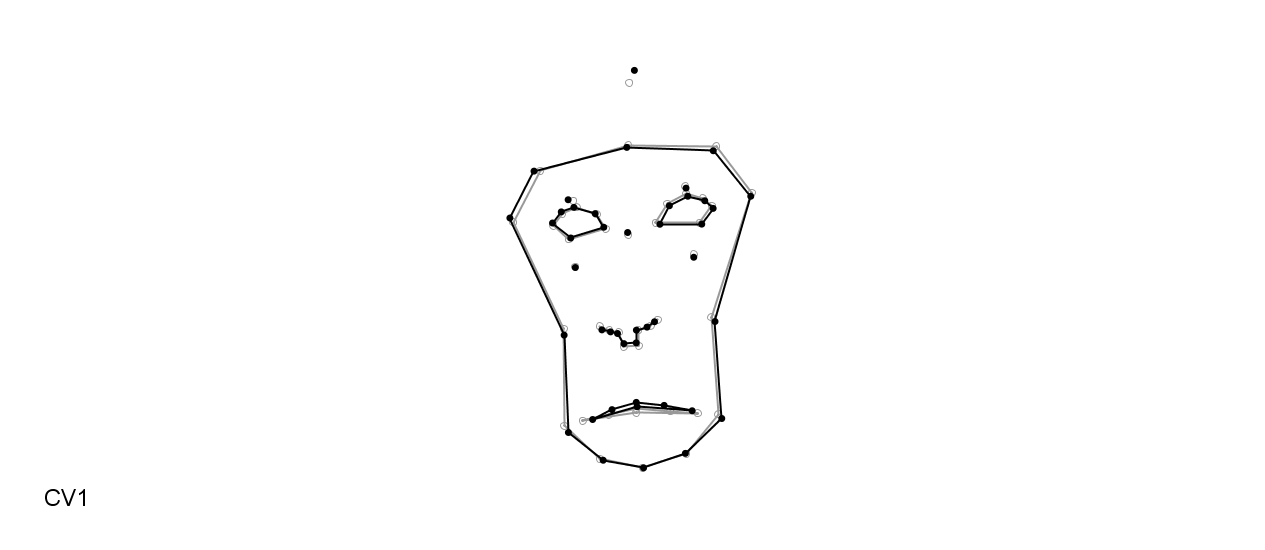

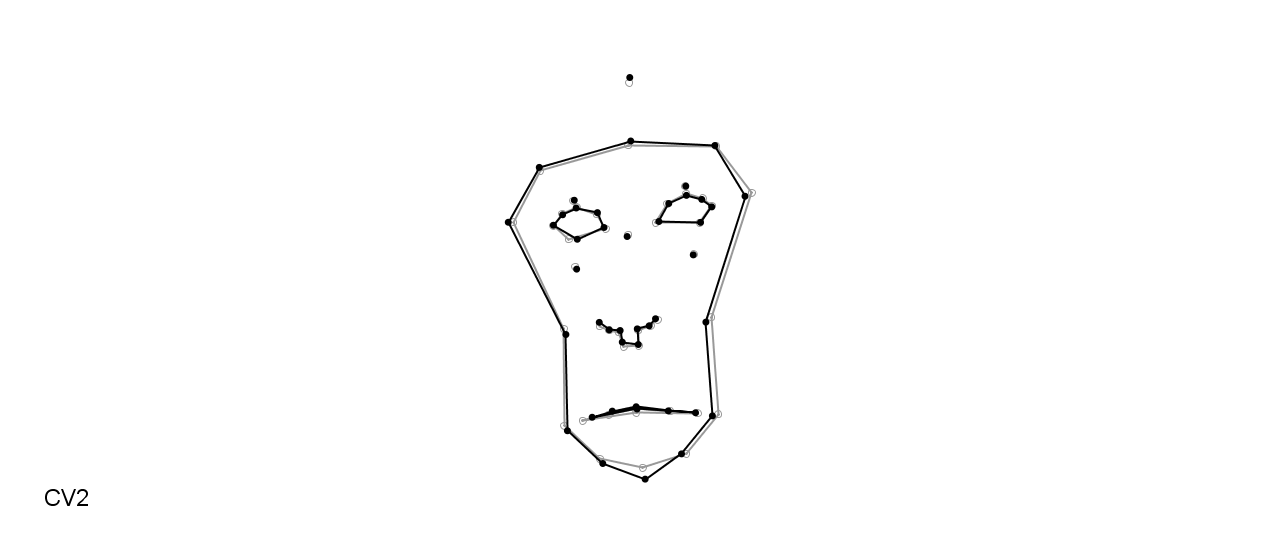


(13)

Pre

D1

D3

D7

**Supplementary Table 3. Individual changes between Pre to D1 according to the CVA plots.**

| Individual | Orbital tightening | Tension of lips | Separation of lips | Smoothed  philtral region |
| --- | --- | --- | --- | --- |
| 1 | X | X | ● | X |
| 2 | ○ | ○ | X | X |
| 3 | ○ | ● | X | X |
| 4 | X | ● | X | ○ |
| 5 | ● | ○ | ● | ○ |
| 6 | ● | ○ | X | X |
| 7 | X | X | X | X |
| 8 | ○ | ○ | X | X |
| 9 | X | ○ | X | X |
| 10 | X | X | ○ | X |
| 11 | ○ | ○ | X | X |
| 12 | ● | ○ | X | X |
| 13 | ● | X | X | X |
| 14 | ● | ● | ○ | X |
| Symbol key: Present ●, Moderately present ○, Not present X | | | | |
